# Correction: Mental health of UK Members of Parliament in the House of Commons: a cross-sectional survey

**DOI:** 10.1136/bmjopen-2018-027892corr1

**Published:** 2019-07-10

Poulter D, Votruba N, Bakolis I, *et al*.Mental health of UK Members of Parliament in the House of Commons: a cross-sectional survey. *BMJ Open* 2019;9:e027892. doi: 10.1136/bmjopen-2018-027892

This article was previously published with an error.

The statement ‘DP and NV are joint first authors’ was missing.

