# Supplementary material for: Mental health of UK Members of Parliament in the House of Commons: a cross-sectional survey
Source: BMJ Open. 2019 Jul 1;9(7):e027892. doi: 10.1136/bmjopen-2018-027892 (PMC6609039; doi:10.1136/bmjopen-2018-027892)
Supplement: Supplementary data [file bmjopen-2018-027892supp001.pdf]

Supplementary File (Online)

1. FULL LIST OF QUESTIONS

UKMPH Survey 2016: list of demographic questions

1. What age group are you?
    - Age 21 to 30
    - Age 31 to 40
    - Age 41 to 50
    - Age 51 to 60
    - Age 61 to 70
    - Age 70 +

2. How long have you been a Westminster MP?
    - Less than 5 years
    - 5 to 10 years
    - 11 to 15 years
    - 16 to 20 years
    - 21 to 25 years
    - More than 25 years

3. What is your highest level of educational attainment?
    - GCSE / O Level
    - A Level / Scottish Higher
    - Vocational Qualifications (BTEC, NVQ, HNC etc)
    - Undergraduate Degree (BA, BSc, or equivalent)
    - Post Graduate (MA, MSC, or equivalent)
    - Doctorate (PhD or equivalent)

4. What is your gender?
    - Male
    - Female

5. Do you have a job / role outside of Parliament?
    - Yes - Paid
    - Yes - Unpaid
    - No

UKMPH Survey 2016: List of questions on inhouse mental health services

6. Do you know how to access Mental Health Support through the Parliamentary Health and Wellbeing Service?
    - Yes
    - No

7. Does the Parliamentary Health and Wellbeing Service currently offer enough support to meet your mental health needs?
    - Yes
    - Somewhat
    - No

8. Would you be happy to approach your Party Whip's office if you were experiencing mental health problems?
    - Yes
    - No

9. Would you be happy to discuss with other MPs if you were experiencing mental health problems?
    - Yes
    - No

2. TABLE S1

Table S1: Descriptive characteristics of the 12 item GHQ (GHQ-12) and the four different predetermined HSE 2014 occupational and sociodemographic comparator groups (EN, CM, AM, HIG) - for Males

	n	WP	n	WP	n	WP	n	WP	n	WP
		**MP**		**EN**		**CM**		**AM**		**HIG**
		**95% CI**		**95% CI**		**95% CI**		**95% CI**		**95% CI**
**Age**										
21-30	2	0.00<br>0.00 to 0.01	515	0.22<br>0.20 to 0.23	26	0.19<br>0.13 to 0.27	35	0.17<br>0.12 to 0.23	32	0.18<br>0.13 to 0.25
31-40	15	0.10<br>0.06 to 0.16	558	0.17<br>0.16 to 0.19	28	0.12<br>0.08 to 0.17	46	0.14<br>0.10 to 0.18	59	0.23<br>0.18 to 0.29
41-50	27	0.31<br>0.22 to 0.42	702	0.19<br>0.18 to 0.20	56	0.22<br>0.17 to 0.28	74	0.19<br>0.16 to 0.24	57	0.19<br>0.15 to 0.25
51-60	31	0.49<br>0.38 to 0.60	606	0.16<br>0.15 to 0.17	38	0.17<br>0.13 to 0.23	66	0.20<br>0.15 to 0.24	43	0.14<br>0.10 to 0.18
61-70	15	0.10<br>0.06 to 0.17	632	0.14<br>0.13 to 0.15	51	0.17<br>0.13 to 0.22	78	0.18<br>0.14 to 0.22	45	0.13<br>0.10 to 0.18
70 +	2	0.00<br>0.00 to 0.01	565	0.12<br>0.11 to 0.13	37	0.12<br>0.09 to 0.16	60	0.13<br>0.10 to 0.16	46	0.13<br>0.09 to 0.17
**Educational attainment**										
NVQ4/NVQ5/ Degree	0	0.79<br>0.69 to 0.87	931	0.28<br>0.26 to 0.30	98	0.42<br>0.35 to 0.49	122	0.34<br>0.29 to 0.40	214	0.77<br>0.72 to 0.82
Higher ed below degree	72	0.09<br>0.04 to 0.17	524	0.13<br>0.12 to 0.14	29	0.13<br>0.09 to 0.19	46	0.13<br>0.09 to 0.17	29	0.08<br>0.06 to 0.12
NVQ3/GCE A Level	8	0.03<br>0.01 to 0.08	504	0.16<br>0.15 to 0.17	36	0.16<br>0.11 to 0.22	58	0.18<br>0.14 to 0.23	20	0.08<br>0.05 to 0.13
NVQ2/GCE O Level	3	0.10<br>0.05 to 0.19	631	0.18<br>0.17 to 0.20	41	0.17<br>0.13 to 0.23	66	0.18<br>0.14 to 0.23	13	0.04<br>0.02 to 0.07
NVQ1/CSE other grade	9	N/A	190	0.05<br>0.04 to 0.06	5	0.03<br>0.01 to 0.07	9	0.03<br>0.01 to 0.06	2	0.01<br>0.00 to 0.02
Foreign/other	0	N/A	9	0.00<br>0.00 to 0.01	0	0.09<br>0.06 to 0.14	1	0.01<br>0.00 to 0.04	0	0.01<br>0.00 to 0.03
No qualification	0	N/A	768	0.20<br>0.19 to 0.21	26	N/A	56	0.14<br>0.10 to 0.18	4	N/A
**GHQ -12**										
**Item 1: Have you recently been able to concentrate on whatever you're doing?**										
Better than usual	2	0.02<br>0.00 to 0.10	100	0.04<br>0.03 to 0.05	5	0.03<br>0.01 to 0.07	9	0.03<br>0.01 to 0.06	7	0.03<br>0.01 to 0.06
Same as usual	61	0.68<br>0.56 to 0.77	2746	0.87<br>0.85 to 0.88	193	0.89<br>0.83 to 0.94	290	0.90<br>0.85 to 0.93	239	0.92<br>0.87 to 0.95
Less than usual	26	0.26<br>0.18 to 0.37	284	0.08<br>0.07 to 0.09	12	0.06<br>0.03 to 0.11	18	0.06<br>0.04 to 0.10	14	0.05<br>0.03 to 0.09
Much less than usual	3	0.04<br>0.01 to 0.12	40	0.01<br>0.01 to 0.02	2	0.02<br>0.00 to 0.08	3	0.02<br>0.00 to 0.06	0	N/A
**Item 2: Have you recently lost much sleep over worry?**										
Not at all	18	0.19<br>0.12 to 0.30	1211	0.38<br>0.36 to 0.40	81	0.39<br>0.32 to 0.46	128	0.39<br>0.34 to 0.45	92	0.33<br>0.28 to 0.40
No more than usual	42	0.47<br>0.36 to 0.58	1519	0.48<br>0.46 to 0.50	114	0.53<br>0.45 to 0.60	166	0.52<br>0.46 to 0.58	136	0.56<br>0.49 to 0.62
Rather more than usual	26	0.27<br>0.19 to 0.38	352	0.11<br>0.10 to 0.12	15	0.07<br>0.04 to 0.12	23	0.08<br>0.05 to 0.11	30	0.11<br>0.07 to 0.15
Much more than usual	6	0.06<br>0.03 to 0.14	89	0.03<br>0.02 to 0.03	2	0.01<br>0.00 to 0.04	3	0.01<br>0.00 to 0.04	2	0.01<br>0.00 to 0.02
**Item 3: Have you recently felt you were playing a useful part in things?**										

More so than usual	14	0.15<br>0.09 to 0.25	291	0.10<br>0.09 to 0.11	26	0.13<br>0.09 to 0.19	35	0.11<br>0.08 to 0.16	27	0.10<br>0.07 to 0.15
Same as usual	47	0.49<br>0.38 to 0.60	2533	0.80<br>0.79 to 0.82	171	0.78<br>0.71 to 0.84	257	0.78<br>0.73 to 0.83	215	0.82<br>0.77 to 0.87
Less useful than usual	28	0.31<br>0.22 to 0.42	274	0.08<br>0.07 to 0.09	15	0.09<br>0.05 to 0.15	27	0.10<br>0.07 to 0.15	16	0.07<br>0.04 to 0.11
Much less useful	3	0.04<br>0.01 to 0.13	66	0.02<br>0.01 to 0.03	0	N/A	1	0.00<br>0.00 to 0.02	2	0.01<br>0.00 to 0.03
**Item 4: Have you recently felt capable of making decisions about things?**										
More so than usual	5	0.05<br>0.02 to 0.13	231	0.08<br>0.07 to 0.10	13	0.08<br>0.04 to 0.14	20	0.07<br>0.04 to 0.11	20	0.07<br>0.05 to 0.12
Same as usual	77	0.86<br>0.76 to 0.92	2745	0.86<br>0.84 to 0.87	193	0.88<br>0.81 to 0.93	290	0.89<br>0.84 to 0.92	232	0.89<br>0.84 to 0.92
Less so than usual	10	0.09<br>0.05 to 0.18	171	0.05<br>0.04 to 0.06	6	0.04<br>0.02 to 0.09	10	0.04<br>0.02 to 0.08	8	0.04<br>0.02 to 0.08
Much less capable	0	N/A	23	0.01<br>0.00 to 0.01	0	N/A	0	N/A	0	N/A
**Item 5: Have you felt under constant strain recently?**										
Not at all	8	0.08<br>0.04 to 0.17	837	0.27<br>0.25 to 0.29	68	0.32<br>0.25 to 0.39	106	0.32<br>0.27 to 0.38	64	0.23<br>0.18 to 0.29
No more than usual	42	0.43<br>0.33 to 0.54	1773	0.56<br>0.54 to 0.58	114	0.53<br>0.45 to 0.60	168	0.52<br>0.46 to 0.58	143	0.54<br>0.47 to 0.60
Rather more than usual	33	0.38<br>0.28 to 0.50	466	0.14<br>0.13 to 0.16	27	0.15<br>0.10 to 0.21	42	0.15<br>0.11 to 0.20	49	0.21<br>0.16 to 0.28
Much more than usual	9	0.10<br>0.05 to 0.19	92	0.03<br>0.02 to 0.03	3	0.01<br>0.00 to 0.03	4	0.01<br>0.00 to 0.02	4	0.02<br>0.01 to 0.06
**Item 6: Have you recently felt you couldn't overcome your difficulties?**										
Not at all	31	0.31<br>0.22 to 0.42	1191	0.39<br>0.37 to 0.40	88	0.41<br>0.34 to 0.49	138	0.43<br>0.37 to 0.49	96	0.34<br>0.29 to 0.41
No more than usual	45	0.52 0.41 to 0.63	1680	0.52<br>0.51 to 0.54	107	0.51<br>0.43 to 0.58	155	0.49<br>0.43 to 0.54	148	0.59<br>0.52 to 0.65
Rather more than usual	14	0.15<br>0.09 to 0.24	241	0.07<br>0.06 to 0.08	15	0.07<br>0.04 to 0.12	24	0.08<br>0.05 to 0.12	16	0.07<br>0.04 to 0.11
Much more than usual	2	0.02<br>0.01 to 0.09	55	0.02<br>0.01 to 0.02	2	0.01<br>0.00 to 0.06	3	0.01<br>0.00 to 0.04	0	N/A
**Item 7: Have you recently been able to enjoy your normal day to day activities?**										
More so than usual	1	0.01<br>0.00 to 0.05	158	0.06<br>0.05 to 0.07	15	0.09<br>0.05 to 0.15	23	0.08<br>0.05 to 0.13	12	0.04<br>0.02 to 0.07
Same as usual	58	0.61<br>0.50 to 0.71	2537	0.80<br>0.78 to 0.82	174	0.79<br>0.71 to 0.85	256	0.77<br>0.71 to 0.82	220	0.84<br>0.78 to 0.88
Less so than usual	26	0.31<br>0.22 to 0.42	382	0.12<br>0.10 to 0.13	18	0.10<br>0.06 to 0.17	33	0.12<br>0.08 to 0.17	27	0.12<br>0.08 to 0.18
Much less than usual	7	0.07<br>0.03 to 0.15	88	0.02<br>0.02 to 0.03	4	0.02<br>0.01 to 0.05	8	0.02<br>0.01 to 0.05	1	0.00<br>0.00 to 0.02
**Item 8: Have you recently been able to face up to your problems?**										
More so than usual	7	0.08<br>0.04 to 0.16	154	0.06<br>0.05 to 0.07	9	0.06<br>0.03 to 0.12	15	0.06<br>0.03 to 0.10	9	0.04<br>0.02 to 0.08
Same as usual	73	0.78<br>0.68 to 0.86	2746	0.87<br>0.86 to 0.88	191	0.90<br>0.84 to 0.94	287	0.90<br>0.86 to 0.93	235	0.91<br>0.86 to 0.95
Less able than usual	12	0.14<br>0.08 to 0.24	198	0.06<br>0.05 to 0.07	6	0.03<br>0.01 to 0.06	10	0.04<br>0.02 to 0.07	12	0.05<br>0.02 to 0.09
Much less able	0	N/A	29	0.01<br>0.01 to 0.01	1	0.01<br>0.00 to 0.06	1	0.01<br>0.00 to 0.04	0	N/A
**Item 9: Have you recently been feeling unhappy and depressed?**										
Not at all	29	0.31<br>0.21 to 0.42	1263	0.41<br>0.39 to 0.43	96	0.46<br>0.38 to 0.53	151	0.48<br>0.42 to 0.54	104	0.38<br>0.31 to 0.44
No more than usual	38	0.43<br>0.32 to 0.54	1420	0.45<br>0.43 to 0.47	90	0.43<br>0.36 to 0.51	130	0.41<br>0.35 to 0.47	129	0.54<br>0.47 to 0.60
Rather more than usual	25	0.26<br>0.18 to 0.37	366	0.12<br>0.10 to 0.13	20	0.11<br>0.07 to 0.17	28	0.10<br>0.07 to 0.14	23	0.09<br>0.06 to 0.13

Much more than usual	0	N/A	75	0.02 0.02 to 0.03	1	0.00 0.00 to 0.03	3	0.01 0.00 to 0.04	0	N/A
**Item 10: Have you recently been losing confidence in yourself?**										
**Not at all**	38	0.39 0.29 to 0.51	1510	0.49 0.47 to 0.51	113	0.53 0.46 to 0.61	176	0.55 0.49 to 0.61	132	0.48 0.42 to 0.55
No more than usual	39	0.43 0.33 to 0.54	1290	0.41 0.39 to 0.43	80	0.39 0.32 to 0.47	116	0.37 0.32 to 0.43	106	0.44 0.38 to 0.51
Rather more than usual	15	0.17 0.10 to 0.28	263	0.09 0.07 to 0.10	11	0.07 0.04 to 0.13	17	0.07 0.04 to 0.12	18	0.07 0.04 to 0.12
Much more than usual	0	N/A	58	0.02 0.01 to 0.02	1	0.00 0.00 to 0.03	2	0.01 0.00 to 0.03	0	N/A
**Item 11: Have you recently been thinking of yourself as a worthless person?**										
Not at all	54	0.57 0.45 to 0.67	2128	0.69 0.67 to 0.70	152	0.72 0.65 to 0.78	231	0.73 0.67 to 0.78	184	0.70 0.64 to 0.76
No more than usual	29	0.32 0.23 to 0.43	810	0.25 0.24 to 0.27	43	0.20 0.15 to 0.27	64	0.20 0.16 to 0.26	65	0.27 0.21 to 0.34
Rather more than usual	9	0.11 0.06 to 0.20	134	0.04 0.04 to 0.05	12	0.08 0.04 to 0.14	17	0.07 0.04 to 0.11	6	0.02 0.01 to 0.05
Much more than usual	0	N/A	53	0.02 0.01 to 0.02	0	N/A	1	0.00 0.00 to 0.03	1	0.00 0.00 to 0.03
**Item 12: Have you recently been feeling reasonably happy, all things considered?**										
More so than usual	5	0.06 0.02 to 0.14	310	0.11 0.10 to 0.12	18	0.11 0.07 to 0.17	26	0.10 0.06 to 0.14	29	0.12 0.08 to 0.18
About same as usual	67	0.71 0.60 to 0.80	2510	0.80 0.78 to 0.81	175	0.82 0.75 to 0.87	266	0.83 0.77 to 0.87	215	0.83 0.77 to 0.87
Less so than usual	20	0.23 0.15 to 0.33	243	0.07 0.06 to 0.08	13	0.06 0.03 to 0.10	18	0.06 0.04 to 0.09	11	0.05 0.02 to 0.08
Much less than usual	0	N/A	59	0.02 0.01 to 0.02	1	0.01 0.00 to 0.06	4	0.02 0.01 to 0.05	1	0.00 0.00 to 0.02
**Presence of probable mental ill health**										
No evidence of probable MIH	26	0.29 0.20 to 0.40	2009	0.56 0.55 to 0.58	143	0.58 0.51 to 0.65	220	0.58 0.52 to 0.64	168	0.58 0.52 to 0.65
Less than optimal MIH	39	0.41 0.30 to 0.52	665	0.19 0.17 to 0.20	44	0.22 0.16 to 0.28	61	0.19 0.15 to 0.24	69	0.27 0.21 to 0.33
MIH	27	0.30 0.21 to 0.41	904	0.25 0.23 to 0.26	49	0.20 0.15 to 0.27	78	0.23 0.18 to 0.28	45	0.15 0.11 to 0.20

Weighted proportion (WP) with the corresponding 95% Confidence Intervals (CI).

Key: MP: Member of Parliament Sample; EN: English Population (HSE 2014); CM: Corporate Managers (HSE 2014); AM: All managers (HSE 2014); HIG: high-income group (HSE 2014).

3. TABLE S2

Table S2: Descriptive characteristics of the 12 item GHQ (GHQ-12) and the four different predetermined HSE 2014 occupational and sociodemographic comparator groups (EN, CM, AM, HIG) - for Females

	n	WP	n	WP	n	WP	n	WP	n	WP
		**MP**		**EN**		**CM**		**AM**		**HIG**
		**95% CI**		**95% CI**		**95% CI**		**95% CI**		**95% CI**
**Age**										
21-30	2	0.00<br>0.00 to 0.01	681	0.20<br>0.19 to 0.22	21	0.13<br>0.08 to 0.21	36	0.13<br>0.09 to 0.19	26	0.21<br>0.14 to 0.29
31-40	8	0.08<br>0.04 to 0.17	784	0.17<br>0.16 to 0.19	45	0.16<br>0.12 to 0.21	72	0.17<br>0.14 to 0.21	55	0.32<br>0.25 to 0.40
41-50	17	0.32<br>0.20 to 0.46	845	0.18<br>0.17 to 0.19	66	0.24<br>0.19 to 0.29	83	0.20<br>0.16 to 0.24	30	0.18<br>0.12 to 0.25
51-60	21	0.54<br>0.39 to 0.68	726	0.16<br>0.15 to 0.17	51	0.21<br>0.16 to 0.28	80	0.21<br>0.17 to 0.26	21	0.12<br>0.08 to 0.18
61-70	5	0.06<br>0.02 to 0.14	681	0.13<br>0.12 to 0.14	44	0.15<br>0.11 to 0.19	71	0.16<br>0.13 to 0.20	20	0.11<br>0.07 to 0.17
70 +	1	0.00<br>0.00 to 0.01	722	0.15<br>0.14 to 0.16	32	0.11<br>0.08 to 0.15	55	0.12<br>0.10 to 0.16	11	0.06<br>0.03 to 0.11
**Educational attainment**										
NVQ4/NVQ5/Degree	0	N/A	1106	0.27<br>0.25 to 0.28	84	0.33<br>0.27 to 0.40	115	0.30<br>0.25 to 0.35	14<br>1	0.88<br>0.82 to 0.92
Higher ed below degree	47	0.05<br>0.01 to 0.17	483	0.10<br>0.09 to 0.11	39	0.15<br>0.11 to 0.20	58	0.14<br>0.11 to 0.18	5	0.03<br>0.01 to 0.06
NVQ3/GCE A Level	3	0.02<br>0.01 to 0.09	678	0.17<br>0.16 to 0.18	37	0.16<br>0.11 to 0.24	61	0.17<br>0.13 to 0.23	6	0.04<br>0.02 to 0.08
NVQ2/GCE O Level	2	0.01<br>0.00 to 0.07	878	0.19<br>0.18 to 0.21	58	0.22<br>0.17 to 0.27	88	0.21<br>0.17 to 0.25	6	0.03<br>0.02 to 0.08
NVQ1/CSE other grade	2	N/A	125	0.03<br>0.02 to 0.03	7	0.02<br>0.01 to 0.05	9	0.02<br>0.01 to 0.04	1	0.01<br>0.00 to 0.04
Foreign/other	0	N/A	95	0.02<br>0.02 to 0.02	4	0.01<br>0.00 to 0.03	9	0.02<br>0.01 to 0.04	2	0.01<br>0.00 to 0.04
No qualification	0	N/A	1060	0.22<br>0.21 to 0.24	30	0.10<br>0.07 to 0.15	57	0.13<br>0.10 to 0.17	2	0.01<br>0.00 to 0.03
**GHQ -12**										
**Item 1: Have you recently been able to concentrate on whatever you're doing?**										
Better than usual	3	0.05<br>0.01 to 0.15	123	0.03<br>0.02 to 0.04	10	0.04<br>0.02 to 0.07	15	0.04<br>0.02 to 0.06	3	0.02<br>0.01 to 0.06
Same as usual	32	0.61<br>0.46 to 0.74	3327	0.83<br>0.82 to 0.85	201	0.86<br>0.81 to 0.90	312	0.87<br>0.83 to 0.90	13<br>2	0.87<br>0.81 to 0.92
Less than usual	14	0.25<br>0.14 to 0.39	487	0.12<br>0.11 to 0.13	26	0.11<br>0.07 to 0.15	35	0.09<br>0.07 to 0.13	15	0.10<br>0.06 to 0.17
Much less than usual	5	0.09<br>0.04 to 0.21	63	0.01<br>0.01 to 0.02	0	N/A	0	N/A	1	0.01<br>0.00 to 0.04
**Item 2: Have you recently lost much sleep over worry?**										

Not at all	6	0.12 0.05 to 0.26	1123	0.28 0.27 to 0.30	65	0.27 0.21 to 0.34	98	0.26 0.22 to 0.32	38	0.25 0.18 to 0.33
No more than usual	24	0.45 0.31 to 0.60	2054	0.51 0.50 to 0.53	132	0.56 0.49 to 0.63	204	0.57 0.52 to 0.63	84	0.56 0.48 to 0.64
Rather more than usual	12	0.23 0.13 to 0.37	683	0.17 0.16 to 0.18	36	0.15 0.11 to 0.20	53	0.14 0.11 to 0.18	25	0.17 0.12 to 0.24
Much more than usual	12	0.19 0.10 to 0.33	151	0.03 0.03 to 0.04	5	0.02 0.01 to 0.05	8	0.02 0.01 to 0.04	4	0.02 0.01 to 0.06
**Item 3: Have you recently felt you were playing a useful part in things?**										
More so than usual	13	0.28 0.16 to 0.43	385	0.10 0.09 to 0.11	32	0.18 0.12 to 0.27	48	0.16 0.12 to 0.23	12	0.09 0.05 to 0.15
Same as usual	20	0.38 0.25 to 0.53	3163	0.79 0.78 to 0.81	191	0.76 0.68 to 0.83	291	0.77 0.71 to 0.82	124	0.80 0.72 to 0.86
Less useful than usual	15	0.26 0.15 to 0.40	351	0.08 0.08 to 0.09	11	0.05 0.03 to 0.08	20	0.05 0.03 to 0.08	14	0.10 0.06 to 0.17
Much less useful	6	0.09 0.03 to 0.20	91	0.02 0.02 to 0.03	3	0.01 0.00 to 0.04	3	0.01 0.00 to 0.03	1	0.01 0.00 to 0.05
**Item 4: Have you recently felt capable of making decisions about things?**										
More so than usual	4	0.07 0.03 to 0.19	278	0.07 0.06 to 0.08	16	0.06 0.04 to 0.10	22	0.06 0.04 to 0.09	8	0.07 0.03 to 0.13
Same as usual	41	0.78 0.65 to 0.88	3417	0.85 0.83 to 0.86	210	0.89 0.84 to 0.92	323	0.89 0.86 to 0.92	135	0.88 0.81 to 0.93
Less so than usual	7	0.10 0.04 to 0.21	273	0.07 0.06 to 0.08	11	0.04 0.02 to 0.08	17	0.04 0.03 to 0.07	8	0.05 0.03 to 0.10
Much less capable	2	0.04 0.01 to 0.17	43	0.01 0.01 to 0.01	1	0.01 0.00 to 0.04	1	0.00 0.00 to 0.02	0	N/A
**Item 5: Have you felt under constant strain recently?**										
Not at all	1	0.02 0.00 to 0.13	941	0.24 0.23 to 0.25	62	0.24 0.19 to 0.31	88	0.23 0.19 to 0.28	30	0.20 0.14 to 0.28
No more than usual	18	0.34 0.22 to 0.49	2201	0.55 0.53 to 0.57	129	0.55 0.47 to 0.62	206	0.57 0.52 to 0.63	93	0.63 0.54 to 0.70
Rather more than usual	20	0.39 0.26 to 0.54	726	0.18 0.17 to 0.19	42	0.19 0.14 to 0.26	60	0.17 0.13 to 0.22	26	0.16 0.11 to 0.22
Much more than usual	15	0.25 0.15 to 0.40	133	0.03 0.02 to 0.03	4	0.02 0.01 to 0.05	8	0.02 0.01 to 0.04	2	0.01 0.00 to 0.06
**Item 6: Have you recently felt you couldn't overcome your difficulties?**										
Not at all	10	0.20 0.11 to 0.34	1468	0.37 0.35 to 0.39	95	0.39 0.32 to 0.46	140	0.37 0.32 to 0.43	60	0.40 0.32 to 0.48
No more than usual	31	0.56 0.41 to 0.69	2082	0.52 0.50 to 0.53	127	0.55 0.47 to 0.62	197	0.56 0.50 to 0.61	81	0.54 0.46 to 0.62
Rather more than usual	10	0.18 0.09 to 0.32	361	0.09 0.08 to 0.10	16	0.07 0.04 to 0.11	24	0.07 0.04 to 0.10	7	0.05 0.02 to 0.09
Much more than usual	3	0.06 0.02 to 0.19	88	0.02 0.02 to 0.03	0	N/A	2	0.00 0.00 to 0.02	2	0.01 0.00 to 0.06
**Item 7: Have you recently been able to enjoy your normal day to day activities?**										
More so than usual	5	0.08 0.03 to 0.20	218	0.06 0.05 to 0.07	20	0.13 0.07 to 0.22	24	0.09 0.06 to 0.16	11	0.08 0.04 to 0.15
Same as usual	30	0.58 0.43 to 0.71	3112	0.78 0.76 to 0.79	184	0.74 0.66 to 0.81	288	0.77 0.71 to 0.82	124	0.82 0.75 to 0.88
Less so than usual	10	0.17	542	0.13	29	0.11	45	0.12	13	0.08

		0.09 to 0.31		0.12 to 0.14		0.08 to 0.16		0.09 to 0.15		0.05 to 0.14
Much less than usual	9	0.17 0.08 to 0.30	137	0.03 0.03 to 0.04	5	0.02 0.01 to 0.05	6	0.02 0.01 to 0.04	3	0.02 0.01 to 0.06
**Item 8: Have you recently been able to face up to your problems?**										
More so than usual	2	0.04 0.01 to 0.17	186	0.05 0.04 to 0.06	10	0.07 0.03 to 0.16	15	0.06 0.03 to 0.12	8	0.06 0.03 to 0.13
Same as usual	45	0.83 0.69 to 0.91	3411	0.86 0.85 to 0.87	213	0.89 0.81 to 0.94	323	0.90 0.84 to 0.93	137	0.90 0.83 to 0.94
Less able than usual	7	0.13 0.06 to 0.26	312	0.08 0.07 to 0.08	9	0.04 0.02 to 0.08	17	0.05 0.03 to 0.07	5	0.03 0.01 to 0.07
Much less able	0	N/A	43	0.01 0.01 to 0.01	0	N/A	0	N/A	1	0.01 0.00 to 0.05
**Item 9: Have you recently been feeling unhappy and depressed?**										
Not at all	14	0.26 0.16 to 0.41	1583	0.40 0.38 to 0.41	117	0.49 0.42 to 0.56	167	0.46 0.40 to 0.52	64	0.42 0.34 to 0.50
No more than usual	21	0.38 0.25 to 0.53	1699	0.43 0.42 to 0.45	88	0.40 0.33 to 0.48	141	0.42 0.36 to 0.47	73	0.49 0.41 to 0.58
Rather more than usual	19	0.35 0.23 to 0.50	545	0.13 0.12 to 0.15	24	0.10 0.07 to 0.15	42	0.11 0.08 to 0.15	11	0.07 0.04 to 0.13
Much more than usual	0	N/A	131	0.03 0.03 to 0.04	2	0.01 0.00 to 0.03	4	0.01 0.00 to 0.03	3	0.02 0.01 to 0.06
**Item 10: Have you recently been losing confidence in yourself?**										
Not at all	15	0.29 0.17 to 0.44	1682	0.42 0.40 to 0.44	119	0.52 0.45 to 0.59	173	0.49 0.43 to 0.55	69	0.45 0.37 to 0.54
No more than usual	26	0.49 0.35 to 0.63	1689	0.43 0.41 to 0.44	95	0.41 0.35 to 0.48	145	0.41 0.36 to 0.47	68	0.45 0.37 to 0.53
Rather more than usual	13	0.22 0.13 to 0.36	476	0.12 0.11 to 0.13	13	0.05 0.03 to 0.09	29	0.08 0.06 to 0.11	14	0.10 0.06 to 0.17
Much more than usual	0	N/A	112	0.03 0.02 to 0.04	4	0.02 0.01 to 0.04	7	0.02 0.01 to 0.04	0	N/A
**Item 11: Have you recently been thinking of yourself as a worthless person?**										
Not at all	32	0.60 0.45 to 0.73	2561	0.64 0.63 to 0.66	171	0.74 0.68 to 0.80	249	0.71 0.65 to 0.75	101	0.67 0.58 to 0.74
No more than usual	15	0.29 0.17 to 0.44	1069	0.27 0.26 to 0.29	52	0.23 0.18 to 0.29	90	0.26 0.21 to 0.31	42	0.27 0.20 to 0.35
Rather more than usual	7	0.11 0.05 to 0.24	244	0.06 0.05 to 0.07	4	0.02 0.01 to 0.04	9	0.02 0.01 to 0.05	7	0.05 0.02 to 0.11
Much more than usual	0	N/A	80	0.02 0.02 to 0.02	3	0.01 0.00 to 0.04	5	0.01 0.00 to 0.03	1	0.01 0.00 to 0.05
**Item 12: Have you recently been feeling reasonably happy, all things considered?**										
More so than usual	11	0.19 0.10 to 0.32	388	0.10 0.09 to 0.11	27	0.15 0.10 to 0.23	40	0.13 0.09 to 0.19	10	0.07 0.04 to 0.12
About same as usual	29	0.54 0.40 to 0.68	3123	0.79 0.77 to 0.80	189	0.79 0.71 to 0.85	287	0.79 0.74 to 0.84	131	0.86 0.80 to 0.91
Less so than usual	14	0.27 0.16 to 0.42	368	0.09 0.08 to 0.10	12	0.05 0.03 to 0.08	24	0.06 0.04 to 0.09	9	0.06 0.03 to 0.11
Much less than usual	0	N/A	78	0.02 0.02 to 0.03	3	0.01 0.00 to 0.04	3	0.01 0.00 to 0.03	1	0.01 0.00 to 0.05

**Presence of probable mental ill health**										
No evidence of probable MIH	9	0.19<br>0.10 to 0.34	2247	0.51<br>0.49 to 0.52	147	0.57<br>0.51 to 0.64	226	0.58<br>0.53 to 0.63	86	0.52<br>0.44 to 0.60
Less than optimal MIH	23	0.40<br>0.27 to 0.54	955	0.22<br>0.20 to 0.23	53	0.19<br>0.15 to 0.24	79	0.19<br>0.15 to 0.23	48	0.29<br>0.22 to 0.36
MIH	22	0.41<br>0.27 to 0.56	1237	0.28<br>0.26 to 0.29	59	0.24<br>0.18 to 0.30	92	0.23<br>0.19 to 0.28	29	0.20<br>0.14 to 0.27

Weighted proportion (WP) with the corresponding 95% Confidence Intervals (CI).

Key: MP: Member of Parliament Sample; EN: English Population (HSE 2014); CM: Corporate Managers (HSE 2014); AM: All managers (HSE 2014); HIG: high-income group (HSE 2014).

4. TABLE S3

Table S3. Crude and adjusted associations of mental health in relation to job status (having a job outside the parliament vs. not) of members of the parliament

GHQ-12 Items (n=146)	Crude		Adjusted±	
	**OR**	**95%CI**	**OR**	**(95% CI)**
Have you recently been able to concentrate on whatever you're doing?	0·6	0.23 to 1.57	0.74	0.27 to 2.04
Have you recently lost much sleep over worry?	0·64	0.26 to 1.58	0.73	0.28 to 1.90
Have you recently felt you were playing a useful part in things?	1.52	0.70 to 3.28	1.62	0.70 to 3.74
Have you recently felt capable of making decisions about things?	0·98	0.37 to 2.56	1.17	0.42 to 3.27
Have you felt under constant strain recently?	0·59	0.26 to 1.34	0.71	0.32 to 1.59
Have you recently felt you couldn't overcome your difficulties?	0·74	0.36 to 1.50	0.87	0.41 to 1.85
Have you recently been able to enjoy your normal day to day activities?	1.01	0.43 to 2.37	0.96	0.36 to 2.57
Have you recently been able to face up to your problems	1.04	0.37 to 2.93	0.98	0.36 to 2.69
Have you recently been feeling unhappy and depressed?	0·66	0.31 to 1.41	0.82	0.35 to 1.92
Have you recently been losing confidence in yourself?	1.02	0.37 to 2.69	1.29	0.46 to 3.60
Have you recently been thinking of yourself as a worthless person?	1.01	0.41 to 2.43	1.2	0.45 to 3.21
Presence of Common Mental Disorders	0.77	0.47 to 1.26	0.82	0.49 to 1.36
	**MD**	**95%CI**	**MD**	**95%CI**
**Total Score of GHQ to 12**	-.61	-3.06 to 1.84	-0·07	-2.44 to 2.31

Crude and Adjusted Odds Ratio (ORs) and Mean Difference (MD) with corresponding 95% Confidence Intervals (95% CI). Inverse probability weights were used with reference to the total number of the members of the parliament. All models were adjusted for age, sex and educational status
