# Supplementary material for: Mental health of UK Members of Parliament in the House of Commons: a cross-sectional survey
Source: BMJ Open. 2019 Jul 1;9(7):e027892. doi: 10.1136/bmjopen-2018-027892 (PMC6609039; doi:10.1136/bmjopen-2018-027892)
Supplement: Author's manuscript [file bmjopen-2018-027892.draft_revisions.pdf]

BMJ Open is committed to open peer review. As part of this commitment we make the peer review history of every article we publish publicly available.

When an article is published we post the peer reviewers' comments and the authors' responses online. We also post the versions of the paper that were used during peer review. These are the versions that the peer review comments apply to.

The versions of the paper that follow are the versions that were submitted during the peer review process. They are not the versions of record or the final published versions. They should not be cited or distributed as the published version of this manuscript.

BMJ Open is an open access journal and the full, final, typeset and author-corrected version of record of the manuscript is available on our site with no access controls, subscription charges or pay-per-view fees (http://bmjopen.bmj.com).

If you have any questions on BMJ Open's open peer review process please email info.bmjopen@bmj.com

Mental health of UK Members of Parliament in the House of Commons: a cross-sectional survey

Journal:	*BMJ Open*
Manuscript ID	bmjopen-2018-027892
Article Type:	Research
Date Submitted by the Author:	13-Nov-2018

Complete List of Authors:	Poulter, Daniel; House of Commons<br>Votruba, Nicole; King’s College London, Institute of Psychiatry, Psychology and Neuroscience;<br>Bakolis, Ioannis; King\'s College London, Department of Biostatistics and Health Informatics & Health Services and Population Research Department, Institute of Psychiatry, Psychology and Neuroscience<br>Debell, Frances; South London and Maudsley NHS Foundation Trust<br>Das-Munshi, Jayati; King's College London, Institute of Psychiatry, Section of Epidemiology, Department of Health Service & Population Research<br>Thornicroft, Graham; institute of psychiatry, health service and population research

Keywords:	MENTAL HEALTH, United Kingdom, policy making, stigma, Members of Parliament, MP

SCHOLARONE™
Manuscripts

Mental health of UK Members of Parliament in the House of Commons: a cross-sectional survey

Daniel Poulter[1]*, Nicole Votruba[2]*, Ioannis Bakolis[3],

Frances Debell[4], Jayati Das-Munshi[5] and Graham Thornicroft[6]

November 2018

* Joint first authors

Affiliations

[1] House of Commons, UK Parliament, House of Commons, London SW1A 0AA, UK; South London and Maudsley, Denmark Hill, London SE5 8AZ, UK; King's College London Policy Institute, 22 Kingsway, London, WC2B 6LE, UK, Dr Daniel Poulter, Visiting Professor

[2] Health Service & Population Research Department, Institute of Psychiatry, Psychology and Neuroscience, King's College London, De Crespigny Park, London SE5 8AF, UK, Nicole Votruba, Researcher

[3] Department of Biostatistics and Health Informatics & Health Services and Population Research Department, Institute of Psychiatry, Psychology and Neuroscience, King's College London, De Crespigny Park, London SE5 8AF, UK, Dr Ioannis Bakolis, Lecturer

[4] South London and Maudsley, Denmark Hill, London SE5 8AZ, UK, Dr Frances Debell

[5] Health Service & Population Research Department, Institute of Psychiatry, Psychology and Neuroscience, King's College London, De Crespigny Park, London SE5 8AF, UK, South London & Maudsley NHS Trust, UK, Dr Jayati Das-Munshi, Clinical Scientist Fellow (Honorary Consultant)

[6] Centre for Global Mental Health, Institute of Psychiatry, Psychology and Neuroscience, King's College London, De Crespigny Park, London SE5 8AF, UK, Sir Graham Thornicroft, Professor of Community Psychiatry

For peer review only - http://bmjopen.bmj.com/site/about/guidelines.xhtml

For peer review only

Correspondence to: Nicole Votruba Nicole.votruba@kcl.ac.uk +44 (0) 207 848 0619

Word count main text (excl. abstract, references, tables, boxes, figures): 3900

Key words: mental health, United Kingdom, policy making, stigma, Members of Parliament, MP

For peer review only

ABSTRACT

Objectives The purpose of this study was to assess: (i) overall mental health of Members of Parliament (MPs) of the 56th UK House of Commons; and (ii) awareness among MPs of the mental health support services available to them.

Design Anonymous, self-completed, online cross-sectional survey, conducted in December 2016.

Setting The 56th UK House of Commons.

Participants All 650 members of the 56th UK House of Commons were invited; 146 MPs (23%) completed the survey.

Outcomes The General Health Questionnaire-12 was used to assess age- and sex- standardised prevalence of probable common mental disorders, and results were compared to a nationally representative survey, the Health Survey for England 2014 (HSE). Core demographic questions, MPs' awareness of available mental health services, their willingness to discuss mental health issues with whips or with fellow MPs, and the effects of employment outside parliament, were assessed.

Results Comparison of the MP respondents with the HSE comparator groups found that MPs have higher rates of mental health problems (age/sex- standardised prevalence of probable CMDs in surveyed MPs 34%; (95% CI: 27% to 42%) versus 17%; (95% CI: 13% to 21%) HSE). 55% of MPs did not know how to access mental health support in the House of Commons, and 52% would not discuss their mental health with party whips, or other MPs (48%). No association was found for holding employment outside of parliament.

Conclusions MPs have higher rates of mental health problems, compared to the whole English population, as well as with comparable professional and occupational groups. Most MPs are unaware of mental health support services, or how to access them. There is a need for MPs to have better awareness of, and access to, mental health support services. Further in-depth research is needed to assess the mental health of MPs and Parliamentary staff.

For peer review only

STRENGTHS AND LIMITATIONS OF THIS STUDY

- This is a unique study where for the first-time the mental health in MPs has been assessed using structured validated scales.
- This study is also the first assessment of how far MPs are aware of the mental health services of the Parliamentary Health and Wellbeing Service, and how to access this service.
- We have also assessed for the first time the willingness of MPs to discuss any mental health issues with whips or with fellow MPs.
- The survey had a low response rate which may be related to the stigma surrounding mental illness, and the nature of the policymaker role which is associated to a stressful work schedule and heavy public exposure.

INTRODUCTION

There is a long history of public debate about the mental health of politicians, including discussion of the potential psychiatric diagnoses of notable individuals active in political life.[1-9] Research to date has considered some related questions, such as the harassment and stalking of politicians.[10-13] Yet, little has been published on the actual mental health or mental illness of MPs. To date, no quantitative, ethically-approved surveys have been conducted of Members of Parliament (MPs) in the UK Parliament, to assess their mental health, and to assess their awareness of support and treatment services.

Some factors in the UK political system may additionally influence MPs and their mental health:

- The UK Parliament permits MPs to hold employment outside Parliament in addition to their roles as elected representatives.
- In the UK parliament, “whips” are appointed officials in each political party who are charged with organising their party’s parliamentary business and ensuring party discipline amongst MPs.

For peer review only - http://bmjopen.bmj.com/site/about/guidelines.xhtml

- A confidential in-house service is provided within Parliament for MPs and peers, called the Parliamentary Health and Wellbeing Service, to support their occupational health and wellbeing.

The UK Parliamentary Mental Health (UKPMH) study aims to: (i) assess the overall mental health of MPs by drawing comparisons with a nationally representative survey in England, and with comparator socio-demographic and occupational groups within the survey; and (ii) assess awareness among MPs of the mental health support services available to them.

The principal research question was: What is the prevalence of common mental disorders among MPs? Additional questions were asked to inform the research study, to find out how far MPs are aware of mental health services that can assist them with mental health problems, and if they are willing to discuss their mental health with party whips or other MPs. This study tested the following hypothesis: The occurrence of Common Mental Disorders (CMDs) is higher among MPs compared to the general population or specific sociodemographic, professional and occupational comparator groups.

METHODS

Study design and participants

We conducted an anonymised, online self-completed survey at the House of Commons in December 2016. The inclusion criteria for participation was: membership of the 56th UK Parliament, House of Commons; and providing written, informed consent. We followed the STROBE guidelines for observational studies for the reporting of our cross-sectional study.[14] No age limits were defined, except to be elected to Parliament someone must be over 18 years old. Participants were identified via email, and via an invitation letter to participate sent out through the internal post to all MPs in the 56th UK Parliament. The survey took place between 5 and 31 December 2016. Initially, in November 2016 a letter was sent to all 650 members of the House of Commons to make them aware of the study. In early December, a letter including a web link to an online survey with an individual access code was sent out via to all MPs internal post, and via email. Efforts were taken to promote participation and maximise

For peer review only

response rates in the survey. A study information sheet explaining the purpose of the study, and instructions for the online questionnaire, as well as two reminder emails were sent out with clear descriptions of encrypted data collection and protection measures.

Ethics and data protection

At all times throughout the study preparation, conduct and analysis, particular consideration and care has been given to the specific, sensitive study context, and to the potential vulnerability of participants, namely the risk of sensationalised coverage should any individual be identifiable. Ethics approval for the study was obtained in September 2016 from King's College London Ethics Committee (reference number: HR-16/17-3118). Efforts were taken to limit distress and secure confidentiality for the participants. To ensure full confidentiality no personal identifiers were collected, and identifiers were removed if provided. All participants were provided with contact information for the Parliamentary Health and Wellbeing Service in the introductory letter and via the online survey in case any participants were experiencing distress at the time of the survey.

Health survey for England comparator groups

Data for the comparator groups were elicited from the Health Survey for England (HSE) 2014. The HSE is an annual survey which uses a multi-stage stratified design to sample nationally representative random cross section of the population of England each year. Participants are visited by an interviewer who collects demographic and socio-economic data, and information on health and health-related behaviours. A detailed description of the HSE has been reported elsewhere.[15] From the HSE, we identified four comparison groups: total population of England in the HSE England population (EN), corporate managers in England (CM), all managers in England (AM), and those in high income groups in England (HIG). The socio-economic groups derive from a standardised questionnaire asked in the HSE to all survey respondents.

Measures of mental health

For peer review only - http://bmjopen.bmj.com/site/about/guidelines.xhtml

The General Health Questionnaire (GHQ-12) was used to assess the mental health of respondents in the UKPMH sample and the HSE 2014. The self-completed 12-item GHQ-12 is one of the most extensively used screening instruments for common mental disorders, measured by a 4-point Likert scale (ranging from 'less than usual' to 'much more than usual') across twelve items.[15, 16]

Scoring of the GHQ-12 for the present study was done in the original bi-modal method as developed by Goldberg.[17] Specifically, each symptom was scored either 0 if 'not at all present' or present 'no more than usual', or 1 for symptoms that were present 'rather more than usual' or 'much more than usual'). The scoring method allowed for total scores to range from 0 to 12. No formal threshold exists for identifying probable mental ill health, with optimal values likely to be specific to the population under study. However, in line with the previous HSE survey, MP's total scores are grouped according to three categories: 0 (indicating no evidence of probable mental ill health), 1 to 3 (indicating less than optimal mental health), and 4 or more (indicating probable psychological disturbance or mental ill health.[15, 16]

The GHQ-12 has been extensively validated across international settings for screening and detection of the common mental disorders.[18] In previous work, at an optimal cut-point $\geq$4 on the total score of the GHQ-12 was found to have a sensitivity of 84.6% and specificity of 89.3% when assessed against *International Classification of Mental Disorders (ICD-10) and the Diagnostic Statistical Manuals-IV (DSM-IV),* diagnoses derived from the Composite International Diagnostic Interview (CIDI-PC) for the common mental disorders (e.g. including depression, dysthymia, generalised anxiety disorder, panic disorder and other related conditions)[18], when assessed in a UK setting.

A technical error in the administration of the questionnaire caused a lack of indication for respondents of the 4th option (much more/much less than usual) on GHQ-12 items 8, 9, 10, 11, 12. However, this has no impact on the total scores of GHQ-12 for each participant, as the third and fourth option are grouped together in the bi-modal scoring.

Covariates

Core demographic questions were obtained from the UKPMH study sample: Age (categorised into five groups: 21 to 30; 31 to 40; 41 to 50; 51 to 60; 61 to 70, >70 years), sex (female or male), and educational

For peer review only - http://bmjopen.bmj.com/site/about/guidelines.xhtml

status (GCSE/ O level, A Level, Vocational Qualifications, Undergraduate Degree, Post Graduate Degree, Doctorate), as well as years serving as MP. MPs were also asked if they were aware of the mental health services available to them, as well as their willingness to discuss their mental health with their Whips and other MPs (full list of questions in Supplementary File).

Statistical analyses

All statistical analyses were performed using STATA 14.1. Within the UKPMH sample, a descriptive analysis was undertaken first to determine the distribution of each item of the GHQ-12 and of socio-demographic characteristics, awareness of mental health services, and willingness to discuss any mental health issues with whips or with fellow MPs.

Our study MP sample is subject to "unit non-response" as 22.4% of the participants have completed the survey. To address this issue, we employed inverse probability weighting (IPW)[19] in our analysis, where weights are used to rebalance the set of complete cases within the MP sample to make it representative of the whole English population; we used the weighted sample of the HSE 2014. Age-sex standardised proportion estimates were calculated i) for each item of the GHQ-12, and ii) for the presence of probable mental ill health. We compared i) each item of the GHQ-12, and ii) the three combined categories derived from the total score of the GHQ-12 that indicate the presence of probable mental ill health of the MP sample with a range of socio-demographic groups (the English population (EN), corporate managers (CM), all managers (AM), and with high income groups (HIG) in England) derived from HSE 2014.

Non-parametric tests (chi-square) and parametric tests (t-test for unequal sample sizes) were employed to explore potential differences in the proportion estimates between UKPMH and HSE 2014 samples.

Cross-sectional associations of whether an MP had an additional employment outside Parliament with each different item of the GHQ-12 and with the three combined categories (indicating no evidence of probable mental ill health, less than optimal mental health, probable psychological disturbance or mental ill health) were explored with the use of ordinal logistic regression models. Results were expressed as increased risk (odds ratio and corresponding 95% confidence intervals) of being in a

For peer review only - http://bmjopen.bmj.com/site/about/guidelines.xhtml

highest category of each item of the GHQ-12 for those MPs with a work role outside parliament were compared to those without such an external role.

In addition, linear regression models were employed to explore the mean difference in the GHQ-12 total scores for those MPs who had additional employment outside Parliament, and for those who did not. All models were adjusted for the following potential confounders identified a priori: age, sex and educational status. Age-sex standardised inverse probability weights were employed for all linear and ordinal regression models.

Patient and Public Involvement

Daniel Poulter, MP, was involved at all stages of the study and is co-author of the paper. Other parliamentarians and staff of the Parliamentary Health and Wellbeing Service were consulted at the planning and design stages, as well as at the interpretations of the findings and dissemination stages of the study.

RESULTS

Questionnaires were returned by 146 respondents (22.5%) of the total of 650 MPs. Median time in completing the survey was 4 minutes (IQR: 3 to 5). Most respondents were male (63%), with an undergraduate (44%) or a postgraduate degree (36%) or Doctorate (2%), and most were between 41 and 60 years old (66%), or did not work outside parliament (81%) (see Table 1).

Table 1: Demographic characteristics of UKPMH participants

	MP sample (N=146)	Total Health Survey for England sample (N=7871)
	n (%)	n (%)
Below 40 years old	27 (18%)	4014 (51%)
Female	54 (36%)	4385 (55%)
Higher education degree	119 (82%)	888 (11.3%)
Knowledge on how to access to mental health support	65 (45%)	n/a
Unaware of parliamentary well-being service	96 (67%)	n/a
Willing to discuss mental health problems with whips	70 (48%)	n/a
Willing to discuss mental health problems with other MPs	76 (52%)	n/a
Presence of CMD (according to ≥4 cut point on the GHQ-12 total score)	49 (33%)	2902 (27%)

For peer review only - http://bmjopen.bmj.com/site/about/guidelines.xhtml

Mental health of MPs and the HSE 2014 comparator groups

Table 2 presents weighted proportion estimates and corresponding 95% confidence intervals of the UKPMH sample and the four different predetermined HSE 2014 occupational and sociodemographic comparator groups (EN, CM, AM, HIG). For each item of the GHQ-12, the UKPMH sample presented a higher weighted proportion of participants who had lower levels of concentration, were losing sleep because of worry, were feeling less useful, were less capable of making decisions, and were feeling under constant strain, compared to the four HSE 2014 occupational and sociodemographic comparison groups (p-values of chi-square test <0.001). In addition, a higher weighted proportion of MPs could not overcome difficulties, were less able to enjoy normal day to day activities, were less able to face up to their problems, reported losing confidence in themselves, or feeling unhappy and depressed, and more individual MPs considered themselves to be a worthless person (p-values of chi-square test <0.001). Compared to the HSE 2014 predetermined occupational and sociodemographic comparator groups, a higher weighted proportion of the MPs also reported being less able to feel reasonably happy (p-values of chi-square test <0.001).

When we compared the weighted proportions of the three combined categories derived for the GHQ-12 total score that indicate the presence of probable mental ill health between the UKPMH and HSE 2014 samples, we found that a higher proportion of MPs had probable mental ill health (weighted proportion: 34%; 95% CI: 27%, 42%)), compared with EN (weighted proportion: 26%; 95% CI: 25%, 27%), CM (weighted proportion: 22%; 95% CI: 18%, 26%), AM (weighted proportion: 23%; 95% CI: 20%, 27%) and HIG (weighted proportion: 17%; 95% CI: 13% to 21%) (p-values of chi-square test <0.001) (see Table 2 and Figure 1).

Table 2: Descriptive characteristics of the 12 item GHQ (GHQ-12), age, sex and educational qualifications of the UKPMH sample.

	n	WP<br>95%CI	n	WP<br>95% CI	n	WP<br>95% CI	n	WP<br>95% CI	n	WP<br>95% CI
	MP		EN		CM		AM		HIG	
**Item 1: Have you recently been able to concentrate on whatever you're doing?**										
Better than usual	5	0.03<br>0.01 to 0.07	223	0.035<br>0.03 to 0.04	15	0.03<br>0.02 to 0.05	24	0.03<br>0.02 to 0.05	10	0.03<br>0.01 to 0.05
Same as usual	93	0.66<br>0.57 to 0.74	6073	0.85<br>0.84 to 0.86	394	0.88<br>0.84 to 0.91	602	0.88<br>0.85 to 0.91	371	0.9<br>0.87 to 0.93

Less than usual	40	0.26<br>0.19 to 0.34	771	0.1<br>0.10 to 0.11	38	0.08<br>0.06 to 0.11	53	0.08<br>0.06 to 0.10	29	0.07<br>0.05 to 0.10
Much less than usual	8	0.05<br>0.02 to 0.11	103	0.01<br>0.01 to 0.02	2	0.01<br>0.00 to 0.04	3	0.01<br>0.00 to 0.03	1	0.005<br>0.00 to 0.01
**Item 2: Have you recently lost much sleep over worry?**										
Not at all	24	0.18<br>0.12 to 0.26	2334	0.33<br>0.32 to 0.34	146	0.33<br>0.28 to 0.38	226	0.33<br>0.29 to 0.37	130	0.3<br>0.26 to 0.35
No more than usual	66	0.47<br>0.38 to 0.56	3573	0.5<br>0.49 to 0.51	246	0.54<br>0.49 to 0.59	370	0.55<br>0.50 to 0.59	220	0.56<br>0.51 to 0.61
Rather more than usual	38	0.26<br>0.19 to 0.34	1035	0.14<br>0.13 to 0.15	51	0.11<br>0.08 to 0.14	76	0.11<br>0.09 to 0.14	55	0.13<br>0.10 to 0.16
Much more than usual	18	0.1<br>0.06 to 0.16	240	0.03<br>0.02 to 0.04	7	0.02<br>0.01 to 0.03	11	0.02<br>0.01 to 0.03	6	0.01<br>0.00 to 0.03
**Item 3: Have you recently felt you were playing a useful part in things?**										
More so than usual	27	0.19<br>0.13 to 0.27	676	0.10<br>0.09 to 0.11	58	0.16<br>0.12 to 0.21	83	0.14<br>0.11 to 0.18	39	0.10<br>0.07 to 0.13
Same as usual	67	0.46<br>0.38 to 0.55	5696	0.8<br>0.79 to 0.81	362	0.77<br>0.72 to 0.81	548	0.78<br>0.74 to 0.81	339	0.82<br>0.77 to 0.85
Less useful than usual	43	0.3<br>0.22 to 0.39	625	0.08<br>0.07 to 0.09	26	0.07<br>0.05 to 0.10	47	0.08<br>0.06 to 0.10	30	0.08<br>0.05 to 0.12
Much less useful	9	0.05<br>0.02 to 0.11	157	0.02<br>0.01 to 0.03	3	0.005<br>0.00 to 0.02	4	0.005<br>0.00 to 0.02	3	0.01<br>0.00 to 0.02
**Item 4: Have you recently felt capable of making decisions about things?**										
More so than usual	9	0.06<br>0.03 to 0.11	509	0.08<br>0.07 to 0.09	29	0.07<br>0.05 to 0.11	42	0.07<br>0.05 to 0.09	28	0.07<br>0.05 to 0.10
Same as usual	118	0.84<br>0.77 to 0.89	6162	0.85<br>0.84 to 0.86	403	0.88<br>0.84 to 0.91	613	0.89<br>0.86 to 0.91	367	0.89<br>0.85 to 0.92
Less so than usual	17	0.09<br>0.05 to 0.15	444	0.066<br>0.06 to 0.08	17	0.04<br>0.02 to 0.07	27	0.04<br>0.03 to 0.06	16	0.04<br>0.02 to 0.07
Much less capable	2	0.01<br>0.00 to 0.05	66	0.01<br>0.01 to 0.01	1	0<br>0.00 to 0.02	1	0<br>0.00 to 0.01	0	NA
**Item 5: Have you felt under constant strain recently?**										
Not at all	9	0.07<br>0.03 to 0.13	1778	0.25<br>0.24 to 0.27	130	0.28<br>0.24 to 0.33	194	0.28<br>0.24 to 0.31	94	0.22<br>0.18 to 0.27
No more than usual	60	0.41<br>0.33 to 0.50	3974	0.56<br>0.54 to 0.57	243	0.54<br>0.49 to 0.59	374	0.55<br>0.51 to 0.59	236	0.57<br>0.51 to 0.62
Rather more than usual	53	0.38<br>0.30 to 0.47	1192	0.16<br>0.15 to 0.17	69	0.17<br>0.13 to 0.21	102	0.16<br>0.13 to 0.20	75	0.19<br>0.15 to 0.24
Much more than usual	24	0.14<br>0.09 to 0.21	225	0.03<br>0.02 to 0.03	7	0.02<br>0.01 to 0.03	12	0.02<br>0.01 to 0.03	6	0.02<br>0.01 to 0.04
**Item 6: Have you recently felt you couldn't overcome your difficulties?**										
Not at all	41	0.29<br>0.21 to 0.37	2659	0.38<br>0.37 to 0.39	183	0.4<br>0.35 to 0.45	278	0.4<br>0.36 to 0.44	156	0.36<br>0.31 to 0.41
No more than usual	76	0.52<br>0.44 to 0.61	3762	0.52<br>0.51 to 0.53	234	0.53<br>0.47 to 0.58	352	0.52<br>0.48 to 0.56	229	0.57<br>0.52 to 0.62
Rather more than usual	24	0.16<br>0.10 to 0.23	602	0.08<br>0.08 to 0.09	31	0.07<br>0.05 to 0.10	48	0.07<br>0.05 to 0.09	23	0.06<br>0.04 to 0.09
Much more than usual	5	0.03<br>0.01 to 0.08	143	0.02<br>0.02 to 0.02	2	0.01<br>0.00 to 0.03	5	0.01<br>0.00 to 0.02	2	0<br>0.00 to 0.02
**Item 7: Have you recently been able to enjoy your normal day to day activities?**										
More so than usual	6	0.03<br>0.01 to 0.06	376	0.06<br>0.05 to 0.07	35	0.11<br>0.07 to 0.16	47	0.09<br>0.06 to 0.13	23	0.05<br>0.04 to 0.08
Same as usual	88	0.61<br>0.52 to 0.69	5649	0.79<br>0.78 to 0.80	358	0.76<br>0.71 to 0.81	544	0.77<br>0.73 to 0.81	344	0.83<br>0.79 to 0.87
Less so than usual	36	0.27<br>0.19 to 0.36	924	0.12<br>0.12 to 0.13	47	0.11<br>0.08 to 0.14	78	0.12<br>0.09 to 0.15	40	0.11<br>0.08 to 0.15
Much less than usual	16	0.10<br>0.06 to 0.16	225	0.025<br>0.02 to 0.03	9	0.02<br>0.01 to 0.04	14	0.02<br>0.01 to 0.03	4	0.01<br>0.00 to 0.02
**Item 8: Have you recently been able to face up to your problems?**										
More so than usual	9	0.07<br>0.04 to 0.13	340	0.06<br>0.05 to 0.07	19	0.06<br>0.04 to 0.11	30	0.06<br>0.04 to 0.09	17	0.05<br>0.03 to 0.08
Same as usual	118	0.80<br>0.71 to 0.86	6157	0.87<br>0.86 to 0.88	404	0.90<br>0.85 to 0.93	610	0.9<br>0.86 to 0.92	372	0.91<br>0.87 to 0.94
Less able than usual	19	0.14<br>0.08 to 0.21	510	0.07<br>0.06 to 0.07	15	0.03<br>0.02 to 0.06	27	0.04<br>0.03 to 0.06	17	0.04<br>0.02 to 0.07
Much less able	NA	NA	72	0.01<br>0.01 to 0.01	1	0.01<br>0.00 to 0.03	1	0.01<br>0.00 to 0.02	1	0.01<br>0.00 to 0.02
**Item 9: Have you recently been feeling unhappy and depressed?**										
Not at all	43	0.3	2846	0.4	213	0.47	318	0.47	168	0.39

For peer review only - http://bmjopen.bmj.com/site/about/guidelines.xhtml

		0.22 to 0.38		0.39 to 0.42		0.42 to 0.52		0.43 to 0.51		0.34 to 0.44
No more than usual	59	0.42<br>0.33 to 0.51	3119	0.44<br>0.43 to 0.45	178	0.42<br>0.37 to 0.47	271	0.41<br>0.37 to 0.46	202	0.52<br>0.47 to 0.58
Rather more than usual	44	0.29<br>0.21 to 0.37	911	0.13<br>0.12 to 0.15	44	0.1<br>0.08 to 0.14	70	0.11<br>0.08 to 0.13	34	0.08<br>0.06 to 0.11
Much more than usual	NA	NA	206	0.03<br>0.01 to 0.04	3	0.01<br>0.00 to 0.02	7	0.01<br>0.01 to 0.03	3	0.01<br>0.00 to 0.02
**Item 10: Have you recently been losing confidence in yourself?**										
Not at all	53	0.37<br>0.29 to 0.46	3192	0.45<br>0.44 to 0.47	232	0.52<br>0.47 to 0.58	349	0.52<br>0.48 to 0.56	201	0.47<br>0.42 to 0.53
No more than usual	65	0.45<br>0.36 to 0.54	2979	0.42<br>0.41 to 0.43	175	0.4<br>0.35 to 0.45	261	0.39<br>0.35 to 0.43	174	0.44<br>0.39 to 0.50
Rather more than usual	28	0.18<br>0.13 to 0.26	739	0.1<br>0.10 to 0.11	24	0.06<br>0.04 to 0.10	46	0.08<br>0.06 to 0.10	32	0.08<br>0.06 to 0.12
Much more than usual	NA	NA	170	0.02<br>0.02 to 0.03	5	0.01<br>0.00 to 0.02	9	0.015<br>0.01 to 0.02	NA	NA
**Item 11: Have you recently been thinking of yourself as a worthless person?**										
Not at all	86	0.58<br>0.49 to 0.66	4689	0.66<br>0.65 to 0.68	323	0.73<br>0.68 to 0.77	480	0.72<br>0.68 to 0.75	285	0.69<br>0.64 to 0.74
No more than usual	44	0.31<br>0.24 to 0.40	1879	0.26<br>0.25 to 0.27	95	0.22<br>0.18 to 0.26	154	0.23<br>0.20 to 0.27	107	0.27<br>0.23 to 0.32
Rather more than usual	16	0.11<br>0.06 to 0.18	378	0.05<br>0.05 to 0.06	16	0.05<br>0.03 to 0.08	26	0.05<br>0.03 to 0.07	13	0.03<br>0.02 to 0.06
Much more than usual	NA	N A NA	133	0.02<br>0.02 to 0.02	3	0.01<br>0.00 to 0.02	6	0.01<br>0.00 to 0.02	2	0.01<br>0.00 to 0.02
**Item 12: Have you recently been feeling reasonably happy, all things considered?**										
More so than usual	16	0.09<br>0.05 to 0.15	698	0.11<br>0.10 to 0.11	45	0.13<br>0.09 to 0.18	66	0.12<br>0.09 to 0.15	39	0.11<br>0.08 to 0.14
About same as usual	96	0.67<br>0.59 to 0.75	5633	0.79<br>0.78 to 0.80	364	0.8<br>0.75 to 0.85	553	0.81<br>0.77 to 0.84	346	0.84<br>0.80 to 0.88
Less so than usual	34	0.24<br>0.17 to 0.32	611	0.08<br>0.08 to 0.09	25	0.05<br>0.04 to 0.08	42	0.06<br>0.04 to 0.08	20	0.05<br>0.03 to 0.08
Much less than usual	NA	NA	137	0.02<br>0.02 to 0.02	4	0.01<br>0.00 to 0.03	7	0.01<br>0.01 to 0.03	2	0<br>0.00 to 0.02
**Presence of probable mental ill health**										
No evidence of probable mental ill health	35	0.25<br>0.18 to 0.34	4256	0.53<br>0.52 to 0.55	290	0.58<br>0.53 to 0.62	446	0.58<br>0.54 to 0.62	254	0.56<br>0.51 to 0.61
Less than optimal mental ill health	62	0.40<br>0.32 to 0.49	1620	0.2<br>0.19 to 0.21	97	0.2<br>0.17 to 0.25	140	0.19<br>0.16 to 0.22	117	0.27<br>0.23 to 0.32
Probable mental ill health	49	0.34<br>0.27 to 0.43	2141	0.26<br>0.25 to 0.27	108	0.22<br>0.18 to 0.26	170	0.23<br>0.20 to 0.27	74	0.17<br>0.13 to 0.21
Weighted proportion (WP) with the corresponding 95% Confidence Intervals (CI).										

Key: MP: Member of Parliament Sample; EN: English Population (HSE 2014); CM: Corporate Managers (HSE 2014); AM: All managers (HSE 2014); HIG: high-income group (HSE 2014).

Awareness of mental health support services

Most MPs were unaware of the mental health services provided by the Parliamentary Health and Wellbeing Service within parliament. Most MPs (55 %) did not know how to access any mental health support at Parliament (see Figure 2). When asked whether they felt the Parliamentary Health and Wellbeing Service currently offered sufficient support, a large majority of MPs (67%) were unaware of

what options are currently offered by the service. Only 23% were aware that support was sufficiently available (see Figure 3).

(Figures 2, 3, 4, 5 about here)

Willingness to disclose poor mental health

Most MPs who took part in our survey are not willing to discuss mental health problems with their party whips (52%), and only a small majority of MPs would feel able to talk with other MPs about their mental health (52%) (see Figures 4 and 5). After adjusting for age, sex and educational status, we found evidence that MPs who were willing to discuss their mental health with their party whips (adjusted OR: 0.32; 95% CI: 0.16, 0.31), or with their fellow MPs (adjusted OR: 0.57; 95% CI: 0.30, 0.99) had a reduced risk of CMD (data not shown).

Additional employment outside parliament

We found no evidence of an association between having additional employment outside Parliament with the individual GHQ-12 items or an increased total score indicating poor mental health (see Supplementary File, Table S1).

DISCUSSION

Principal findings

The main findings of this study were: 1) strong evidence to indicate that a higher proportion of MPs had poor mental health than among the general population and among the defined occupational and sociodemographic comparator groups (EN, CM, AM, HIG). 2) Most MPs were not aware of Parliamentary mental health and support services. 3) Most MPs are not willing to discuss their mental health with party whips, and only a small majority would be happy to discuss mental health issues with other MPs. 4) It appears that having employment outside Parliament, in addition to the role of MP, is not linked with increased risk for mental ill health.

For peer review only - http://bmjopen.bmj.com/site/about/guidelines.xhtml

The Parliamentary Health and Wellbeing Service is the occupational health service provided since 2013 inside the House of Commons. It aims to support all staff and MPs in developing a healthy and safe working environment and encourages them to adopt better attitudes towards their own health and mental health.[20] Despite the service being in place for almost four years, the Parliamentary Health and Wellbeing Service had reported low numbers of MPs requesting support. Our study confirms this in finding that a majority of MPs are unaware of the service or how to access it. Reasons for this might be insufficient advertising of the support options offered and location of the services, as well as anticipated stigma and discrimination among MPs[21].

Strengths and weaknesses of the study

The study has several limitations and potential biases. First, the response rate was relatively low (22.4%). Given the intense work loads of MPs, this may have been partly due to the additional work load of completing the survey, even though the median time to complete survey was 4 minutes. Also, a possible fear of being identified, stigmatisation, and the potential reputational damage associated with adverse media coverage may have influenced the response rate. We tried to reduce these biases by promoting the survey in Parliament, sending reminders, and stressing brevity, as well as the anonymity of the survey. Generally, MPs are a difficult survey population to engage, which has also been confirmed in a 2008 internal UK Parliament survey, where only 14.5% (94 MPs) responded.[22]

Secondly, it is also possible that MPs who responded to the online survey may have increased stress or mental ill health and that therefore a greater number of them were willing to complete the survey. A potential self-selection bias may therefore be present in our UKPMH sample. However, there is also a potential risk of underreporting from people who might be reluctant to take part in the study, because they are affected by mental health problems, or because of the stigma associated with the topic. We therefore feel confident that the risks of over- and underreporting are weighing up and that our findings are representative.

Respondents tended to be younger in relation to the age distribution of the total number of MPs (27% of our MPs sample vs. 16% of total MP population were below 40 years old), and more likely to be

For peer review only - http://bmjopen.bmj.com/site/about/guidelines.xhtml

female (36% female of our MPs sample vs 30% of total MPs population were female) in relation to the gender distribution of the total number of MPs and had a university degree (81% of our MPs sample vs 76% of total MP population). We did not assess marital or cohabitance status, as this would've increased the risk of identifiability of MPs, and potentially this would've also affected the response rate negatively. However, these potentially interesting confounders could be considered in future, more in-depth evaluations.

Thirdly, comparing MPs to other occupational and sociodemographic groups within a population presents challenges. We considered comparing the UKPMH sample to the UK Health and Safety Executive's Labour Force Survey (LFS), which provides annual data on rates of mental disorder by occupation.[23] However, the LFS relies on random household sampling is poorly suited to extrapolating meaningful data for a relatively tiny group 650 UK MPs. Published LFS data lacks sufficient granularity to be able to analyse the prevalence of mental disorders at an occupation-specific level, which for politicians would be 'elected officers and representatives'.[24] Given the unique features of political careers, including the diverse backgrounds from which politicians may be drawn, specific data relating to these generic occupational groupings are unlikely to be fully helpful in understanding why there is a higher burden of mental ill health. In our sample we found that having employment outside Parliament, and in addition to the role of MP, does not seem to constitute an increased risk for mental ill health. However, we regard this outcome with caution as we feel our study may be underpowered for a general statement, as most participants (81%) did not have employment outside Parliament.

Comparison of results with earlier studies

When examining UK parliamentary working hours reform, research found high levels of physical and emotional stress as a result of various aspects of political life such as additional work roles, extensive travel and job insecurity.[25] A longitudinal study in new UK MPs highlighted increased levels of stress post-election.[26] In 2008 the UK Parliament has also conducted its own informal survey regarding experience and perceptions of mental illness, which concluded that one in five MPs had a personal experience of a mental health problem, and one in three felt stigma was a barrier to openness about

For peer review only - http://bmjopen.bmj.com/site/about/guidelines.xhtml

mental health, yet no data on CMDs was collected.[22] Given that work characteristics promoting stress are associated with mental disorders,[27, 28] it may be reasonable to assume that rates of common mental disorder would be high in parliamentarians, however no rigorous assessment has previously been conducted to investigate this issue.

This is the first study of assessment of mental health in members of Parliament of the UK House of Commons using structured validated scales. Our findings indicate that MPs are more likely to experience probable mental ill health and symptoms indicative of mental distress compared to the general population and compared with similar occupational and professional groups. In addition, most MPs are not aware of mental health support offered by the Parliamentary Health and Wellbeing service, or willing to disclose to their whips or other MPs. This leaves MPs with levels of mental ill health facing a critical situation and a lack of awareness of how to access help. These findings are of considerable concern.

Interpretation of the results

A number of studies have examined media and public reactions to politicians' actual or perceived mental health problems.[29-31] In an ever more hostile media environment, poor mental health can be regarded as a factor limiting politicians in their capacities. Stigma against people with mental disorders is prevalent in all countries and sectors of society. It was not until 2013 that the UK passed the Mental Health (Discrimination) (No 2) Act 2013, which removed discriminatory provisions permitting Members of Parliament (MPs) with mental health problems to be disqualified under certain circumstances.[32] Subsequent to the Act, there have been more disclosures from politicians about personal mental health problems. However, given that our results showed that only 48% of surveyed MPs felt able to talk to their party whips, and only 52% felt able to talk to another MP about their mental health, stigma and self-stigma about mental health appears to remain a powerful barrier to seeking help and support among Members of the UK House of Commons. Our findings show an increased need for mental health support for MPs, and the need for awareness raising, stigma and self-stigma reduction, in order to facilitate access to mental health services in the House of Commons.

For peer review only - http://bmjopen.bmj.com/site/about/guidelines.xhtml

Implications for future research

This is a first, initial study into the mental health of MPs, and further in-depth research is needed to assess the raised issues, and to assess trends in the mental health of MPs over time. Our findings are only a starting point, but they reveal MPs' mental health problems and the need to properly assess them. There is a need for better promotion of mental health support, such as the Parliamentary Health and Wellbeing Service, and for additional information and support for MPs in accessing the mental health services provided. Due to their working routine and hours, MPs spend a majority of their working time far from support provided by their usual NHS services, which is why strengthening the Parliamentary Health and Wellbeing Service could offer a specifically relevant support function. Further research is also needed on mental health in other parliamentary staff to increase comparability, to identify their needs, and to evaluate their awareness of, and access to, the Parliamentary Health and Wellbeing Service and other services.

CONCLUSION AND POLICY IMPLICATIONS

MPs have a vital role to play in the UK democracy: in making and scrutinising the legislation that governs the country, as well as in representing the interests of their constituents. We have found indication that the people in these important roles experience significantly higher levels of mental ill health when compared to the general population, and when compared to other senior executive and managerial groups. The majority of MPs do not feel that they have adequate mental health support, they lack knowledge of how to access the mental health services that are available to them. Most MPs are not able to discuss their mental health problems with their whips or other MPs. Our findings indicate that better support to MPs is required to access existing mental health services, such as the one provided in the House of Commons. More research is needed to identify what the UK Parliament can do to support the mental health needs of its MPs and other staff, not least in order to ensure they can best address legislation and directives for mental health for the people they serve.

For peer review only - http://bmjopen.bmj.com/site/about/guidelines.xhtml

Acknowledgments: We would like to thank all Members of Parliament who took part in this study. In addition, we would like to thank Elaine Bryce (member of Dr Daniel Poulter's Parliamentary office), and the staff of the Parliamentary Health and Wellbeing Service for their support in this study.

Funding: This research received no specific grant from any funding agency in the public, commercial or not-for-profit sectors.

Competing interests:

NV acknowledges funding from the Economic and Social Research Council (ESRC) and National Institute for Health Research (NIHR) Collaboration for Leadership in Applied Health Research and Care South London at King's College London NHS Foundation Trust.

GT is supported by the National Institute for Health Research (NIHR) Collaboration for Leadership in Applied Health Research and Care South London at King's College London NHS Foundation Trust. The views expressed are those of the author(s) and not necessarily those of the NHS, the NIHR or the Department of Health. GT acknowledges financial support from the Department of Health via the National Institute for Health Research (NIHR) Biomedical Research Centre and Dementia Unit awarded to South London and Maudsley NHS Foundation Trust in partnership with King's College London and King's College Hospital NHS Foundation Trust. GT is supported by the European Union Seventh Framework Programme (FP7/2007-2013) Emerald project. GT also receives support from the National Institute of Mental Health of the National Institutes of Health under award number R01MH100470 (Cobalt study). GT is also supported by the UK Medical Research Council in relation the Emilia (MR/S001255/1) and Indigo Partnership (MR/R023697/1) awards.

IB is supported by the NIHR Biomedical Research Centre at South London and Maudsley NHS Foundation Trust and by the NIHR Collaboration for Leadership in Applied Health Research.

JD has a Clinician Scientist Fellowship, funded by the Health Foundation working with the Academy of Medical Sciences

For peer review only

All authors have completed the ICMJE uniform disclosure form at www.icmje.org/coi_disclosure.pdf and declare: no support from any organisation for the submitted work; no financial relationships with any organisations that might have an interest in the submitted work in the previous three years; DP is currently MP of the 57th UK Parliament and was member of the 56th UK Parliament; no other relationships or activities that could appear to have influenced the submitted work.

Copyright/license for publication: The Corresponding Author has the right to grant on behalf of all authors and does grant on behalf of all authors, a worldwide licence to the Publishers and its licensees in perpetuity, in all forms, formats and media (whether known now or created in the future), to i) publish, reproduce, distribute, display and store the Contribution, ii) translate the Contribution into other languages, create adaptations, reprints, include within collections and create summaries, extracts and/or, abstracts of the Contribution, iii) create any other derivative work(s) based on the Contribution, iv) to exploit all subsidiary rights in the Contribution, v) the inclusion of electronic links from the Contribution to third party material where-ever it may be located; and, vi) licence any third party to do any or all of the above."

Ethical approval: Ethics approval for the study was obtained in September 2016 from King's College London Ethics Committee (reference number: HR-16/17-3118).

Data sharing: No additional data available. The Health Survey for England 2014 can be accessed at: https://digital.nhs.uk/data-and-information/publications/statistical/health-survey-for-england/health-survey-for-england-2014. Due to the sensibility of the data, and in order to ensure full anonymity, confidentiality and data protection for the participants, the full survey data cannot be made accessible to the public.

Transparency declaration: The corresponding author affirms that the manuscript is an honest, accurate, and transparent account of the study being reported; that no important aspects of the study have been omitted; and that any discrepancies from the study as planned have been explained.

Contributors: DP and GT conceived the original idea for the study, which was then discussed with NV. NV coordinated the study. All authors contributed to the design of the study. NV and FD conducted

For peer review only

the literature review. DP and NV collected the data. IB conducted design and analysis of the data. NV led the writing of the manuscript, and all authors contributed and critically revised it. All authors have given their approval for the publication of this manuscript and agree to be accountable for all aspects of the work to ensure that the questions related to the accuracy or integrity of any part of the work are appropriately investigated and resolved.

For peer review only

References

1. Davidson J. Downing Street Blues: A History of Depression and Other Mental Afflictions in British Prime Ministers: McFarland 2010.
2. Fieve R. The case of dominique straus-kahn and mental disorder. *International Clinical Psychopharmacology* 2012;28:e14-e15.
3. Freedman L. Mental states and political decisions: Commentary on... Psychiatry and politicians. *The Psychiatrist* 2011;35(4):148-50.
4. Peters U. Daniel Paul Schreber, the illness of the Senate president. *Fortschritte der Neurologie-Psychiatrie* 1995;63(12):469-79.
5. Owen D. In sickness and in power: illnesses in heads of government during the last 100 years,: Methuen Publishing, London, 2008.
6. Owen LD. Hubris and nemesis in heads of government. *Journal of the Royal Society of Medicine* 2006;99(11):548-51.
7. Russell G. Psychiatry and politicians: the 'hubris syndrome'. *The Psychiatrist* 2011;35(4):140-45.
8. Sidwell B. Gaius Caligula's Mental Illness. *Classical world* 2010;103(2):183-206.
9. Vatz RE. Rhetoric and psychiatry: A Szaszian perspective on a political case study. *Current Psychology* 2006;25(3):173-81.
10. James DV, Mullen PE, Meloy JR, et al. The role of mental disorder in attacks on European politicians 1990–2004. *Acta Psychiatrica Scandinavica* 2007;116(5):334-44.
11. James DV, Sukhwal S, Farnham FR, et al. Harassment and stalking of Members of the United Kingdom Parliament: associations and consequences. *The Journal of Forensic Psychiatry & Psychology* 2016;27(3):309-30.
12. Gersons BPR, Nijdam MJ. Supporting leaders under threat and their protection. In: Parkes CM, ed. Responses to Terrorism: Can psychosocial approaches break the cycle of violence? New York: Routledge 2014:181-444.
13. Every-Palmer S, Barry-Walsh J, Pathé M. Harassment, stalking, threats and attacks targeting New Zealand politicians: A mental health issue. *Australian & New Zealand Journal of Psychiatry* 2015;49(7):634-41.
14. Equator Network. The Strengthening the Reporting of Observational Studies in Epidemiology (STROBE) Statement: guidelines for reporting observational studies [Available from: http://www.equator-network.org/reporting-guidelines/strobe/ accessed 06.11.2018.

15. Craig R, Fuller E, Mindell J. Health survey for England 2014, 2015.
16. Goldberg DP, Blackwell B. Psychiatric illness in general practice: a detailed study using a new method of case identification. *Br med J* 1970;2(5707):439-43.
17. Goldberg DP, Hillier VF. A scaled version of the General Health Questionnaire. *Psychological medicine* 1979;9(1):139-45.
18. Goldberg DP, Gater R, Sartorius N, et al. The validity of two versions of the GHQ in the WHO study of mental illness in general health care. *Psychol Med* 1997;27(1):191-7. [published Online First: 1997/01/01]
19. Hofler M, Pfister H, Lieb R, et al. The use of weights to account for non-response and drop-out. *Soc Psychiatry Psychiatr Epidemiol* 2005;40(4):291-9. doi: 10.1007/s00127-005-0882-5 [published Online First: 2005/04/19]
20. UK Parliament. New support announced for MPs with mental health problems 12 February 2013 [Available from: http://www.parliament.uk/business/committees/committees-a-z/other-committees/members-estimate/news/new-support-announced-for-mps-with-mental-health-problems/ accessed 22.02.2018.
21. Thornicroft G, Mehta N, Clement S, et al. Evidence for effective interventions to reduce mental-health-related stigma and discrimination. *The Lancet* 2016;387(10023):1123-32. doi: https://doi.org/10.1016/S0140-6736(15)00298-6
22. All-Party Parliamentary Group on Mental Health. Mental Health in Parliament: Report by the All-Party Parliamentary Group on Mental Health. 2008
23. Health and Safety Executive. Labour Force Survey data for year 2013/14-2015/16 2016.
24. Office for National Statistics. Standard Occupational Classification 2010
25. Weinberg A, Cooper CL. Workload, stress and family life in British Members of Parliament and the psychological impact of reforms to their working hours. *Stress and Health* 1999;15(2):79-87.
26. Weinberg A, Cooper C. Stress among national politicians elected to Parliament for the first time. *Stress and Health* 2003;19(2):111-17.
27. Wilhelm K, Kovess V, Rios-Seidel C, et al. Work and mental health. *Social psychiatry and psychiatric epidemiology* 2004;39(11):866-73.
28. Wang J, Lesage A, Schmitz N, et al. The relationship between work stress and mental disorders in men and women: findings from a population-based study. *Journal of Epidemiology & Community Health* 2008;62(1):42-47.
29. Altheide DL. Mental illness and the news: The Eagleton story. *Sociology & Social Research* 1977
30. Dukakis MS. Campaigns and disability: When an incumbent president questions his potential successor's mental health status during the campaign. *Politics & Life Sciences* 2015;33(2):88-92.
31. MacDonald A, Majumder RK. On the resolution and tolerance of cognitive inconsistency in another naturally occurring event: Attitudes and beliefs following the Senator Eagleton incident. *Journal of Applied Social Psychology* 1973;3(2):132-43.
32. Wykes T, Craig T. Can our politicians help to reduce stigma and discrimination?: Taylor & Francis, 2013.

For peer review only

For peer review only - http://bmjopen.bmj.com/site/about/guidelines.xhtml

List of Figures and legends

Figure 1:
Age-Sex standardised prevalence estimates and 95% Confidence Intervals of UKPMH and of specific population groups of HSE 2014 for the three different categories of Common Mental Disorders (CMD).

Key: MP: Member of Parliament Sample; EN: English Population (HSE 2014); CM: Corporate Managers (HSE 2014); AM: All managers (HSE 2014); HIG: High-income group (HSE 2014).

Figure 2:
Access to the mental health (MH) support of the Parliamentary Health and Wellbeing Service

NB: All p-values <0.001.

Figure 3:
Awareness of the mental health (MH) support of the Parliamentary Health and Wellbeing Service

NB: All p-values <0.001

Figure 4:
Willingness to talk to party whips

NB: All p-values <0.001

Figure 5:
Willingness to talk to other MPs

NB: All p-values <0.001

For peer review only

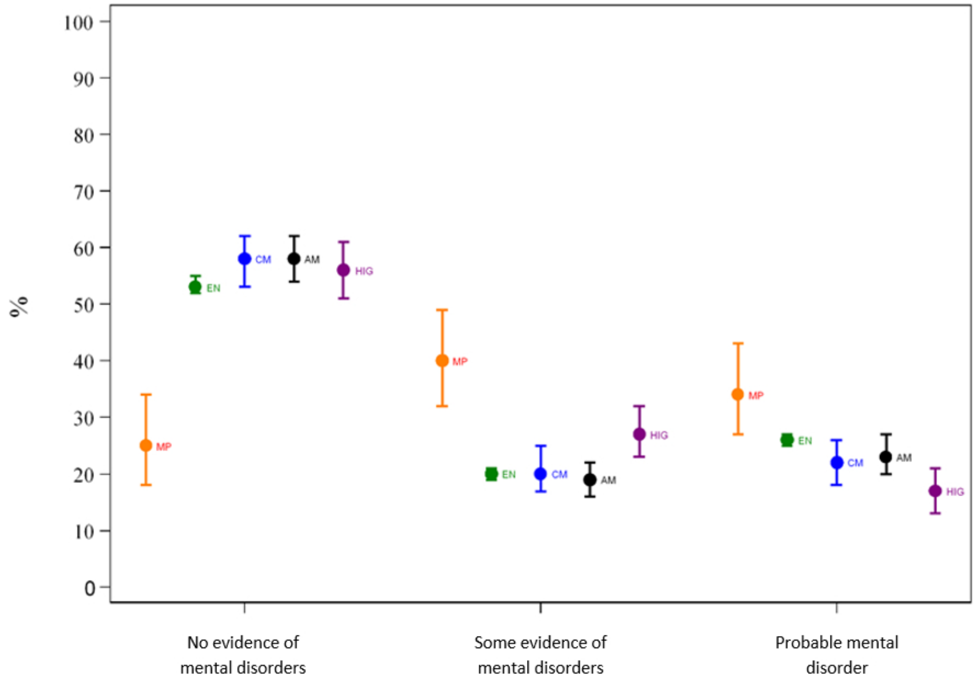

Figure 1: Age-Sex standardised prevalence estimates and 95% Confidence Intervals of UKPMH and of specific population groups of HSE 2014 for the three different categories of Common Mental Disorders (CMD).

Key: MP: Member of Parliament Sample; EN: English Population (HSE 2014); CM: Corporate Managers (HSE 2014); AM: All managers (HSE 2014); HIG: High-income group (HSE 2014).

192x141mm (120 x 120 DPI)

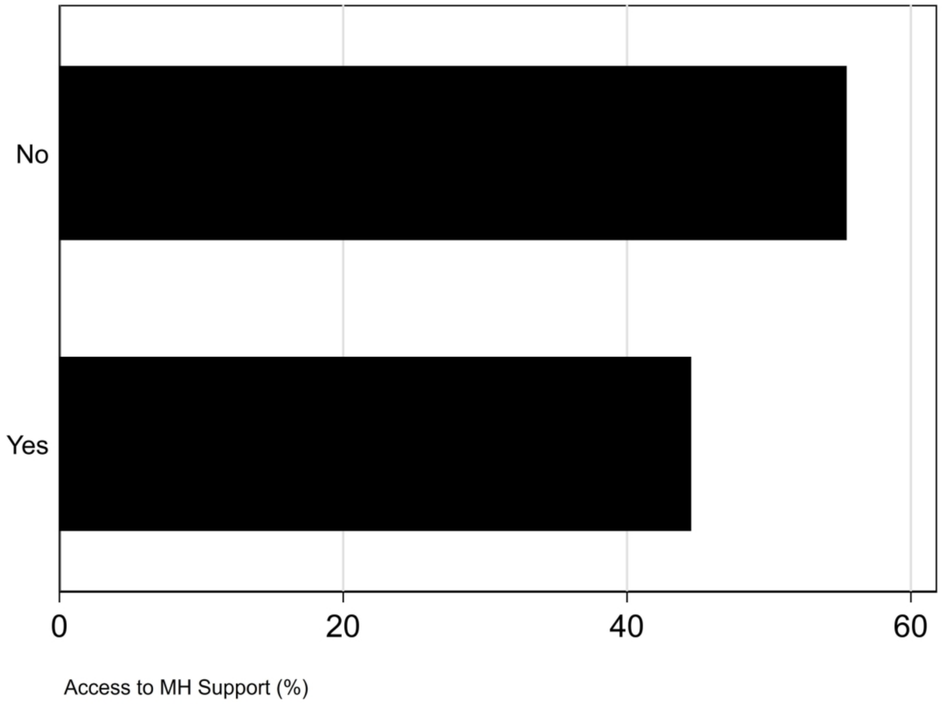

Figure 2: Access to the mental health (MH) support of the Parliamentary Health and Wellbeing Service

NB: All p-values <0.001.

169x127mm (300 x 300 DPI)

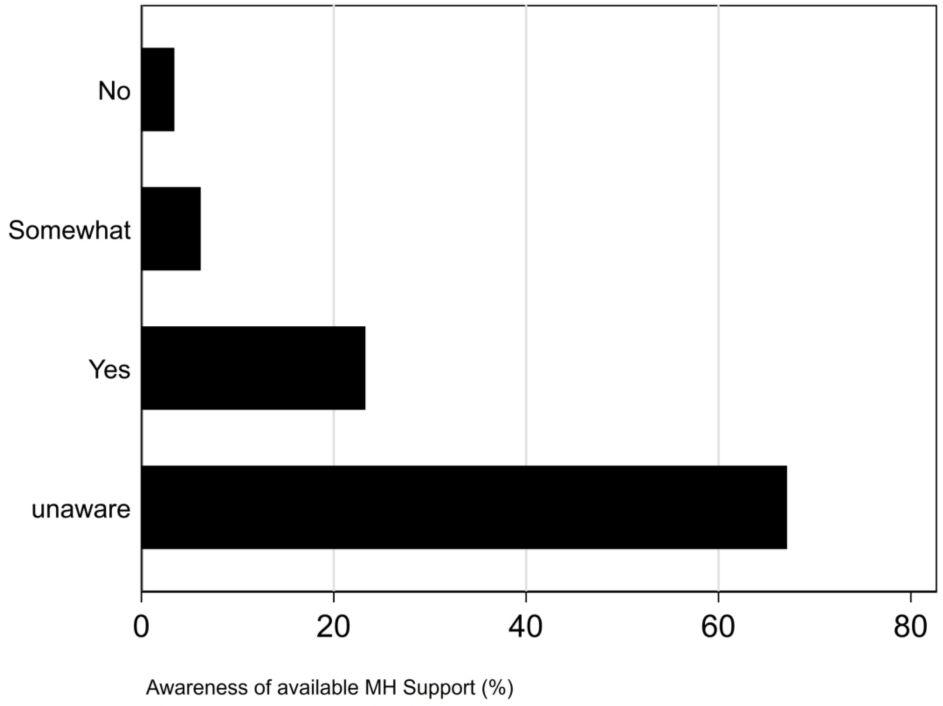

Figure 3: Awareness of the mental health (MH) support of the Parliamentary Health and Wellbeing Service

NB: All p-values <0.001.

169x127mm (300 x 300 DPI)

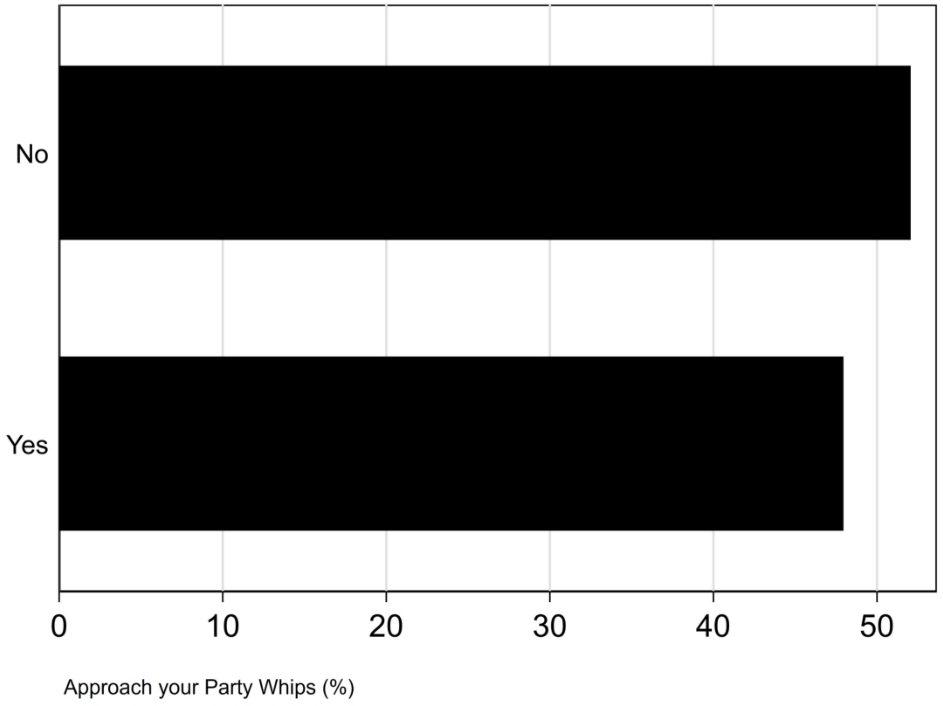

Figure 4: Willingness to talk to party whips

NB: All p-values <0.001.

169x127mm (300 x 300 DPI)

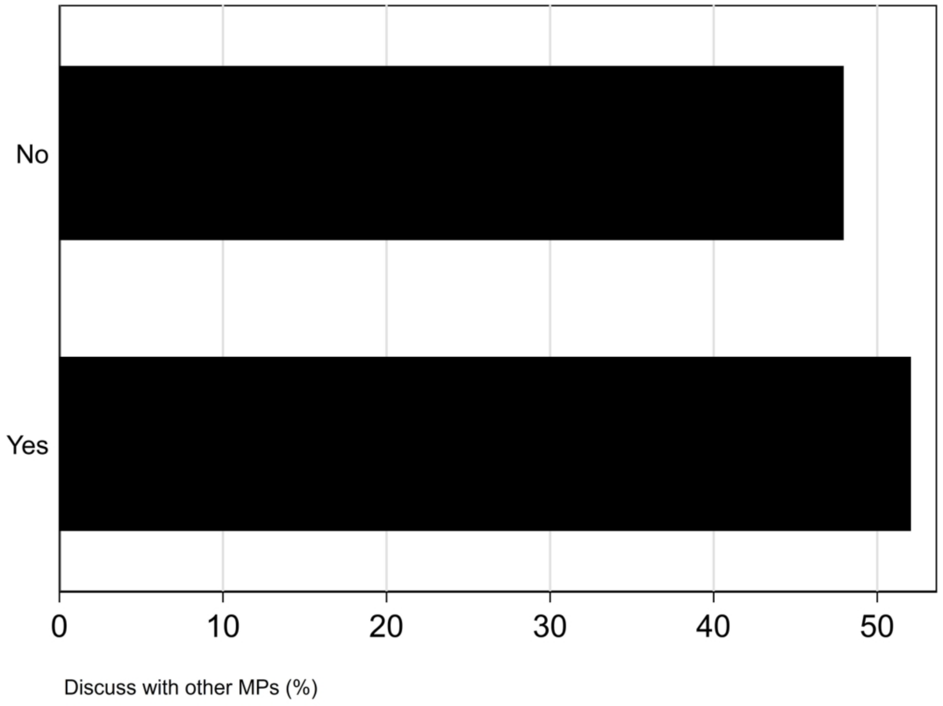

Figure 5: Willingness to talk to other MPs

NB: All p-values <0.001.

169x127mm (300 x 300 DPI)

Supplementary File (Online)

1. FULL LIST OF QUESTIONS

UKMPH Survey 2016: list of demographic questions

1. What age group are you?
    - Age 21 to 30
    - Age 31 to 40
    - Age 41 to 50
    - Age 51 to 60
    - Age 61 to 70
    - Age 70 +

2. How long have you been a Westminster MP?
    - Less than 5 years
    - 5 to 10 years
    - 11 to 15 years
    - 16 to 20 years
    - 21 to 25 years
    - More than 25 years

3. What is your highest level of educational attainment?
    - GCSE / O Level
    - A Level / Scottish Higher
    - Vocational Qualifications (BTEC, NVQ, HNC etc)
    - Undergraduate Degree (BA, BSc, or equivalent)
    - Post Graduate (MA, MSC, or equivalent)
    - Doctorate (PhD or equivalent)

4. What is your gender?
    - Male
    - Female

5. Do you have a job / role outside of Parliament?
    - Yes - Paid
    - Yes - Unpaid
    - No

For peer review only - http://bmjopen.bmj.com/site/about/guidelines.xhtml

UKMPH Survey 2016: List of questions on inhouse mental health services

6. Do you know how to access Mental Health Support through the Parliamentary Health and Wellbeing Service?
    - Yes
    - No

7. Does the Parliamentary Health and Wellbeing Service currently offer enough support to meet your mental health needs?
    - Yes
    - Somewhat
    - No

8. Would you be happy to approach your Party Whip's office if you were experiencing mental health problems?
    - Yes
    - No

9. Would you be happy to discuss with other MPs if you were experiencing mental health problems?
    - Yes
    - No

For peer review only

2. TABLE S1

Table S1. Crude and adjusted associations of mental health in relation to job status (having a job outside the parliament vs. not) of members of the parliament

GHQ-12 Items (n=146)	Crude		Adjusted[‡]	
	**OR**	**95%CI**	**OR**	**(95% CI)**
Have you recently been able to concentrate on whatever you're doing?	0·6	0.23 to 1.57	0.74	0.27 to 2.04
Have you recently lost much sleep over worry?	0·64	0.26 to 1.58	0.73	0.28 to 1.90
Have you recently felt you were playing a useful part in things?	1.52	0.70 to 3.28	1.62	0.70 to 3.74
Have you recently felt capable of making decisions about things?	0·98	0.37 to 2.56	1.17	0.42 to 3.27
Have you felt under constant strain recently?	0·59	0.26 to 1.34	0.71	0.32 to 1.59
Have you recently felt you couldn't overcome your difficulties?	0·74	0.36 to 1.50	0.87	0.41 to 1.85
Have you recently been able to enjoy your normal day to day activities?	1.01	0.43 to 2.37	0.96	0.36 to 2.57
Have you recently been able to face up to your problems	1.04	0.37 to 2.93	0.98	0.36 to 2.69
Have you recently been feeling unhappy and depressed?	0·66	0.31 to 1.41	0.82	0.35 to 1.92
Have you recently been losing confidence in yourself?	1.02	0.37 to 2.69	1.29	0.46 to 3.60
Have you recently been thinking of yourself as a worthless person?	1.01	0.41 to 2.43	1.2	0.45 to 3.21
Presence of Common Mental Disorders	0.77	0.47 to 1.26	0.82	0.49 to 1.36
	**MD**	**95%CI**	**MD**	**95%CI**
**Total Score of GHQ to 12**	-.61	-3.06 to 1.84	-0·07	-2.44 to 2.31

Crude and Adjusted Odds Ratio (ORs) and Mean Difference (MD) with corresponding 95% Confidence Intervals (95% CI). Inverse probability weights were used with reference to the total number of the members of the parliament. All models were adjusted for age, sex and educational status

For peer review only

STROBE Statement—Checklist of items that should be included in reports of ***cross-sectional studies***

Item	Item No	Recommendation	Page No
**Title and abstract**	1	(*a*) Indicate the study's design with a commonly used term in the title or the abstract	1
		(*b*) Provide in the abstract an informative and balanced summary of what was done and what was found	3
**Introduction**			
Background/rationale	2	Explain the scientific background and rationale for the investigation being reported	4-5
Objectives	3	State specific objectives, including any prespecified hypotheses	5
**Methods**			
Study design	4	Present key elements of study design early in the paper	5
Setting	5	Describe the setting, locations, and relevant dates, including periods of recruitment, exposure, follow-up, and data collection	5-6
Participants	6	(*a*) Give the eligibility criteria, and the sources and methods of selection of participants	5
Variables	7	Clearly define all outcomes, exposures, predictors, potential confounders, and effect modifiers. Give diagnostic criteria, if applicable	7
Data sources/ measurement	8*	For each variable of interest, give sources of data and details of methods of assessment (measurement). Describe comparability of assessment methods if there is more than one group	6-7
Bias	9	Describe any efforts to address potential sources of bias	5-6
Study size	10	Explain how the study size was arrived at	5, 8
Quantitative variables	11	Explain how quantitative variables were handled in the analyses. If applicable, describe which groupings were chosen and why	8-9
Statistical methods	12	(*a*) Describe all statistical methods, including those used to control for confounding	8-9
		(*b*) Describe any methods used to examine subgroups and interactions	8-9
		(*c*) Explain how missing data were addressed	8-9
		(*d*) If applicable, describe analytical methods taking account of sampling strategy	8-9
		(*e*) Describe any sensitivity analyses	8-9
**Results**			
Participants	13*	(a) Report numbers of individuals at each stage of study—eg numbers potentially eligible, examined for eligibility, confirmed eligible, included in the study, completing follow-up, and analysed	9
		(b) Give reasons for non-participation at each stage	n.a.
		(c) Consider use of a flow diagram	n.a.
Descriptive data	14*	(a) Give characteristics of study participants (eg demographic, clinical, social) and information on exposures and potential confounders	9
		(b) Indicate number of participants with missing data for each variable of interest	n.a.
Outcome data	15*	Report numbers of outcome events or summary measures	9-12
Main results	16	(*a*) Give unadjusted estimates and, if applicable, confounder-adjusted estimates and their precision (eg, 95% confidence interval). Make clear	10-12

For peer review only - http://bmjopen.bmj.com/site/about/guidelines.xhtml

		which confounders were adjusted for and why they were included	
		(*b*) Report category boundaries when continuous variables were categorized	10-12
		(*c*) If relevant, consider translating estimates of relative risk into absolute risk for a meaningful time period	N.a.
Other analyses	17	Report other analyses done—eg analyses of subgroups and interactions, and sensitivity analyses	12-13
**Discussion**			
Key results	18	Summarise key results with reference to study objectives	13-14
Limitations	19	Discuss limitations of the study, taking into account sources of potential bias or imprecision. Discuss both direction and magnitude of any potential bias	14-15
Interpretation	20	Give a cautious overall interpretation of results considering objectives, limitations, multiplicity of analyses, results from similar studies, and other relevant evidence	16
Generalisability	21	Discuss the generalisability (external validity) of the study results	15-17
**Other information**			
Funding	22	Give the source of funding and the role of the funders for the present study and, if applicable, for the original study on which the present article is based	18

*Give information separately for exposed and unexposed groups.

Note: An Explanation and Elaboration article discusses each checklist item and gives methodological background and published examples of transparent reporting. The STROBE checklist is best used in conjunction with this article (freely available on the Web sites of PLoS Medicine at http://www.plosmedicine.org/, Annals of Internal Medicine at http://www.annals.org/, and Epidemiology at http://www.epidem.com/). Information on the STROBE Initiative is available at www.strobe-statement.org.

For peer review only

BMJ Open

Mental health of UK Members of Parliament in the House of Commons: a cross-sectional survey

Journal:	*BMJ Open*
Manuscript ID	bmjopen-2018-027892.R1
Article Type:	Research
Date Submitted by the Author:	15-Mar-2019
Complete List of Authors:	Poulter, Daniel; House of Commons<br>Votruba, Nicole; King's College London, Institute of Psychiatry, Psychology and Neuroscience;<br>Bakolis, Ioannis; King\'s College London, Department of Biostatistics and Health Informatics & Health Services and Population Research Department, Institute of Psychiatry, Psychology and Neuroscience<br>Debell, Frances; South London and Maudsley NHS Foundation Trust<br>Das-Munshi, Jayati; King's College London, Institute of Psychiatry, Section of Epidemiology, Department of Health Service & Population Research<br>Thornicroft, Graham; institute of psychiatry, Health service and population research/ Centre for Global Health
**Primary Subject Heading**:	Mental health
Secondary Subject Heading:	Public health, Mental health, Health services research, Health policy, Global health
Keywords:	MENTAL HEALTH, United Kingdom, policy making, stigma, Members of Parliament, MP

SCHOLARONE™
Manuscripts

Mental health of UK Members of Parliament in the House of Commons: a cross-sectional survey

Daniel Poulter[1]*, Nicole Votruba[2]*, Ioannis Bakolis[3],

Frances Debell[4], Jayati Das-Munshi[5] and Graham Thornicroft[6]

November 2018

* Joint first authors

Affiliations

[1] House of Commons, UK Parliament, House of Commons, London SW1A 0AA, UK; South London and Maudsley, Denmark Hill, London SE5 8AZ, UK; King's College London Policy Institute, 22 Kingsway, London, WC2B 6LE, UK, Dr Daniel Poulter, Visiting Professor

[2] Health Service & Population Research Department, Institute of Psychiatry, Psychology and Neuroscience, King's College London, De Crespigny Park, London SE5 8AF, UK, Nicole Votruba, Researcher

[3] Department of Biostatistics and Health Informatics & Health Services and Population Research Department, Institute of Psychiatry, Psychology and Neuroscience, King's College London, De Crespigny Park, London SE5 8AF, UK, Dr Ioannis Bakolis, Lecturer

[4] South London and Maudsley, Denmark Hill, London SE5 8AZ, UK, Dr Frances Debell

[5] Health Service & Population Research Department, Institute of Psychiatry, Psychology and Neuroscience, King's College London, De Crespigny Park, London SE5 8AF, UK, South London & Maudsley NHS Trust, UK, Dr Jayati Das-Munshi, Clinical Scientist Fellow (Honorary Consultant)

For peer review only

[6] Centre for Global Mental Health, and Centre for Implementation Science, Institute of Psychiatry, Psychology and Neuroscience, King's College London, De Crespigny Park, London SE5 8AF, UK, Sir Graham Thornicroft, Professor of Community Psychiatry

Correspondence to: Nicole Votruba Nicole.votruba@kcl.ac.uk +44 (0) 207 848 0619

Word count main text (excl. abstract, references, tables, boxes, figures): 3900

Key words: mental health, United Kingdom, policy making, stigma, Members of Parliament, MP

For peer review only

ABSTRACT

Objectives The purpose of this study was to assess: (i) overall mental health of Members of Parliament (MPs) of the 56th UK House of Commons; and (ii) awareness among MPs of the mental health support services available to them in Parliament.

Design Anonymous, self-completed, online cross-sectional survey, conducted in December 2016.

Setting 56th UK House of Commons.

Participants All 650 members of the 56th UK House of Commons were invited to participate; 146 MPs (23%) completed the survey.

Outcomes The General Health Questionnaire-12 was used to assess age and sex standardised prevalence of probable common mental disorders (CMD). Results were compared to a nationally representative survey, the Health Survey for England 2014 (HSE). Core demographic questions, MPs' awareness of available mental health services, their willingness to discuss mental health issues with party whips and fellow MPs, and the effects of employment outside parliament, were assessed.

Results Comparison of MP respondents with HSE comparator groups found that MPs have higher rates of mental health problems (age and sex standardised prevalence of probable CMD in surveyed MPs 34% (n=49); (95% CI: 27% to 42%) versus 17%; (95% CI: 13% to 21%) in the high-income comparison group). Survey respondents were younger, more likely to be female and more educated, compared to all MPs. 77% of MPs (n=112) did not know how to access in house mental health support. 52% (n=76) would not discuss their mental health with party whips, or other MPs (48%; n=70).

Conclusions MPs in the study sample had higher rates of mental health problems than rates seen in the whole English population, or in comparable occupational groups. Most surveyed MPs are unaware of mental health support services, or how to access them. There is a need for MPs to have better awareness of, and access to, mental health support services.

For peer review only

STRENGTHS AND LIMITATIONS OF THIS STUDY

- This is a unique study where the mental health of MPs has been assessed using structured, validated scales for the first time.
- This study is also the first evaluation of MPs' awareness of the mental health support available to them from the Parliamentary Health and Wellbeing Service and how to access this service.
- This study also assessed for the first time the willingness of MPs to discuss any mental health issues with party whips or with fellow MPs.
- The survey had a relatively low response rate which may be related to the stigma associated with mental illness, and to the nature of an MP's role, which is associated with a stressful work schedule and life in the public eye.

For peer review only

INTRODUCTION

There is a public fascination with understanding the psyches of politicians and decision-makers, from ancient times to the present day, and a long history of public debate about the mental health of politicians, including discussion of the potential psychiatric diagnoses of notable individuals active in political life[1-9]. Research studies have considered some related questions, such as the harassment and stalking of politicians.[10-13] Studies have also examined media and public reactions to politicians' actual or perceived mental health problems. [14-17] Yet, little has been published on the actual mental health or mental illness of politicians. Some evidence of politicians disclosing personal mental health problems has been published, for example during the passage of the UK Mental Health (Discrimination) Act in 2013, which removed discriminatory provisions permitting disqualification of Members of Parliament with mental health problems under certain circumstances.[18].

A scoping literature search in January 2017 was conducted to understand what is known about politicians' mental health, and in particular the prevalence of common mental disorders in this group. The papers identified were largely limited to politicians in the UK, USA and Australasia. There remains a dearth of evidence on the prevalence of common mental disorders (CMDs) in politicians and how this compares to general population rates. To date, no quantitative, ethically-approved surveys have been conducted of Members of Parliament (MPs) in the UK Parliament to assess their mental health, and to assess their awareness of the available support and treatment services.

Several factors in the UK political system may adversely influence MPs and their mental health: The UK Parliament permits MPs to hold employment outside Parliament in addition to their roles as elected representatives. Further, in the UK parliament, "whips" are appointed officials in each political party who are charged with organising their party's parliamentary business and ensuring party discipline amongst MPs. In addition, a confidential in-house service is provided within Parliament for MPs and peers, called the Parliamentary Health and Wellbeing Service, to support their occupational health and wellbeing.

For peer review only - http://bmjopen.bmj.com/site/about/guidelines.xhtml

In this context, the UK Parliamentary Mental Health (UKPMH) study aims are to: (i) assess the overall mental health of MPs by drawing comparisons with a nationally representative survey in England, and with comparator socio-demographic and occupational groups within the survey; and (ii) assess awareness among MPs of the mental health support services available to them.

The principal research question was: What is the prevalence of common mental disorders among MPs? The secondary questions addressed were: how far are MPs aware of mental health services that can assist them with mental health problems? Are MPs willing to discuss their mental health with party whips or other MPs? This study tested the following primary hypothesis: the occurrence of common mental disorders (CMDs) is higher among MPs compared to the general population and compared with specific socio-demographic, professional and occupational comparator groups.

METHODS

Study design and participants

We conducted an anonymised, online self-completed survey at the House of Commons in December 2016. The inclusion criteria for participation were: membership of the 56th UK Parliament, House of Commons; and providing written, informed consent. We followed the STROBE guidelines for observational studies for the reporting of this cross-sectional study.[19] No age limits were defined, except that to be elected to Parliament one must be over 18 years old. Participants were sent via email an invitation letter to participate. Initially, in November 2016 a letter was sent to all 650 members of the House of Commons to make them aware of the study. In early December, a letter including a web link to an online survey with an individual access code was sent out via to all MPs internal post, and via email. The survey took place between 5 and 31 December 2016. Repeated efforts were taken to promote participation and maximise response rates in the survey. The study information sheet (explaining the purpose of the study) and instructions for the online questionnaire, as well as two

For peer review only - http://bmjopen.bmj.com/site/about/guidelines.xhtml

reminder emails, were sent out with clear descriptions of encrypted data collection and protection measures to ensure anonymity.

Ethics and data protection

At all times throughout the study preparation, conduct and analysis, particular consideration and care has been given to the specific, sensitive study context, and to the potential vulnerability of participants, namely the risk of sensationalised coverage should any individual be identifiable. Ethics approval for the study was obtained in September 2016 from King's College London Ethics Committee (reference number: HR-16/17-3118). Efforts were taken to limit distress and secure confidentiality for the participants. To ensure full confidentiality no personal identifiers were collected, and identifiers were removed if provided. All participants were provided with contact information for the Parliamentary Health and Wellbeing Service in the introductory letter and via the online survey in case any participants were experiencing distress at the time of the survey.

Health Survey for England comparator groups

Data for the comparator groups were elicited from the Health Survey for England (HSE) 2014. The HSE is an annual survey which uses a multi-stage stratified design to sample nationally representative random cross section of the population of England each year. Participants are visited by an interviewer who collects demographic and socio-economic data, and information on health and health-related behaviours. A detailed description of the HSE has been reported elsewhere.[20] From the HSE, we identified four comparison groups: total population of England in the HSE England population (EN), corporate managers in England (CM), all managers in England (AM), and those in high-income groups in England (HIG). The socio-economic groups derive from a standardised questionnaire asked in the HSE to all survey respondents.

Measures of mental health

For peer review only

143 The General Health Questionnaire (GHQ-12) was used to assess the mental health of respondents in the UKPMH sample and the HSE 2014. The self-completed 12-item GHQ-12 is one of the most extensively used screening instruments for common mental disorders, measured by a 4-point Likert scale (ranging from 'less than usual' to 'much more than usual') across twelve items.[20, 21]

147 Scoring of the GHQ-12 for the present study was done in the original bi-modal method as developed by Goldberg.[22] Specifically, each symptom was scored either 0 if 'not at all present' or present 'no more than usual', or 1 for symptoms that were present 'rather more than usual' or 'much more than usual'). The scoring method allowed for total scores to range from 0 to 12. No formal threshold exists for identifying probable mental ill health, with optimal values likely to be specific to the population under study. However, in line with the previous HSE survey, MP's total scores are grouped according to three categories: 0 (indicating no evidence of probable mental ill health), 1 to 3 (indicating less than optimal mental health), and 4 or more (indicating probable psychological disturbance or mental ill health).[20, 21]

156 The GHQ-12 has been extensively validated across international settings for screening and detection of the common mental disorders.[23] In previous work, with a cut-off point ≧4, the total score of the GHQ-12 was found in a UK setting to have a sensitivity of 84.6% and specificity of 89.3% when assessed against *International Classification of Mental Disorders (ICD-10) and the Diagnostic Statistical Manuals-IV (DSM-IV),* diagnoses derived from the Composite International Diagnostic Interview (CIDI-PC) for the common mental disorders (including depression, dysthymia, generalised anxiety disorder, panic disorder and other related conditions).[23]

163 A technical error in the administration of the questionnaire caused a lack of indication for respondents of the 4th option (much more/much less than usual) on GHQ-12 items 8, 9, 10, 11, 12. However, this has no impact on the total scores of GHQ-12 for each participant, as the third and fourth option are grouped together in the bi-modal scoring.

For peer review only - http://bmjopen.bmj.com/site/about/guidelines.xhtml

In the question on awareness of the Parliamentary Health and Wellbeing Service, a technical error in the administration of the questionnaire caused 4 options (no/ unsure/ unaware/ yes) to be offered rather than binary yes and no options. The three options (no/ unsure/ unaware) were combined to represent "no awareness".

Covariates

Core demographic questions were obtained from the UKPMH study sample: Age (categorised into five groups: 21 to 30; 31 to 40; 41 to 50; 51 to 60; 61 to 70, >70 years), sex (female or male), and educational status (GCSE/ O level, A Level, Vocational Qualifications, Undergraduate Degree, Post Graduate Degree, Doctorate), as well as years serving as MP. MPs were also asked if they were aware of the mental health services available to them, as well as their willingness to discuss their mental health with their Whips and other MPs (full list of questions in Supplementary File). Ethnicity was not assessed. Due to the low number of MPs from a minority ethnic background in the 56th House of Commons (n=41), this avoided any concern about the identification of participants, which may have further limited the response rate.

Statistical analyses

All statistical analyses were performed using STATA 14.1. Within the UKPMH sample, descriptive analyses was undertaken first to determine the distribution of each item of the GHQ-12 and of socio-demographic characteristics, awareness of mental health services, and willingness to discuss mental health issues with party whips or with fellow MPs.

The UKPMH sample is subject to "unit non-response" as 22.4% of all MPs completed the survey. To address this issue, we employed inverse probability weighting (IPW)[24] in the analysis, where weights are used to rebalance the set of complete cases within the MP sample to make it representative of the whole English population; we used the weighted sample of the HSE 2014. Age-sex standardised proportion estimates were calculated i) for each item of the GHQ-12, and ii) for the presence of

For peer review only - http://bmjopen.bmj.com/site/about/guidelines.xhtml

probable mental ill health. We compared i) each item of the GHQ-12, and ii) the three combined categories derived from the total score of the GHQ-12 that indicate the presence of probable mental ill health of the MP sample with a range of socio-demographic groups (the English population (EN), corporate managers (CM), all managers (AM), and with high income groups (HIG) in England) derived from HSE 2014. As a sensitivity analyses, age-sex standardised proportion estimates were calculated separately for males and females.

Non-parametric tests (chi-square) and parametric tests (t-test for unequal sample sizes) were employed to explore potential differences in the proportion estimates between UKPMH and HSE 2014 samples.

Cross-sectional associations of whether an MP had additional employment outside Parliament with each different item of the GHQ-12, and with the three combined categories (indicating no evidence of probable mental ill health, less than optimal mental health, probable psychological disturbance or mental ill health) were explored with the use of ordinal logistic regression models. Results were expressed as increased risk (odds ratio and corresponding 95% confidence intervals) of being in a highest category of each item of the GHQ-12 for those MPs with a work role outside parliament were compared to those without such an external role.

In addition, linear regression models were employed to explore the mean difference in the GHQ-12 total scores for those MPs who had additional employment outside Parliament, and for those who did not. All models were adjusted for the following potential confounders identified a priori: age, sex and educational status. Age-sex standardised inverse probability weights were employed for all linear and ordinal regression models.

Patient and Public Involvement

Daniel Poulter, MP, was involved at all stages of the study and is co-author of the paper. Other parliamentarians and staff of the Parliamentary Health and Wellbeing Service were consulted at the

For peer review only - http://bmjopen.bmj.com/site/about/guidelines.xhtml

planning and design stages, as well as at the interpretations of the findings and dissemination stages of the study.

RESULTS

Questionnaires were returned by 146 respondents (22.4%) of the 650 MPs. Median time to complete the survey was 4 minutes (IQR: 3 to 5). Most respondents were male (63%), with an undergraduate (44%) or a postgraduate degree (36%) or doctorate (2%). Most were between 41 and 60 years old (66%), and most did not work outside parliament (81%) (see Table 1).

Table 1: Demographic characteristics of UKPMH participants

	MP sample (N=146)	Total Health Survey for England sample (N=7871)
	n (%)	n (%)
Below 40 years old	27 (18%)	4014 (51%)
Female	54 (36%)	4385 (55%)
Higher education degree	119 (82%)	888 (11.3%)
Knowledge on how to access to mental health support	65 (45%)	n/a
Unaware of parliamentary well-being service	112 (77%)	n/a
Willing to discuss mental health problems with whips	70 (48%)	n/a
Willing to discuss mental health problems with other MPs	76 (52%)	n/a
Presence of CMD (according to ≥4 cut point on the GHQ-12 total score)	49 (34%)	2902 (26%)

Mental health of MPs and the HSE 2014 comparator groups

Table 2 presents weighted proportion estimates and corresponding 95% confidence intervals of the UKPMH sample and the four different predetermined HSE 2014 occupational and sociodemographic comparator groups (EN, CM, AM, HIG). For each item of the GHQ-12, the UKPMH sample presented a higher weighted proportion of participants who had lower levels of concentration, were losing sleep because of worry, were feeling less useful, were less capable of making decisions, and were feeling under constant strain, compared to the four HSE 2014 occupational and sociodemographic comparison groups (p-values of chi-square test <0.001).

For peer review only

In addition, a higher weighted proportion of MPs could not overcome difficulties, were less able to enjoy normal day to day activities, were less able to face up to their problems, reported losing confidence in themselves, or feeling unhappy and depressed, and more individual MPs considered themselves to be a worthless person (p-values of chi-square test <0.001). Compared to the HSE 2014 predetermined occupational and sociodemographic comparator groups, a higher weighted proportion of the MPs also reported being less able to feel reasonably happy (p-values of chi-square test <0.001).

When we compared the weighted proportions of the three combined categories derived for the GHQ-12 total score that indicate the presence of probable mental ill health between the UKPMH and HSE 2014 samples, we found that a higher proportion of MPs had probable mental ill health (weighted proportion: 34%; 95% CI: 27%, 42%), compared with EN (weighted proportion: 26%; 95% CI: 25%, 27%), CM (weighted proportion: 22%; 95% CI: 18%, 26%), AM (weighted proportion: 23%; 95% CI: 20%, 27%) and HIG (weighted proportion: 17%; 95% CI: 13% to 21%) (p-values of chi-square test <0.001) (see Table 2 and Figure 1). In addition, female MPs had higher rates of probable mental ill health (weighted proportion: 41%; 95% CI: 27%, 56%) compared to male MPs (weighted proportion: 30%; 95% CI: 21%, 41%) (see Supplementary File, Table S1 and Table S2).

Table 2: Descriptive characteristics of the 12 item GHQ (GHQ-12), and the four different predetermined HSE 2014 occupational and sociodemographic comparator groups (EN, CM, AM, HIG).

	n	WP<br>95%CI	n	WP<br>95% CI	n	WP<br>95% CI	n	WP<br>95% CI	n	WP<br>95% CI
		MP		EN		CM		AM		HIG
**Item 1: Have you recently been able to concentrate on whatever you're doing?**										
Better than usual	5	0.03<br>0.01 to 0.07	223	0.035<br>0.03 to 0.04	15	0.03<br>0.02 to 0.05	24	0.03<br>0.02 to 0.05	10	0.03<br>0.01 to 0.05
Same as usual	93	0.66<br>0.57 to 0.74	6073	0.85<br>0.84 to 0.86	394	0.88<br>0.84 to 0.91	602	0.88<br>0.85 to 0.91	371	0.9<br>0.87 to 0.93
Less than usual	40	0.26<br>0.19 to 0.34	771	0.1<br>0.10 to 0.11	38	0.08<br>0.06 to 0.11	53	0.08<br>0.06 to 0.10	29	0.07<br>0.05 to 0.10
Much less than usual	8	0.05<br>0.02 to 0.11	103	0.01<br>0.01 to 0.02	2	0.01<br>0.00 to 0.04	3	0.01<br>0.00 to 0.03	1	0.005<br>0.00 to 0.01
**Item 2: Have you recently lost much sleep over worry?**										
Not at all	24	0.18<br>0.12 to 0.26	2334	0.33<br>0.32 to 0.34	146	0.33<br>0.28 to 0.38	226	0.33<br>0.29 to 0.37	130	0.3<br>0.26 to 0.35
No more than usual	66	0.47<br>0.38 to 0.56	3573	0.5<br>0.49 to 0.51	246	0.54<br>0.49 to 0.59	370	0.55<br>0.50 to 0.59	220	0.56<br>0.51 to 0.61
Rather more than usual	38	0.26<br>0.19 to 0.34	1035	0.14<br>0.13 to 0.15	51	0.11<br>0.08 to 0.14	76	0.11<br>0.09 to 0.14	55	0.13<br>0.10 to 0.16

For peer review only - http://bmjopen.bmj.com/site/about/guidelines.xhtml

For peer review only

Much more than usual	18	0.1<br>0.06 to 0.16	240	0.03<br>0.02 to 0.04	7	0.02<br>0.01 to 0.03	11	0.02<br>0.01 to 0.03	6	0.01<br>0.00 to 0.03
**Item 3: Have you recently felt you were playing a useful part in things?**										
More so than usual	27	0.19<br>0.13 to 0.27	676	0.10<br>0.09 to 0.11	58	0.16<br>0.12 to 0.21	83	0.14<br>0.11 to 0.18	39	0.10<br>0.07 to 0.13
Same as usual	67	0.46<br>0.38 to 0.55	5696	0.8<br>0.79 to 0.81	362	0.77<br>0.72 to 0.81	548	0.78<br>0.74 to 0.81	339	0.82<br>0.77 to 0.85
Less useful than usual	43	0.3<br>0.22 to 0.39	625	0.08<br>0.07 to 0.09	26	0.07<br>0.05 to 0.10	47	0.08<br>0.06 to 0.10	30	0.08<br>0.05 to 0.12
Much less useful	9	0.05<br>0.02 to 0.11	157	0.02<br>0.01 to 0.03	3	0.005<br>0.00 to 0.02	4	0.005<br>0.00 to 0.02	3	0.01<br>0.00 to 0.02
**Item 4: Have you recently felt capable of making decisions about things?**										
More so than usual	9	0.06<br>0.03 to 0.11	509	0.08<br>0.07 to 0.09	29	0.07<br>0.05 to 0.11	42	0.07<br>0.05 to 0.09	28	0.07<br>0.05 to 0.10
Same as usual	118	0.84<br>0.77 to 0.89	6162	0.85<br>0.84 to 0.86	403	0.88<br>0.84 to 0.91	613	0.89<br>0.86 to 0.91	367	0.89<br>0.85 to 0.92
Less so than usual	17	0.09<br>0.05 to 0.15	444	0.066<br>0.06 to 0.08	17	0.04<br>0.02 to 0.07	27	0.04<br>0.03 to 0.06	16	0.04<br>0.02 to 0.07
Much less capable	2	0.01<br>0.00 to 0.05	66	0.01<br>0.01 to 0.01	1	0<br>0.00 to 0.02	1	0<br>0.00 to 0.01	0	NA
**Item 5: Have you felt under constant strain recently?**										
Not at all	9	0.07<br>0.03 to 0.13	1778	0.25<br>0.24 to 0.27	130	0.28<br>0.24 to 0.33	194	0.28<br>0.24 to 0.31	94	0.22<br>0.18 to 0.27
No more than usual	60	0.41<br>0.33 to 0.50	3974	0.56<br>0.54 to 0.57	243	0.54<br>0.49 to 0.59	374	0.55<br>0.51 to 0.59	236	0.57<br>0.51 to 0.62
Rather more than usual	53	0.38<br>0.30 to 0.47	1192	0.16<br>0.15 to 0.17	69	0.17<br>0.13 to 0.21	102	0.16<br>0.13 to 0.20	75	0.19<br>0.15 to 0.24
Much more than usual	24	0.14<br>0.09 to 0.21	225	0.03<br>0.02 to 0.03	7	0.02<br>0.01 to 0.03	12	0.02<br>0.01 to 0.03	6	0.02<br>0.01 to 0.04
**Item 6: Have you recently felt you couldn't overcome your difficulties?**										
Not at all	41	0.29<br>0.21 to 0.37	2659	0.38<br>0.37 to 0.39	183	0.4<br>0.35 to 0.45	278	0.4<br>0.36 to 0.44	156	0.36<br>0.31 to 0.41
No more than usual	76	0.52<br>0.44 to 0.61	3762	0.52<br>0.51 to 0.53	234	0.53<br>0.47 to 0.58	352	0.52<br>0.48 to 0.56	229	0.57<br>0.52 to 0.62
Rather more than usual	24	0.16<br>0.10 to 0.23	602	0.08<br>0.08 to 0.09	31	0.07<br>0.05 to 0.10	48	0.07<br>0.05 to 0.09	23	0.06<br>0.04 to 0.09
Much more than usual	5	0.03<br>0.01 to 0.08	143	0.02<br>0.02 to 0.02	2	0.01<br>0.00 to 0.03	5	0.01<br>0.00 to 0.02	2	0<br>0.00 to 0.02
**Item 7: Have you recently been able to enjoy your normal day to day activities?**										
More so than usual	6	0.03<br>0.01 to 0.06	376	0.06<br>0.05 to 0.07	35	0.11<br>0.07 to 0.16	47	0.09<br>0.06 to 0.13	23	0.05<br>0.04 to 0.08
Same as usual	88	0.61<br>0.52 to 0.69	5649	0.79<br>0.78 to 0.80	358	0.76<br>0.71 to 0.81	544	0.77<br>0.73 to 0.81	344	0.83<br>0.79 to 0.87
Less so than usual	36	0.27<br>0.19 to 0.36	924	0.12<br>0.12 to 0.13	47	0.11<br>0.08 to 0.14	78	0.12<br>0.09 to 0.15	40	0.11<br>0.08 to 0.15
Much less than usual	16	0.10<br>0.06 to 0.16	225	0.025<br>0.02 to 0.03	9	0.02<br>0.01 to 0.04	14	0.02<br>0.01 to 0.03	4	0.01<br>0.00 to 0.02
**Item 8: Have you recently been able to face up to your problems?**										
More so than usual	9	0.07<br>0.04 to 0.13	340	0.06<br>0.05 to 0.07	19	0.06<br>0.04 to 0.11	30	0.06<br>0.04 to 0.09	17	0.05<br>0.03 to 0.08
Same as usual	118	0.80<br>0.71 to 0.86	6157	0.87<br>0.86 to 0.88	404	0.90<br>0.85 to 0.93	610	0.9<br>0.86 to 0.92	372	0.91<br>0.87 to 0.94
Less able than usual	19	0.14<br>0.08 to 0.21	510	0.07<br>0.06 to 0.07	15	0.03<br>0.02 to 0.06	27	0.04<br>0.03 to 0.06	17	0.04<br>0.02 to 0.07
Much less able	NA	NA	72	0.01<br>0.01 to 0.01	1	0.01<br>0.00 to 0.03	1	0.01<br>0.00 to 0.02	1	0.01<br>0.00 to 0.02

For peer review only

**Item 9: Have you recently been feeling unhappy and depressed?**										
Not at all	43	0.3<br>0.22 to 0.38	2846	0.4<br>0.39 to 0.42	213	0.47<br>0.42 to 0.52	318	0.47<br>0.43 to 0.51	168	0.39<br>0.34 to 0.44
No more than usual	59	0.42<br>0.33 to 0.51	3119	0.44<br>0.43 to 0.45	178	0.42<br>0.37 to 0.47	271	0.41<br>0.37 to 0.46	202	0.52<br>0.47 to 0.58
Rather more than usual	44	0.29<br>0.21 to 0.37	911	0.13<br>0.12 to 0.15	44	0.1<br>0.08 to 0.14	70	0.11<br>0.08 to 0.13	34	0.08<br>0.06 to 0.11
Much more than usual	NA	NA	206	0.03<br>0.01 to 0.04	3	0.01<br>0.00 to 0.02	7	0.01<br>0.01 to 0.03	3	0.01<br>0.00 to 0.02
**Item 10: Have you recently been losing confidence in yourself?**										
Not at all	53	0.37<br>0.29 to 0.46	3192	0.45<br>0.44 to 0.47	232	0.52<br>0.47 to 0.58	349	0.52<br>0.48 to 0.56	201	0.47<br>0.42 to 0.53
No more than usual	65	0.45<br>0.36 to 0.54	2979	0.42<br>0.41 to 0.43	175	0.4<br>0.35 to 0.45	261	0.39<br>0.35 to 0.43	174	0.44<br>0.39 to 0.50
Rather more than usual	28	0.18<br>0.13 to 0.26	739	0.1<br>0.10 to 0.11	24	0.06<br>0.04 to 0.10	46	0.08<br>0.06 to 0.10	32	0.08<br>0.06 to 0.12
Much more than usual	NA	NA	170	0.02<br>0.02 to 0.03	5	0.01<br>0.00 to 0.02	9	0.015<br>0.01 to 0.02	NA	NA
**Item 11: Have you recently been thinking of yourself as a worthless person?**										
Not at all	86	0.58<br>0.49 to 0.66	4689	0.66<br>0.65 to 0.68	323	0.73<br>0.68 to 0.77	480	0.72<br>0.68 to 0.75	285	0.69<br>0.64 to 0.74
No more than usual	44	0.31<br>0.24 to 0.40	1879	0.26<br>0.25 to 0.27	95	0.22<br>0.18 to 0.26	154	0.23<br>0.20 to 0.27	107	0.27<br>0.23 to 0.32
Rather more than usual	16	0.11<br>0.06 to 0.18	378	0.05<br>0.05 to 0.06	16	0.05<br>0.03 to 0.08	26	0.05<br>0.03 to 0.07	13	0.03<br>0.02 to 0.06
Much more than usual	NA	N NA<br>A	133	0.02<br>0.02 to 0.02	3	0.01<br>0.00 to 0.02	6	0.01<br>0.00 to 0.02	2	0.01<br>0.00 to 0.02
**Item 12: Have you recently been feeling reasonably happy, all things considered?**										
More so than usual	16	0.09<br>0.05 to 0.15	698	0.11<br>0.10 to 0.11	45	0.13<br>0.09 to 0.18	66	0.12<br>0.09 to 0.15	39	0.11<br>0.08 to 0.14
About same as usual	96	0.67<br>0.59 to 0.75	5633	0.79<br>0.78 to 0.80	364	0.8<br>0.75 to 0.85	553	0.81<br>0.77 to 0.84	346	0.84<br>0.80 to 0.88
Less so than usual	34	0.24<br>0.17 to 0.32	611	0.08<br>0.08 to 0.09	25	0.05<br>0.04 to 0.08	42	0.06<br>0.04 to 0.08	20	0.05<br>0.03 to 0.08
Much less than usual	NA	NA	137	0.02<br>0.02 to 0.02	4	0.01<br>0.00 to 0.03	7	0.01<br>0.01 to 0.03	2	0<br>0.00 to 0.02
**Presence of probable mental ill health**										
No evidence of probable mental ill health	35	0.25<br>0.18 to 0.34	4256	0.53<br>0.52 to 0.55	290	0.58<br>0.53 to 0.62	446	0.58<br>0.54 to 0.62	254	0.56<br>0.51 to 0.61
Less than optimal mental ill health	62	0.40<br>0.32 to 0.49	1620	0.2<br>0.19 to 0.21	97	0.2<br>0.17 to 0.25	140	0.19<br>0.16 to 0.22	117	0.27<br>0.23 to 0.32
Probable mental ill health	49	0.34<br>0.27 to 0.43	2141	0.26<br>0.25 to 0.27	108	0.22<br>0.18 to 0.26	170	0.23<br>0.20 to 0.27	74	0.17<br>0.13 to 0.21

Weighted proportion (WP) with the corresponding 95% Confidence Intervals (CI).

Key: MP: Member of Parliament Sample; EN: English Population (HSE 2014); CM: Corporate Managers (HSE 2014); AM: All managers (HSE 2014); HIG: high-income group (HSE 2014).

For peer review only - http://bmjopen.bmj.com/site/about/guidelines.xhtml

Characteristics of respondents in comparison to all MPs

Compared with all 650 MPs, those who participated were younger (18 %, n=27 vs. 16% of total MP population were below 40 years old), more likely to be female (37%, n=54 of the UKPMH sample vs 30% of total MPs population were female) in relation to the gender distribution of the total number of MPs, and more educated (81%, n=119) of the UKPMH sample had a university degree vs. 76% of total MP population.

Awareness of mental health support services

Most MPs were not aware of the mental health services provided by the Parliamentary Health and Wellbeing Service within parliament. Most MPs (55 %) did not know how to access any mental health support at Parliament (see Figure 2). When asked whether they felt the Parliamentary Health and Wellbeing Service currently offered sufficient support, a large majority of MPs (77%) were unaware of what options are currently offered by the service and only 23% were aware that support was sufficiently available (see Figure 3).

(Figures 2, 3, 4, 5 about here)

Willingness to disclose poor mental health

Most MPs who took part in this survey were not willing to discuss mental health problems with their party whips (52%), and only a small majority of MPs would feel able to talk with other MPs about their mental health (52%) (see Figures 4 and 5). After adjusting for age, sex and educational status, we found evidence that MPs who were willing to discuss their mental health with their party whips or fellow MPs, had a reduced risk of CMDs (willing to discuss with whips: adjusted OR: 0.32; (95% CI: 0.16, 0.31), or discuss with fellow MPs: adjusted OR: 0.57; (95% CI: 0.30, 0.99) .

Additional employment outside parliament

For peer review only - http://bmjopen.bmj.com/site/about/guidelines.xhtml

We found no evidence of an association between having additional employment outside Parliament with the individual GHQ-12 items, or an increased total GHQ score indicating poor mental health (see Supplementary File, Table S3).

DISCUSSION

Principal findings

The main findings of this study were: (1) strong evidence to indicate that a higher proportion of MPs had poor mental health than among the general population, than among the defined occupational and socio-demographic comparator groups (EN, CM, AM, HIG). The primary study hypothesis was therefore confirmed. (2) Most MPs were not aware of Parliamentary mental health and support services. (3) Most MPs were not willing to discuss their mental health with party whips, and only a small majority would be happy to discuss mental health issues with other MPs. (4) Having employment outside Parliament, in addition to the role of MP, is not linked with increased risk for mental ill health.

The Parliamentary Health and Wellbeing Service is the occupational health service provided since 2013 inside the House of Commons. It aims to support all staff and MPs in developing a healthy and safe working environment, and encourages MPs to adopt better attitudes and behaviour towards their own physical health and mental health.[25] Despite the service being in place for almost four years, the Parliamentary Health and Wellbeing Service had reported low numbers of MPs requesting support. This study confirms this reluctance to seek help in finding that a majority of MPs are unaware of the service or how to access it. Reasons for this might be insufficient advertising of the support options offered and location of the services, as well as anticipated stigma and discrimination among MPs.[26]

Strengths and weaknesses of the study

For peer review only

301 The study has several limitations and potential biases. First, the response rate was relatively low (22.4%). Given the intense work loads of MPs, this may have been partly due to the additional work load of completing the survey, even though the median time to complete survey was only 4 minutes. Notably, a possible fear of being identified, of stigmatisation, and of the potential reputational damage associated with adverse media coverage may have influenced the response rate. We tried to reduce these biases by promoting the survey in Parliament, by sending several reminders, and by stressing the brevity, as well as the anonymity of the survey. Generally, MPs are a difficult survey population to engage, which has also been confirmed in a 2008 internal UK Parliament survey, where only 14.5% (94 MPs) responded.[27]

310 Secondly, it is also possible that MPs who responded to the online survey may have increased stress or mental ill health and that therefore a greater number of them were willing to complete the survey. A potential self-selection bias may therefore be present in the UKPMH sample. However, there is also a potential risk of under-reporting from people who might be reluctant to take part in the study, because they are affected by mental health problems, or because of the stigma associated with the topic.

316 Respondents tended to be younger in relation to the age distribution of all MPs (18% of the UKPMH sample vs. 16% of total MP population were below 40 years old), and more likely to be female (36% female of the UKPMH sample vs 30% of total MPs population were female) in relation to the gender distribution of the total number of MPs and had a university degree (81% of the UKPMH sample vs 76% of total MP population). We did not assess marital or cohabitation status, as this would have increased the risk of identifiability of MPs, and this may have therefore also adversely affected the response rate.

323 Thirdly, comparing MPs to other occupational and socio-demographic groups within a population presents challenges. We considered comparing the UKPMH sample to the UK Health and Safety Executive's Labour Force Survey (LFS), which provides annual data on rates of mental disorder by

For peer review only - http://bmjopen.bmj.com/site/about/guidelines.xhtml

326 occupation.[28] However, the LFS relies on random household sampling is poorly suited to
327 extrapolating meaningful data for a relatively small group 650 UK MPs. Published LFS data lacks
328 sufficient granularity to be able to analyse the prevalence of mental disorders at an occupation-
329 specific level, which for politicians would be 'elected officers and representatives'.[29] Given the
330 unique features of political careers, including the diverse backgrounds from which politicians may be
331 drawn, specific data relating to these generic occupational groupings are unlikely to be fully helpful in
332 understanding why there is a higher burden of mental ill health. In this sample we found that having
333 employment outside Parliament, and in addition to the role of MP, does not seem to constitute an
334 increased risk for mental ill health. However, we regard this outcome with caution as this study may
335 be underpowered to test for this specific variable, as most participants (81%) did not have
336 employment outside Parliament.

337 **Comparison of results with earlier studies**

338 When examining UK parliamentary working hours reform, research found high levels of physical and
339 emotional stress as a result of various aspects of political life such as additional work roles, extensive
340 travel and job insecurity.[30] A longitudinal study in new UK MPs highlighted increased levels of stress
341 post-election.[31] In 2008 the UK Parliament also conducted its own informal survey regarding
342 experience and perceptions of mental illness, which concluded that one in five MPs had a personal
343 experience of a mental health problem, and one in three felt stigma was a barrier to openness about
344 mental health, yet no data on CMD were collected.[27] Given that work characteristics promoting
345 stress are associated with mental disorders,[32, 33] it may be reasonable to assume that rates of CMD
346 would be high in parliamentarians. However, no rigorous assessment has previously been conducted
347 to investigate this issue.

348 Selected studies have investigated mental health in politicians, and although they have drawn on
349 biographical evidence, their findings are in line with the results of this study. One study rated 46
350 statesmen and national leaders' biographies for psychopathology, and found increased rates for

For peer review only

lifetime psychopathology, episodes of mental ill health, with only 15.2% of politicians showing no psychopathology at all.[34] A review of biographical sources looking at mental disorders in U.S. Presidents between 1776 and 1974, found that eighteen (49%) presidents met criteria indicative of psychiatric disorders.[35]

This is the first study of assessment of mental health in members of Parliament of the UK House of Commons using structured, validated scales. These findings indicate that MPs are more likely to experience probable mental ill health and symptoms indicative of mental distress compared to the general population, and compared with similar occupational and professional groups. In addition, most MPs are not aware of mental health support offered by the Parliamentary Health and Wellbeing service, or willing to disclose to their whips or other MPs. This leaves MPs who have experience of mental ill health facing considerable difficulties without knowing how to access help.

Interpretation of the results

A number of studies have examined media and public reactions to politicians' actual or perceived mental health problems.[14-16] In an ever more hostile media environment, poor mental health can be regarded as a factor limiting politicians in their capacities. Stigma against people with mental disorders is prevalent in all countries and all sectors of society. It was not until 2013 that the UK passed the Mental Health (Discrimination) (No 2) Act 2013, which removed discriminatory provisions permitting Members of Parliament (MPs) with mental health problems to be disqualified under certain circumstances.[36] Subsequent to the Act, there have been more disclosures from politicians about personal mental health problems. However, given that the results of this study showed that only 48% of surveyed MPs felt able to talk to their party whips, and only about half (52%) felt able to talk to another MP about their mental health, stigma and self-stigma about mental health appears to remain a powerful barrier to seeking help and support among Members of the UK House of Commons.

For peer review only - http://bmjopen.bmj.com/site/about/guidelines.xhtml

The power of disclosure as a catalyst for overcoming stigma has been demonstrated in 1998 when Kjell Magne Bondevik, then Prime Minister of Norway, spoke publicly about his experience of depression. His disclosure was empathetically received by the media and by the public.[37]

In 2012, during a House of Commons debate on mental health, four MPs disclosed their own mental health experiences. This eventually paved the way to providing MPs with access to mental health services in Westminster. Consequently, the Parliamentary Health and Wellbeing Service was created in 2013 and operates a mental health referral service as well as providing general medical advice, support and guidance to MPs and other staff working at Parliament. The service is nurse-led and is supported by one occupational health doctor for 3 days each week. It does not offer the more comprehensive health service that is often provided by General Practice in the United Kingdom. Our findings show poor awareness amongst MPs of the Parliamentary Health and Wellbeing Service and how to access it. This may be related to the restricted times that the service operates, or that the service is not located on the main Parliamentary Estate. These findings support the need for an increased mental health support for MPs and raising awareness about the Parliamentary Health and Wellbeing Service. They also support the need to for mental health stigma and self-stigma reduction amongst MPs.

Implications for future research

This is an initial study into the mental health of MPs, and further work is needed to assess the key issues identified, and to assess trends in the mental health of MPs over time. Our findings are only a starting point, but they reveal MPs' mental health problems and the need to properly assess them. A more granular assessment of mental health problems, including rates and consequences of alcohol and substance use-related problems, as well as cognitive impairment would be needed to provide a more in-depth picture. In terms of prevention, a better understanding of the causes for mental health

For peer review only - http://bmjopen.bmj.com/site/about/guidelines.xhtml

problems and specific risk factors in MPs would be informative, and investigating effective mechanisms and strategies for prevention and increasing resilience. There is a need for better promotion of mental health support, such as the Parliamentary Health and Wellbeing Service, and for additional information and support for MPs in accessing the full range of mental health care. Due to their working routine and hours, MPs spend a majority of their working time far from the support provided by the NHS services in their own constituencies. In addition to their high-performance work life, this adds to the increased stress on MPs' mental health. It is also why strengthening the Parliamentary Health and Wellbeing Service could offer a specifically relevant support function. Research is also needed on mental health of other parliamentary staff, to identify their needs, and to evaluate their awareness of, and access to, the Parliamentary Health and Wellbeing Service and other relevant services.

CONCLUSION AND POLICY IMPLICATIONS

MPs have a vital role to play in the UK democracy: in making and scrutinising the legislation that governs the country, as well as in representing the interests of their constituents and the nation. This study has found the people in these important roles experience significantly higher levels of mental ill health when compared to the general population, and when compared to other senior executive and managerial groups. Most MPs do not feel that they have adequate mental health support, and they lack knowledge of how to access the mental health services that are available to them. Most MPs are not able to discuss their mental health problems with their whips or other MPs. These findings indicate that better support is required both to prevent mental health problems among MPs and to ensure rapid and effective care when needed, to support MPs in their vital work for the people they serve.

For peer review only

For peer review only - http://bmjopen.bmj.com/site/about/guidelines.xhtml

Acknowledgments: We would like to thank all Members of Parliament who took part in this study. In addition, we would like to thank Elaine Bryce (member of Dr Daniel Poulter's Parliamentary office), and the staff of the Parliamentary Health and Wellbeing Service for their support in this study.

Funding: This research received no specific grant from any funding agency in the public, commercial or not-for-profit sectors.

Competing interests:

NV acknowledges funding from the Economic and Social Research Council (ESRC) and National Institute for Health Research (NIHR) Collaboration for Leadership in Applied Health Research and Care South London at King's College London NHS Foundation Trust.

GT is supported by the National Institute for Health Research (NIHR) Collaboration for Leadership in Applied Health Research and Care South London at King's College London NHS Foundation Trust. The views expressed are those of the author(s) and not necessarily those of the NHS, the NIHR or the Department of Health. GT acknowledges financial support from the Department of Health via the National Institute for Health Research (NIHR) Biomedical Research Centre and Dementia Unit awarded to South London and Maudsley NHS Foundation Trust in partnership with King's College London and King's College Hospital NHS Foundation Trust. GT is supported by the European Union Seventh Framework Programme (FP7/2007-2013) Emerald project. GT also receives support from the National Institute of Mental Health of the National Institutes of Health under award number R01MH100470 (Cobalt study). GT is also supported by the UK Medical Research Council in relation the Emilia (MR/S001255/1) and Indigo Partnership (MR/R023697/1) awards.

IB is supported by the NIHR Biomedical Research Centre at South London and Maudsley NHS Foundation Trust and by the NIHR Collaboration for Leadership in Applied Health Research.

JD has a Clinician Scientist Fellowship, funded by the Health Foundation working with the Academy of Medical Sciences

For peer review only

All authors have completed the ICMJE uniform disclosure form at www.icmje.org/coi_disclosure.pdf and declare: no support from any organisation for the submitted work; no financial relationships with any organisations that might have an interest in the submitted work in the previous three years; DP is currently MP of the 57th UK Parliament and was member of the 56th UK Parliament; no other relationships or activities that could appear to have influenced the submitted work.

Copyright/license for publication: The Corresponding Author has the right to grant on behalf of all authors and does grant on behalf of all authors, a worldwide licence to the Publishers and its licensees in perpetuity, in all forms, formats and media (whether known now or created in the future), to i) publish, reproduce, distribute, display and store the Contribution, ii) translate the Contribution into other languages, create adaptations, reprints, include within collections and create summaries, extracts and/or, abstracts of the Contribution, iii) create any other derivative work(s) based on the Contribution, iv) to exploit all subsidiary rights in the Contribution, v) the inclusion of electronic links from the Contribution to third party material where-ever it may be located; and, vi) licence any third party to do any or all of the above."

Ethical approval: Ethics approval for the study was obtained in September 2016 from King's College London Ethics Committee (reference number: HR-16/17-3118).

Data sharing: No additional data available. The Health Survey for England 2014 can be accessed at: https://digital.nhs.uk/data-and-information/publications/statistical/health-survey-for-england/health-survey-for-england-2014. Due to the sensibility of the data, and in order to ensure full anonymity, confidentiality and data protection for the participants, the full survey data cannot be made accessible to the public.

For peer review only

For peer review only - http://bmjopen.bmj.com/site/about/guidelines.xhtml

Transparency declaration: The corresponding author affirms that the manuscript is an honest, accurate, and transparent account of the study being reported; that no important aspects of the study have been omitted; and that any discrepancies from the study as planned have been explained.

Contributors: DP and GT conceived the original idea for the study, which was then discussed with NV. NV coordinated the study. All authors contributed to the design of the study. NV and FD conducted the literature review. DP and NV collected the data. IB conducted design and analysis of the data. JD supported the design of the data analysis, and contributed throughout the design and writing up of the study. NV led the writing of the manuscript, and all authors contributed and critically revised it. All authors have given their approval for the publication of this manuscript and agree to be accountable for all aspects of the work to ensure that the questions related to the accuracy or integrity of any part of the work are appropriately investigated and resolved.

For peer review only

References

1. Davidson J. Downing Street Blues: A History of Depression and Other Mental Afflictions in British Prime Ministers: McFarland; 2010.

2. Fieve R. The case of Dominique Straus-Kahn and Mental Disorder. *International Clinical Psychopharmacology*. 2012;28:e14-e5.

3. Freedman L. Mental states and political decisions: Commentary on... Psychiatry and politicians. *The Psychiatrist*. 2011;35(4):148-50.

4. Peters U. Daniel Paul Schreber, the illness of the Senate president. *Fortschritte der Neurologie-Psychiatrie*. 1995;63(12):469-79.

5. Owen D. In sickness and in power: illnesses in heads of government during the last 100 years,. Methuen Publishing, London; 2008.

6. Owen LD. Hubris and nemesis in heads of government. *Journal of the Royal Society of Medicine*. 2006;99(11):548-51.

7. Russell G. Psychiatry and politicians: the 'hubris syndrome'. *The Psychiatrist*. 2011;35(4):140-5.

8. Sidwell B. Gaius Caligula's Mental Illness. *Classical world*. 2010;103(2):183-206.

9. Vatz RE. Rhetoric and psychiatry: A Szaszian perspective on a political case study. *Current Psychology*. 2006;25(3):173-81.

10. James DV, Mullen PE, Meloy JR, Pathé MT, Farnham FR, Preston L, et al. The role of mental disorder in attacks on European politicians 1990–2004. *Acta Psychiatrica Scandinavica*. 2007;116(5):334-44.

11. James DV, Sukhwal S, Farnham FR, Evans J, Barrie C, Taylor A, et al. Harassment and stalking of Members of the United Kingdom Parliament: associations and consequences. *The Journal of Forensic Psychiatry & Psychology*. 2016;27(3):309-30.

12. Gersons BPR, Nijdam MJ. Supporting leaders under threat and their protection. In: Parkes CM, editor. Responses to Terrorism: Can psychosocial approaches break the cycle of violence? New York: Routledge; 2014. p. 181-444.

13. Every-Palmer S, Barry-Walsh J, Pathé M. Harassment, stalking, threats and attacks targeting New Zealand politicians: A mental health issue. *Australian & New Zealand Journal of Psychiatry*. 2015;49(7):634-41.

14. Altheide DL. Mental illness and the news: The Eagleton story. *Sociology & Social Research*. 1977.

15. Dukakis MS. Campaigns and disability: When an incumbent president questions his potential successor's mental health status during the campaign. *Politics & Life Sciences*. 2015;33(2):88-92.

For peer review only

For peer review only - http://bmjopen.bmj.com/site/about/guidelines.xhtml

16. MacDonald A, Majumder RK. On the resolution and tolerance of cognitive inconsistency in another naturally occurring event: Attitudes and beliefs following the Senator Eagleton incident. *Journal of Applied Social Psychology*. 1973;3(2):132-43.

17. Tolor A. Opinions about mental illness and political ideology. *American Journal of Psychiatry*. 1973;130(11):1269-72.

18. Wykes T, Craig T. Can our politicians help to reduce stigma and discrimination? *J Ment Health*. 2013;22(3):203-6.

19. Equator Network. The Strengthening the Reporting of Observational Studies in Epidemiology (STROBE) Statement: guidelines for reporting observational studies [Available from: http://www.equator-network.org/reporting-guidelines/strobe/.

20. Craig R, Fuller E, Mindell J. Health survey for England 2014. 2015.

21. Goldberg DP, Blackwell B. Psychiatric illness in general practice: a detailed study using a new method of case identification. *Br med J*. 1970;2(5707):439-43.

22. Goldberg DP, Hillier VF. A scaled version of the General Health Questionnaire. *Psychological medicine*. 1979;9(1):139-45.

23. Goldberg DP, Gater R, Sartorius N, Ustun TB, Piccinelli M, Gureje O, et al. The validity of two versions of the GHQ in the WHO study of mental illness in general health care. *Psychol Med*. 1997;27(1):191-7.

24. Hofler M, Pfister H, Lieb R, Wittchen HU. The use of weights to account for non-response and drop-out. *Soc Psychiatry Psychiatr Epidemiol*. 2005;40(4):291-9.

25. UK Parliament. New support announced for MPs with mental health problems 12 February 2013 [Available from: http://www.parliament.uk/business/committees/committees-a-z/other-committees/members-estimate/news/new-support-announced-for-mps-with-mental-health-problems/.

26. Thornicroft G, Mehta N, Clement S, Evans-Lacko S, Doherty M, Rose D, et al. Evidence for effective interventions to reduce mental-health-related stigma and discrimination. *The Lancet*. 2016;387(10023):1123-32.

27. All-Party Parliamentary Group on Mental Health. Mental Health in Parliament: Report by the All-Party Parliamentary Group on Mental Health. 2008.

28. Health and Safety Executive. Labour Force Survey data for year 2013/14-2015/16 2016.

29. Office for National Statistics. Standard Occupational Classification 2010

30. Weinberg A, Cooper CL. Workload, stress and family life in British Members of Parliament and the psychological impact of reforms to their working hours. *Stress and Health*. 1999;15(2):79-87.

31. Weinberg A, Cooper C. Stress among national politicians elected to Parliament for the first time. *Stress and Health*. 2003;19(2):111-7.

For peer review only

32. Wilhelm K, Kovess V, Rios-Seidel C, Finch A. Work and mental health. *Social psychiatry and psychiatric epidemiology*. 2004;39(11):866-73.

33. Wang J, Lesage A, Schmitz N, Drapeau A. The relationship between work stress and mental disorders in men and women: findings from a population-based study. *Journal of Epidemiology & Community Health*. 2008;62(1):42-7.

34. Post F. Creativity and Psychopathology a Study of 291 World-Famous Men. *British Journal of Psychiatry*. 1994;165(1):22-34.

35. Davidson JR, Connor KM, Swartz M. Mental illness in U.S. Presidents between 1776 and 1974: a review of biographical sources. *J Nerv Ment Dis*. 2006;194(1):47-51.

36. Wykes T, Craig T. Can our politicians help to reduce stigma and discrimination? : Taylor & Francis; 2013.

37. Brustad S. Foreword by the Minister of Health and Care Services of Norway. In: Knapp M, McDaid D, Mossialos E, editors. Mental health policy and practice across Europe. Maidenhead: McGraw-Hill Education (UK); 2006. p. xxiv-xxv.

For peer review only

List of figures and legends

Figure 1:
Age-Sex standardised prevalence estimates and 95% Confidence Intervals of UKPMH and of specific population groups of HSE 2014 for the three different categories of Common Mental Disorders (CMD).

Key: MP: Member of Parliament Sample; EN: English Population (HSE 2014); CM: Corporate Managers (HSE 2014); AM: All managers (HSE 2014); HIG: High-income group (HSE 2014).

Figure 2:
Access to the mental health (MH) support of the Parliamentary Health and Wellbeing Service

NB: All p-values <0.001.

Figure 3:
Awareness of the mental health (MH) support of the Parliamentary Health and Wellbeing Service

NB: All p-values <0.001

Figure 4:
Willingness to talk to party whips

NB: All p-values <0.001

Figure 5:
Willingness to talk to other MPs

NB: All p-values <0.001

For peer review only

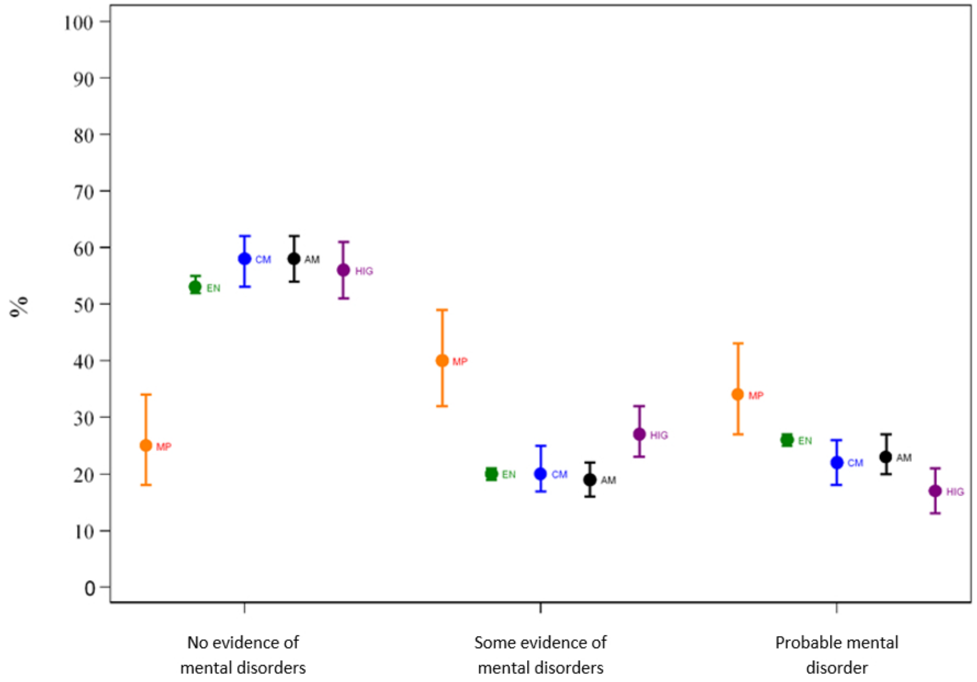

Figure 1: Age-Sex standardised prevalence estimates and 95% Confidence Intervals of UKPMH and of specific population groups of HSE 2014 for the three different categories of Common Mental Disorders (CMD).

Key: MP: Member of Parliament Sample; EN: English Population (HSE 2014); CM: Corporate Managers (HSE 2014); AM: All managers (HSE 2014); HIG: High-income group (HSE 2014).

192x141mm (120 x 120 DPI)

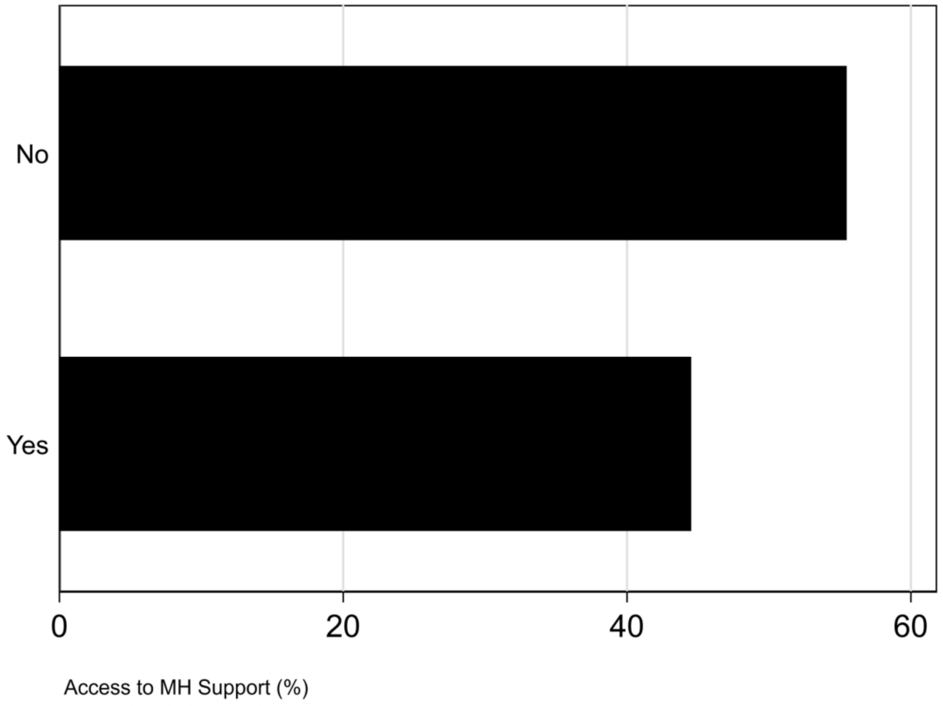

Figure 2: Access to the mental health (MH) support of the Parliamentary Health and Wellbeing Service

NB: All p-values <0.001.

169x127mm (300 x 300 DPI)

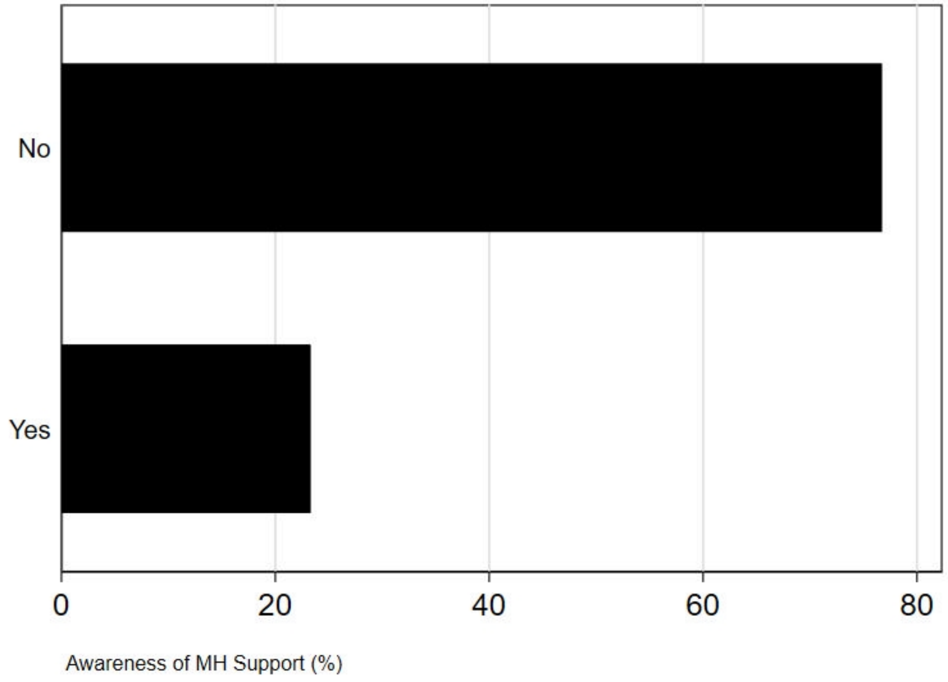

Awareness of the mental health (MH) support of the Parliamentary Health and Wellbeing Service

NB: All p-values <0.001

66x48mm (300 x 300 DPI)

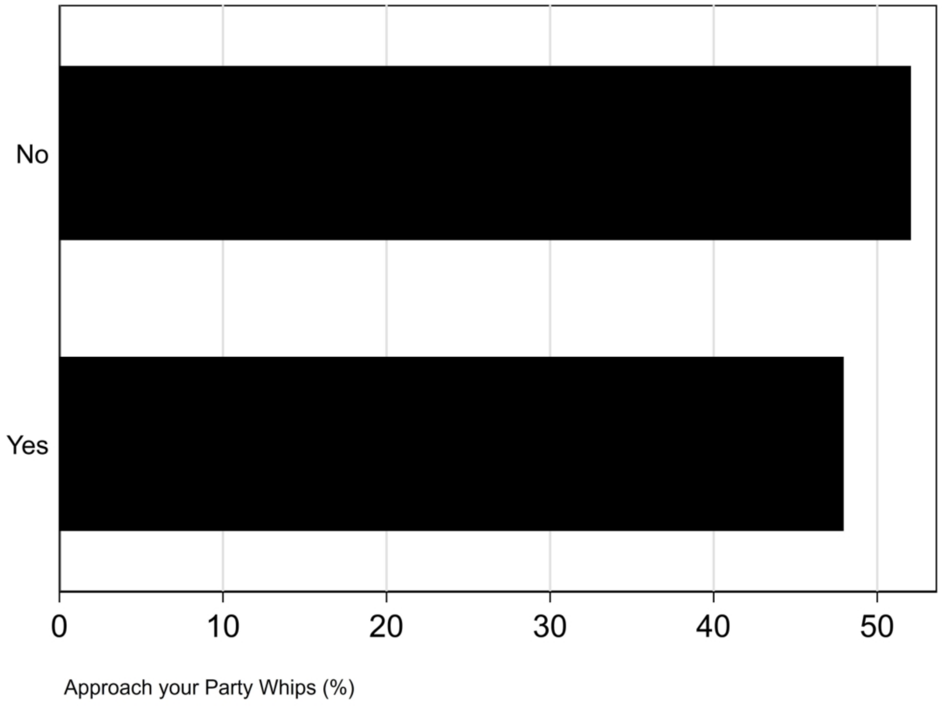

Figure 4: Willingness to talk to party whips

NB: All p-values <0.001.

169x127mm (300 x 300 DPI)

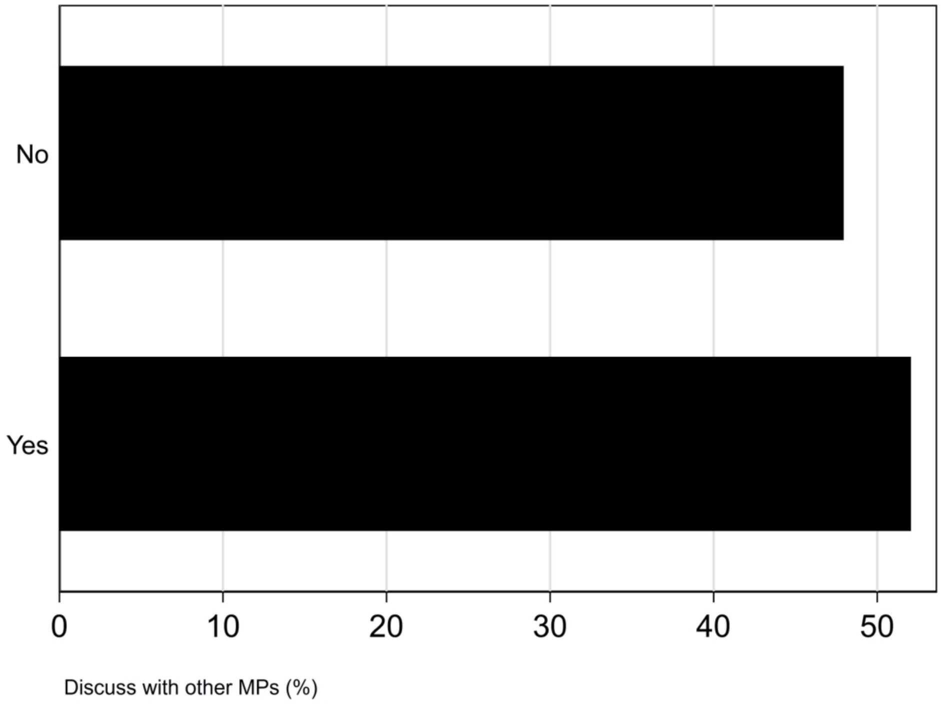

Figure 5: Willingness to talk to other MPs

NB: All p-values <0.001.

169x127mm (300 x 300 DPI)

Supplementary File (Online)

1. FULL LIST OF QUESTIONS

UKMPH Survey 2016: list of demographic questions

1. What age group are you?
    - Age 21 to 30
    - Age 31 to 40
    - Age 41 to 50
    - Age 51 to 60
    - Age 61 to 70
    - Age 70 +

2. How long have you been a Westminster MP?
    - Less than 5 years
    - 5 to 10 years
    - 11 to 15 years
    - 16 to 20 years
    - 21 to 25 years
    - More than 25 years

3. What is your highest level of educational attainment?
    - GCSE / O Level
    - A Level / Scottish Higher
    - Vocational Qualifications (BTEC, NVQ, HNC etc)
    - Undergraduate Degree (BA, BSc, or equivalent)
    - Post Graduate (MA, MSC, or equivalent)
    - Doctorate (PhD or equivalent)

4. What is your gender?
    - Male
    - Female

5. Do you have a job / role outside of Parliament?
    - Yes - Paid
    - Yes - Unpaid
    - No

For peer review only

UKMPH Survey 2016: List of questions on inhouse mental health services

6. Do you know how to access Mental Health Support through the Parliamentary Health and Wellbeing Service?
    - Yes
    - No

7. Does the Parliamentary Health and Wellbeing Service currently offer enough support to meet your mental health needs?
    - Yes
    - Somewhat
    - No

8. Would you be happy to approach your Party Whip's office if you were experiencing mental health problems?
    - Yes
    - No

9. Would you be happy to discuss with other MPs if you were experiencing mental health problems?
    - Yes
    - No

For peer review only

2. TABLE S1

Table S1: Descriptive characteristics of the 12 item GHQ (GHQ-12) and the four different predetermined HSE 2014 occupational and sociodemographic comparator groups (EN, CM, AM, HIG) - for Males

	n	WP	n	WP	n	WP	n	WP	n	WP
		MP		EN		CM		AM		HIG
		95% CI		95% CI		95% CI		95% CI		95% CI
**Age**										
21-30	2	0.00<br>0.00 to 0.01	515	0.22<br>0.20 to 0.23	26	0.19<br>0.13 to 0.27	35	0.17<br>0.12 to 0.23	32	0.18<br>0.13 to 0.25
31-40	15	0.10<br>0.06 to 0.16	558	0.17<br>0.16 to 0.19	28	0.12<br>0.08 to 0.17	46	0.14<br>0.10 to 0.18	59	0.23<br>0.18 to 0.29
41-50	27	0.31<br>0.22 to 0.42	702	0.19<br>0.18 to 0.20	56	0.22<br>0.17 to 0.28	74	0.19<br>0.16 to 0.24	57	0.19<br>0.15 to 0.25
51-60	31	0.49<br>0.38 to 0.60	606	0.16<br>0.15 to 0.17	38	0.17<br>0.13 to 0.23	66	0.20<br>0.15 to 0.24	43	0.14<br>0.10 to 0.18
61-70	15	0.10<br>0.06 to 0.17	632	0.14<br>0.13 to 0.15	51	0.17<br>0.13 to 0.22	78	0.18<br>0.14 to 0.22	45	0.13<br>0.10 to 0.18
70 +	2	0.00<br>0.00 to 0.01	565	0.12<br>0.11 to 0.13	37	0.12<br>0.09 to 0.16	60	0.13<br>0.10 to 0.16	46	0.13<br>0.09 to 0.17
**Educational attainment**										
NVQ4/NVQ5/ Degree	0	0.79<br>0.69 to 0.87	931	0.28<br>0.26 to 0.30	98	0.42<br>0.35 to 0.49	122	0.34<br>0.29 to 0.40	214	0.77<br>0.72 to 0.82
Higher ed below degree	72	0.09<br>0.04 to 0.17	524	0.13<br>0.12 to 0.14	29	0.13<br>0.09 to 0.19	46	0.13<br>0.09 to 0.17	29	0.08<br>0.06 to 0.12
NVQ3/GCE A Level	8	0.03<br>0.01 to 0.08	504	0.16<br>0.15 to 0.17	36	0.16<br>0.11 to 0.22	58	0.18<br>0.14 to 0.23	20	0.08<br>0.05 to 0.13
NVQ2/GCE O Level	3	0.10<br>0.05 to 0.19	631	0.18<br>0.17 to 0.20	41	0.17<br>0.13 to 0.23	66	0.18<br>0.14 to 0.23	13	0.04<br>0.02 to 0.07
NVQ1/CSE other grade	9	N/A	190	0.05<br>0.04 to 0.06	5	0.03<br>0.01 to 0.07	9	0.03<br>0.01 to 0.06	2	0.01<br>0.00 to 0.02
Foreign/other	0	N/A	9	0.00<br>0.00 to 0.01	0	0.09<br>0.06 to 0.14	1	0.01<br>0.00 to 0.04	0	0.01<br>0.00 to 0.03
No qualification	0	N/A	768	0.20<br>0.19 to 0.21	26	N/A	56	0.14<br>0.10 to 0.18	4	N/A
**GHQ -12**										
**Item 1: Have you recently been able to concentrate on whatever you're doing?**										
Better than usual	2	0.02<br>0.00 to 0.10	100	0.04<br>0.03 to 0.05	5	0.03<br>0.01 to 0.07	9	0.03<br>0.01 to 0.06	7	0.03<br>0.01 to 0.06
Same as usual	61	0.68<br>0.56 to 0.77	2746	0.87<br>0.85 to 0.88	193	0.89<br>0.83 to 0.94	290	0.90<br>0.85 to 0.93	239	0.92<br>0.87 to 0.95
Less than usual	26	0.26<br>0.18 to 0.37	284	0.08<br>0.07 to 0.09	12	0.06<br>0.03 to 0.11	18	0.06<br>0.04 to 0.10	14	0.05<br>0.03 to 0.09
Much less than usual	3	0.04<br>0.01 to 0.12	40	0.01<br>0.01 to 0.02	2	0.02<br>0.00 to 0.08	3	0.02<br>0.00 to 0.06	0	N/A
**Item 2: Have you recently lost much sleep over worry?**										
Not at all	18	0.19<br>0.12 to 0.30	1211	0.38<br>0.36 to 0.40	81	0.39<br>0.32 to 0.46	128	0.39<br>0.34 to 0.45	92	0.33<br>0.28 to 0.40
No more than usual	42	0.47<br>0.36 to 0.58	1519	0.48<br>0.46 to 0.50	114	0.53<br>0.45 to 0.60	166	0.52<br>0.46 to 0.58	136	0.56<br>0.49 to 0.62
Rather more than usual	26	0.27<br>0.19 to 0.38	352	0.11<br>0.10 to 0.12	15	0.07<br>0.04 to 0.12	23	0.08<br>0.05 to 0.11	30	0.11<br>0.07 to 0.15
Much more than usual	6	0.06<br>0.03 to 0.14	89	0.03<br>0.02 to 0.03	2	0.01<br>0.00 to 0.04	3	0.01<br>0.00 to 0.04	2	0.01<br>0.00 to 0.02
**Item 3: Have you recently felt you were playing a useful part in things?**										

For peer review only

More so than usual	14	0.15<br>0.09 to 0.25	291	0.10<br>0.09 to 0.11	26	0.13<br>0.09 to 0.19	35	0.11<br>0.08 to 0.16	27	0.10<br>0.07 to 0.15
Same as usual	47	0.49<br>0.38 to 0.60	2533	0.80<br>0.79 to 0.82	171	0.78<br>0.71 to 0.84	257	0.78<br>0.73 to 0.83	215	0.82<br>0.77 to 0.87
Less useful than usual	28	0.31<br>0.22 to 0.42	274	0.08<br>0.07 to 0.09	15	0.09<br>0.05 to 0.15	27	0.10<br>0.07 to 0.15	16	0.07<br>0.04 to 0.11
Much less useful	3	0.04<br>0.01 to 0.13	66	0.02<br>0.01 to 0.03	0	N/A	1	0.00<br>0.00 to 0.02	2	0.01<br>0.00 to 0.03
**Item 4: Have you recently felt capable of making decisions about things?**										
More so than usual	5	0.05<br>0.02 to 0.13	231	0.08<br>0.07 to 0.10	13	0.08<br>0.04 to 0.14	20	0.07<br>0.04 to 0.11	20	0.07<br>0.05 to 0.12
Same as usual	77	0.86<br>0.76 to 0.92	2745	0.86<br>0.84 to 0.87	193	0.88<br>0.81 to 0.93	290	0.89<br>0.84 to 0.92	232	0.89<br>0.84 to 0.92
Less so than usual	10	0.09<br>0.05 to 0.18	171	0.05<br>0.04 to 0.06	6	0.04<br>0.02 to 0.09	10	0.04<br>0.02 to 0.08	8	0.04<br>0.02 to 0.08
Much less capable	0	N/A	23	0.01<br>0.00 to 0.01	0	N/A	0	N/A	0	N/A
**Item 5: Have you felt under constant strain recently?**										
Not at all	8	0.08<br>0.04 to 0.17	837	0.27<br>0.25 to 0.29	68	0.32<br>0.25 to 0.39	106	0.32<br>0.27 to 0.38	64	0.23<br>0.18 to 0.29
No more than usual	42	0.43<br>0.33 to 0.54	1773	0.56<br>0.54 to 0.58	114	0.53<br>0.45 to 0.60	168	0.52<br>0.46 to 0.58	143	0.54<br>0.47 to 0.60
Rather more than usual	33	0.38<br>0.28 to 0.50	466	0.14<br>0.13 to 0.16	27	0.15<br>0.10 to 0.21	42	0.15<br>0.11 to 0.20	49	0.21<br>0.16 to 0.28
Much more than usual	9	0.10<br>0.05 to 0.19	92	0.03<br>0.02 to 0.03	3	0.01<br>0.00 to 0.03	4	0.01<br>0.00 to 0.02	4	0.02<br>0.01 to 0.06
**Item 6: Have you recently felt you couldn't overcome your difficulties?**										
Not at all	31	0.31<br>0.22 to 0.42	1191	0.39<br>0.37 to 0.40	88	0.41<br>0.34 to 0.49	138	0.43<br>0.37 to 0.49	96	0.34<br>0.29 to 0.41
No more than usual	45	0.52 0.41 to 0.63	1680	0.52<br>0.51 to 0.54	107	0.51<br>0.43 to 0.58	155	0.49<br>0.43 to 0.54	148	0.59<br>0.52 to 0.65
Rather more than usual	14	0.15<br>0.09 to 0.24	241	0.07<br>0.06 to 0.08	15	0.07<br>0.04 to 0.12	24	0.08<br>0.05 to 0.12	16	0.07<br>0.04 to 0.11
Much more than usual	2	0.02<br>0.01 to 0.09	55	0.02<br>0.01 to 0.02	2	0.01<br>0.00 to 0.06	3	0.01<br>0.00 to 0.04	0	N/A
**Item 7: Have you recently been able to enjoy your normal day to day activities?**										
More so than usual	1	0.01<br>0.00 to 0.05	158	0.06<br>0.05 to 0.07	15	0.09<br>0.05 to 0.15	23	0.08<br>0.05 to 0.13	12	0.04<br>0.02 to 0.07
Same as usual	58	0.61<br>0.50 to 0.71	2537	0.80<br>0.78 to 0.82	174	0.79<br>0.71 to 0.85	256	0.77<br>0.71 to 0.82	220	0.84<br>0.78 to 0.88
Less so than usual	26	0.31<br>0.22 to 0.42	382	0.12<br>0.10 to 0.13	18	0.10<br>0.06 to 0.17	33	0.12<br>0.08 to 0.17	27	0.12<br>0.08 to 0.18
Much less than usual	7	0.07<br>0.03 to 0.15	88	0.02<br>0.02 to 0.03	4	0.02<br>0.01 to 0.05	8	0.02<br>0.01 to 0.05	1	0.00<br>0.00 to 0.02
**Item 8: Have you recently been able to face up to your problems?**										
More so than usual	7	0.08<br>0.04 to 0.16	154	0.06<br>0.05 to 0.07	9	0.06<br>0.03 to 0.12	15	0.06<br>0.03 to 0.10	9	0.04<br>0.02 to 0.08
Same as usual	73	0.78<br>0.68 to 0.86	2746	0.87<br>0.86 to 0.88	191	0.90<br>0.84 to 0.94	287	0.90<br>0.86 to 0.93	235	0.91<br>0.86 to 0.95
Less able than usual	12	0.14<br>0.08 to 0.24	198	0.06<br>0.05 to 0.07	6	0.03<br>0.01 to 0.06	10	0.04<br>0.02 to 0.07	12	0.05<br>0.02 to 0.09
Much less able	0	N/A	29	0.01<br>0.01 to 0.01	1	0.01<br>0.00 to 0.06	1	0.01<br>0.00 to 0.04	0	N/A
**Item 9: Have you recently been feeling unhappy and depressed?**										
Not at all	29	0.31<br>0.21 to 0.42	1263	0.41<br>0.39 to 0.43	96	0.46<br>0.38 to 0.53	151	0.48<br>0.42 to 0.54	104	0.38<br>0.31 to 0.44
No more than usual	38	0.43<br>0.32 to 0.54	1420	0.45<br>0.43 to 0.47	90	0.43<br>0.36 to 0.51	130	0.41<br>0.35 to 0.47	129	0.54<br>0.47 to 0.60
Rather more than usual	25	0.26<br>0.18 to 0.37	366	0.12<br>0.10 to 0.13	20	0.11<br>0.07 to 0.17	28	0.10<br>0.07 to 0.14	23	0.09<br>0.06 to 0.13

Much more than usual	0	N/A	75	0.02 0.02 to 0.03	1	0.00 0.00 to 0.03	3	0.01 0.00 to 0.04	0	N/A
**Item 10: Have you recently been losing confidence in yourself?**										
**Not at all**	38	0.39 0.29 to 0.51	1510	0.49 0.47 to 0.51	113	0.53 0.46 to 0.61	176	0.55 0.49 to 0.61	132	0.48 0.42 to 0.55
No more than usual	39	0.43 0.33 to 0.54	1290	0.41 0.39 to 0.43	80	0.39 0.32 to 0.47	116	0.37 0.32 to 0.43	106	0.44 0.38 to 0.51
Rather more than usual	15	0.17 0.10 to 0.28	263	0.09 0.07 to 0.10	11	0.07 0.04 to 0.13	17	0.07 0.04 to 0.12	18	0.07 0.04 to 0.12
Much more than usual	0	N/A	58	0.02 0.01 to 0.02	1	0.00 0.00 to 0.03	2	0.01 0.00 to 0.03	0	N/A
**Item 11: Have you recently been thinking of yourself as a worthless person?**										
Not at all	54	0.57 0.45 to 0.67	2128	0.69 0.67 to 0.70	152	0.72 0.65 to 0.78	231	0.73 0.67 to 0.78	184	0.70 0.64 to 0.76
No more than usual	29	0.32 0.23 to 0.43	810	0.25 0.24 to 0.27	43	0.20 0.15 to 0.27	64	0.20 0.16 to 0.26	65	0.27 0.21 to 0.34
Rather more than usual	9	0.11 0.06 to 0.20	134	0.04 0.04 to 0.05	12	0.08 0.04 to 0.14	17	0.07 0.04 to 0.11	6	0.02 0.01 to 0.05
Much more than usual	0	N/A	53	0.02 0.01 to 0.02	0	N/A	1	0.00 0.00 to 0.03	1	0.00 0.00 to 0.03
**Item 12: Have you recently been feeling reasonably happy, all things considered?**										
More so than usual	5	0.06 0.02 to 0.14	310	0.11 0.10 to 0.12	18	0.11 0.07 to 0.17	26	0.10 0.06 to 0.14	29	0.12 0.08 to 0.18
About same as usual	67	0.71 0.60 to 0.80	2510	0.80 0.78 to 0.81	175	0.82 0.75 to 0.87	266	0.83 0.77 to 0.87	215	0.83 0.77 to 0.87
Less so than usual	20	0.23 0.15 to 0.33	243	0.07 0.06 to 0.08	13	0.06 0.03 to 0.10	18	0.06 0.04 to 0.09	11	0.05 0.02 to 0.08
Much less than usual	0	N/A	59	0.02 0.01 to 0.02	1	0.01 0.00 to 0.06	4	0.02 0.01 to 0.05	1	0.00 0.00 to 0.02
**Presence of probable mental ill health**										
No evidence of probable MIH	26	0.29 0.20 to 0.40	2009	0.56 0.55 to 0.58	143	0.58 0.51 to 0.65	220	0.58 0.52 to 0.64	168	0.58 0.52 to 0.65
Less than optimal MIH	39	0.41 0.30 to 0.52	665	0.19 0.17 to 0.20	44	0.22 0.16 to 0.28	61	0.19 0.15 to 0.24	69	0.27 0.21 to 0.33
MIH	27	0.30 0.21 to 0.41	904	0.25 0.23 to 0.26	49	0.20 0.15 to 0.27	78	0.23 0.18 to 0.28	45	0.15 0.11 to 0.20

Weighted proportion (WP) with the corresponding 95% Confidence Intervals (CI).

Key: MP: Member of Parliament Sample; EN: English Population (HSE 2014); CM: Corporate Managers (HSE 2014); AM: All managers (HSE 2014); HIG: high-income group (HSE 2014).

For peer review only

3. TABLE S2

Table S2: Descriptive characteristics of the 12 item GHQ (GHQ-12) and the four different predetermined HSE 2014 occupational and sociodemographic comparator groups (EN, CM, AM, HIG) - for Females

	n	WP	n	WP	n	WP	n	WP	n	WP
		MP		EN		CM		AM		HIG
		95% CI		95% CI		95% CI		95% CI		95% CI
**Age**										
21-30	2	0.00<br>0.00 to 0.01	681	0.20<br>0.19 to 0.22	21	0.13<br>0.08 to 0.21	36	0.13<br>0.09 to 0.19	26	0.21<br>0.14 to 0.29
31-40	8	0.08<br>0.04 to 0.17	784	0.17<br>0.16 to 0.19	45	0.16<br>0.12 to 0.21	72	0.17<br>0.14 to 0.21	55	0.32<br>0.25 to 0.40
41-50	17	0.32<br>0.20 to 0.46	845	0.18<br>0.17 to 0.19	66	0.24<br>0.19 to 0.29	83	0.20<br>0.16 to<br>0.24	30	0.18<br>0.12 to 0.25
51-60	21	0.54<br>0.39 to 0.68	726	0.16<br>0.15 to<br>0.17	51	0.21<br>0.16 to<br>0.28	80	0.21<br>0.17 to 0.26	21	0.12<br>0.08 to 0.18
61-70	5	0.06<br>0.02 to 0.14	681	0.13<br>0.12 to 0.14	44	0.15<br>0.11 to 0.19	71	0.16<br>0.13 to 0.20	20	0.11<br>0.07 to 0.17
70 +	1	0.00<br>0.00 to 0.01	722	0.15<br>0.14 to 0.16	32	0.11<br>0.08 to<br>0.15	55	0.12<br>0.10 to 0.16	11	0.06<br>0.03 to 0.11
**Educational attainment**										
NVQ4/NVQ5/Degree	0	N/A	1106	0.27<br>0.25 to<br>0.28	84	0.33<br>0.27 to 0.40	115	0.30<br>0.25 to 0.35	14<br>1	0.88<br>0.82 to 0.92
Higher ed below degree	47	0.05<br>0.01 to 0.17	483	0.10<br>0.09 to<br>0.11	39	0.15<br>0.11 to<br>0.20	58	0.14<br>0.11 to 0.18	5	0.03<br>0.01 to 0.06
NVQ3/GCE A Level	3	0.02<br>0.01 to 0.09	678	0.17<br>0.16 to 0.18	37	0.16<br>0.11 to<br>0.24	61	0.17<br>0.13 to<br>0.23	6	0.04<br>0.02 to 0.08
NVQ2/GCE O Level	2	0.01<br>0.00 to 0.07	878	0.19<br>0.18 to 0.21	58	0.22<br>0.17 to 0.27	88	0.21<br>0.17 to<br>0.25	6	0.03<br>0.02 to 0.08
NVQ1/CSE other grade	2	N/A	125	0.03<br>0.02 to<br>0.03	7	0.02<br>0.01 to<br>0.05	9	0.02<br>0.01 to 0.04	1	0.01<br>0.00 to 0.04
Foreign/other	0	N/A	95	0.02<br>0.02 to 0.02	4	0.01<br>0.00 to 0.03	9	0.02<br>0.01 to<br>0.04	2	0.01<br>0.00 to 0.04
No qualification	0	N/A	1060	0.22<br>0.21 to<br>0.24	30	0.10<br>0.07 to 0.15	57	0.13<br>0.10 to<br>0.17	2	0.01<br>0.00 to 0.03
**GHQ -12**										
**Item 1: Have you recently been able to concentrate on whatever you're doing?**										
Better than usual	3	0.05<br>0.01 to 0.15	123	0.03<br>0.02 to<br>0.04	10	0.04<br>0.02 to<br>0.07	15	0.04<br>0.02 to<br>0.06	3	0.02<br>0.01 to 0.06
Same as usual	32	0.61<br>0.46 to 0.74	3327	0.83<br>0.82 to 0.85	201	0.86<br>0.81 to 0.90	312	0.87<br>0.83 to 0.90	13<br>2	0.87<br>0.81 to 0.92
Less than usual	14	0.25<br>0.14 to 0.39	487	0.12<br>0.11 to 0.13	26	0.11<br>0.07 to 0.15	35	0.09<br>0.07 to 0.13	15	0.10<br>0.06 to 0.17
Much less than usual	5	0.09<br>0.04 to 0.21	63	0.01<br>0.01 to 0.02	0	N/A	0	N/A	1	0.01<br>0.00 to 0.04
**Item 2: Have you recently lost much sleep over worry?**										

Not at all	6	0.12 0.05 to 0.26	1123	0.28 0.27 to 0.30	65	0.27 0.21 to 0.34	98	0.26 0.22 to 0.32	38	0.25 0.18 to 0.33
No more than usual	24	0.45 0.31 to 0.60	2054	0.51 0.50 to 0.53	132	0.56 0.49 to 0.63	204	0.57 0.52 to 0.63	84	0.56 0.48 to 0.64
Rather more than usual	12	0.23 0.13 to 0.37	683	0.17 0.16 to 0.18	36	0.15 0.11 to 0.20	53	0.14 0.11 to 0.18	25	0.17 0.12 to 0.24
Much more than usual	12	0.19 0.10 to 0.33	151	0.03 0.03 to 0.04	5	0.02 0.01 to 0.05	8	0.02 0.01 to 0.04	4	0.02 0.01 to 0.06
**Item 3: Have you recently felt you were playing a useful part in things?**										
More so than usual	13	0.28 0.16 to 0.43	385	0.10 0.09 to 0.11	32	0.18 0.12 to 0.27	48	0.16 0.12 to 0.23	12	0.09 0.05 to 0.15
Same as usual	20	0.38 0.25 to 0.53	3163	0.79 0.78 to 0.81	191	0.76 0.68 to 0.83	291	0.77 0.71 to 0.82	124	0.80 0.72 to 0.86
Less useful than usual	15	0.26 0.15 to 0.40	351	0.08 0.08 to 0.09	11	0.05 0.03 to 0.08	20	0.05 0.03 to 0.08	14	0.10 0.06 to 0.17
Much less useful	6	0.09 0.03 to 0.20	91	0.02 0.02 to 0.03	3	0.01 0.00 to 0.04	3	0.01 0.00 to 0.03	1	0.01 0.00 to 0.05
**Item 4: Have you recently felt capable of making decisions about things?**										
More so than usual	4	0.07 0.03 to 0.19	278	0.07 0.06 to 0.08	16	0.06 0.04 to 0.10	22	0.06 0.04 to 0.09	8	0.07 0.03 to 0.13
Same as usual	41	0.78 0.65 to 0.88	3417	0.85 0.83 to 0.86	210	0.89 0.84 to 0.92	323	0.89 0.86 to 0.92	135	0.88 0.81 to 0.93
Less so than usual	7	0.10 0.04 to 0.21	273	0.07 0.06 to 0.08	11	0.04 0.02 to 0.08	17	0.04 0.03 to 0.07	8	0.05 0.03 to 0.10
Much less capable	2	0.04 0.01 to 0.17	43	0.01 0.01 to 0.01	1	0.01 0.00 to 0.04	1	0.00 0.00 to 0.02	0	N/A
**Item 5: Have you felt under constant strain recently?**										
Not at all	1	0.02 0.00 to 0.13	941	0.24 0.23 to 0.25	62	0.24 0.19 to 0.31	88	0.23 0.19 to 0.28	30	0.20 0.14 to 0.28
No more than usual	18	0.34 0.22 to 0.49	2201	0.55 0.53 to 0.57	129	0.55 0.47 to 0.62	206	0.57 0.52 to 0.63	93	0.63 0.54 to 0.70
Rather more than usual	20	0.39 0.26 to 0.54	726	0.18 0.17 to 0.19	42	0.19 0.14 to 0.26	60	0.17 0.13 to 0.22	26	0.16 0.11 to 0.22
Much more than usual	15	0.25 0.15 to 0.40	133	0.03 0.02 to 0.03	4	0.02 0.01 to 0.05	8	0.02 0.01 to 0.04	2	0.01 0.00 to 0.06
**Item 6: Have you recently felt you couldn't overcome your difficulties?**										
Not at all	10	0.20 0.11 to 0.34	1468	0.37 0.35 to 0.39	95	0.39 0.32 to 0.46	140	0.37 0.32 to 0.43	60	0.40 0.32 to 0.48
No more than usual	31	0.56 0.41 to 0.69	2082	0.52 0.50 to 0.53	127	0.55 0.47 to 0.62	197	0.56 0.50 to 0.61	81	0.54 0.46 to 0.62
Rather more than usual	10	0.18 0.09 to 0.32	361	0.09 0.08 to 0.10	16	0.07 0.04 to 0.11	24	0.07 0.04 to 0.10	7	0.05 0.02 to 0.09
Much more than usual	3	0.06 0.02 to 0.19	88	0.02 0.02 to 0.03	0	N/A	2	0.00 0.00 to 0.02	2	0.01 0.00 to 0.06
**Item 7: Have you recently been able to enjoy your normal day to day activities?**										
More so than usual	5	0.08 0.03 to 0.20	218	0.06 0.05 to 0.07	20	0.13 0.07 to 0.22	24	0.09 0.06 to 0.16	11	0.08 0.04 to 0.15
Same as usual	30	0.58 0.43 to 0.71	3112	0.78 0.76 to 0.79	184	0.74 0.66 to 0.81	288	0.77 0.71 to 0.82	124	0.82 0.75 to 0.88
Less so than usual	10	0.17	542	0.13	29	0.11	45	0.12	13	0.08

		0.09 to 0.31		0.12 to 0.14		0.08 to 0.16		0.09 to 0.15		0.05 to 0.14
Much less than usual	9	0.17 0.08 to 0.30	137	0.03 0.03 to 0.04	5	0.02 0.01 to 0.05	6	0.02 0.01 to 0.04	3	0.02 0.01 to 0.06
**Item 8: Have you recently been able to face up to your problems?**										
More so than usual	2	0.04 0.01 to 0.17	186	0.05 0.04 to 0.06	10	0.07 0.03 to 0.16	15	0.06 0.03 to 0.12	8	0.06 0.03 to 0.13
Same as usual	45	0.83 0.69 to 0.91	3411	0.86 0.85 to 0.87	213	0.89 0.81 to 0.94	323	0.90 0.84 to 0.93	137	0.90 0.83 to 0.94
Less able than usual	7	0.13 0.06 to 0.26	312	0.08 0.07 to 0.08	9	0.04 0.02 to 0.08	17	0.05 0.03 to 0.07	5	0.03 0.01 to 0.07
Much less able	0	N/A	43	0.01 0.01 to 0.01	0	N/A	0	N/A	1	0.01 0.00 to 0.05
**Item 9: Have you recently been feeling unhappy and depressed?**										
Not at all	14	0.26 0.16 to 0.41	1583	0.40 0.38 to 0.41	117	0.49 0.42 to 0.56	167	0.46 0.40 to 0.52	64	0.42 0.34 to 0.50
No more than usual	21	0.38 0.25 to 0.53	1699	0.43 0.42 to 0.45	88	0.40 0.33 to 0.48	141	0.42 0.36 to 0.47	73	0.49 0.41 to 0.58
Rather more than usual	19	0.35 0.23 to 0.50	545	0.13 0.12 to 0.15	24	0.10 0.07 to 0.15	42	0.11 0.08 to 0.15	11	0.07 0.04 to 0.13
Much more than usual	0	N/A	131	0.03 0.03 to 0.04	2	0.01 0.00 to 0.03	4	0.01 0.00 to 0.03	3	0.02 0.01 to 0.06
**Item 10: Have you recently been losing confidence in yourself?**										
Not at all	15	0.29 0.17 to 0.44	1682	0.42 0.40 to 0.44	119	0.52 0.45 to 0.59	173	0.49 0.43 to 0.55	69	0.45 0.37 to 0.54
No more than usual	26	0.49 0.35 to 0.63	1689	0.43 0.41 to 0.44	95	0.41 0.35 to 0.48	145	0.41 0.36 to 0.47	68	0.45 0.37 to 0.53
Rather more than usual	13	0.22 0.13 to 0.36	476	0.12 0.11 to 0.13	13	0.05 0.03 to 0.09	29	0.08 0.06 to 0.11	14	0.10 0.06 to 0.17
Much more than usual	0	N/A	112	0.03 0.02 to 0.04	4	0.02 0.01 to 0.04	7	0.02 0.01 to 0.04	0	N/A
**Item 11: Have you recently been thinking of yourself as a worthless person?**										
Not at all	32	0.60 0.45 to 0.73	2561	0.64 0.63 to 0.66	171	0.74 0.68 to 0.80	249	0.71 0.65 to 0.75	101	0.67 0.58 to 0.74
No more than usual	15	0.29 0.17 to 0.44	1069	0.27 0.26 to 0.29	52	0.23 0.18 to 0.29	90	0.26 0.21 to 0.31	42	0.27 0.20 to 0.35
Rather more than usual	7	0.11 0.05 to 0.24	244	0.06 0.05 to 0.07	4	0.02 0.01 to 0.04	9	0.02 0.01 to 0.05	7	0.05 0.02 to 0.11
Much more than usual	0	N/A	80	0.02 0.02 to 0.02	3	0.01 0.00 to 0.04	5	0.01 0.00 to 0.03	1	0.01 0.00 to 0.05
**Item 12: Have you recently been feeling reasonably happy, all things considered?**										
More so than usual	11	0.19 0.10 to 0.32	388	0.10 0.09 to 0.11	27	0.15 0.10 to 0.23	40	0.13 0.09 to 0.19	10	0.07 0.04 to 0.12
About same as usual	29	0.54 0.40 to 0.68	3123	0.79 0.77 to 0.80	189	0.79 0.71 to 0.85	287	0.79 0.74 to 0.84	131	0.86 0.80 to 0.91
Less so than usual	14	0.27 0.16 to 0.42	368	0.09 0.08 to 0.10	12	0.05 0.03 to 0.08	24	0.06 0.04 to 0.09	9	0.06 0.03 to 0.11
Much less than usual	0	N/A	78	0.02 0.02 to 0.03	3	0.01 0.00 to 0.04	3	0.01 0.00 to 0.03	1	0.01 0.00 to 0.05

**Presence of probable mental ill health**										
No evidence of probable MIH	9	0.19<br>0.10 to 0.34	2247	0.51<br>0.49 to 0.52	147	0.57<br>0.51 to 0.64	226	0.58<br>0.53 to 0.63	86	0.52<br>0.44 to 0.60
Less than optimal MIH	23	0.40<br>0.27 to 0.54	955	0.22<br>0.20 to 0.23	53	0.19<br>0.15 to 0.24	79	0.19<br>0.15 to 0.23	48	0.29<br>0.22 to 0.36
MIH	22	0.41<br>0.27 to 0.56	1237	0.28<br>0.26 to 0.29	59	0.24<br>0.18 to 0.30	92	0.23<br>0.19 to 0.28	29	0.20<br>0.14 to 0.27

Weighted proportion (WP) with the corresponding 95% Confidence Intervals (CI).

Key: MP: Member of Parliament Sample; EN: English Population (HSE 2014); CM: Corporate Managers (HSE 2014); AM: All managers (HSE 2014); HIG: high-income group (HSE 2014).

For peer review only

4. TABLE S3

Table S3. Crude and adjusted associations of mental health in relation to job status (having a job outside the parliament vs. not) of members of the parliament

GHQ-12 Items (n=146)	Crude		Adjusted±	
	**OR**	**95%CI**	**OR**	**(95% CI)**
Have you recently been able to concentrate on whatever you're doing?	0·6	0.23 to 1.57	0.74	0.27 to 2.04
Have you recently lost much sleep over worry?	0·64	0.26 to 1.58	0.73	0.28 to 1.90
Have you recently felt you were playing a useful part in things?	1.52	0.70 to 3.28	1.62	0.70 to 3.74
Have you recently felt capable of making decisions about things?	0·98	0.37 to 2.56	1.17	0.42 to 3.27
Have you felt under constant strain recently?	0·59	0.26 to 1.34	0.71	0.32 to 1.59
Have you recently felt you couldn't overcome your difficulties?	0·74	0.36 to 1.50	0.87	0.41 to 1.85
Have you recently been able to enjoy your normal day to day activities?	1.01	0.43 to 2.37	0.96	0.36 to 2.57
Have you recently been able to face up to your problems	1.04	0.37 to 2.93	0.98	0.36 to 2.69
Have you recently been feeling unhappy and depressed?	0·66	0.31 to 1.41	0.82	0.35 to 1.92
Have you recently been losing confidence in yourself?	1.02	0.37 to 2.69	1.29	0.46 to 3.60
Have you recently been thinking of yourself as a worthless person?	1.01	0.41 to 2.43	1.2	0.45 to 3.21
Presence of Common Mental Disorders	0.77	0.47 to 1.26	0.82	0.49 to 1.36
	**MD**	**95%CI**	**MD**	**95%CI**
**Total Score of GHQ to 12**	-.61	-3.06 to 1.84	-0·07	-2.44 to 2.31

Crude and Adjusted Odds Ratio (ORs) and Mean Difference (MD) with corresponding 95% Confidence Intervals (95% CI). Inverse probability weights were used with reference to the total number of the members of the parliament. All models were adjusted for age, sex and educational status

For peer review only

STROBE Statement—Checklist of items that should be included in reports of ***cross-sectional studies***

Item	Item No	Recommendation	Page No
**Title and abstract**	1	(*a*) Indicate the study's design with a commonly used term in the title or the abstract	1
		(*b*) Provide in the abstract an informative and balanced summary of what was done and what was found	3
**Introduction**			
Background/rationale	2	Explain the scientific background and rationale for the investigation being reported	4-5
Objectives	3	State specific objectives, including any prespecified hypotheses	5
**Methods**			
Study design	4	Present key elements of study design early in the paper	5
Setting	5	Describe the setting, locations, and relevant dates, including periods of recruitment, exposure, follow-up, and data collection	5-6
Participants	6	(*a*) Give the eligibility criteria, and the sources and methods of selection of participants	5
Variables	7	Clearly define all outcomes, exposures, predictors, potential confounders, and effect modifiers. Give diagnostic criteria, if applicable	7
Data sources/ measurement	8*	For each variable of interest, give sources of data and details of methods of assessment (measurement). Describe comparability of assessment methods if there is more than one group	6-7
Bias	9	Describe any efforts to address potential sources of bias	5-6
Study size	10	Explain how the study size was arrived at	5, 8
Quantitative variables	11	Explain how quantitative variables were handled in the analyses. If applicable, describe which groupings were chosen and why	8-9
Statistical methods	12	(*a*) Describe all statistical methods, including those used to control for confounding	8-9
		(*b*) Describe any methods used to examine subgroups and interactions	8-9
		(*c*) Explain how missing data were addressed	8-9
		(*d*) If applicable, describe analytical methods taking account of sampling strategy	8-9
		(*e*) Describe any sensitivity analyses	8-9
**Results**			
Participants	13*	(a) Report numbers of individuals at each stage of study—eg numbers potentially eligible, examined for eligibility, confirmed eligible, included in the study, completing follow-up, and analysed	9
		(b) Give reasons for non-participation at each stage	n.a.
		(c) Consider use of a flow diagram	n.a.
Descriptive data	14*	(a) Give characteristics of study participants (eg demographic, clinical, social) and information on exposures and potential confounders	9
		(b) Indicate number of participants with missing data for each variable of interest	n.a.
Outcome data	15*	Report numbers of outcome events or summary measures	9-12
Main results	16	(*a*) Give unadjusted estimates and, if applicable, confounder-adjusted estimates and their precision (eg, 95% confidence interval). Make clear	10-12

For peer review only - http://bmjopen.bmj.com/site/about/guidelines.xhtml

		which confounders were adjusted for and why they were included	
		(*b*) Report category boundaries when continuous variables were categorized	10-12
		(*c*) If relevant, consider translating estimates of relative risk into absolute risk for a meaningful time period	N.a.
Other analyses	17	Report other analyses done—eg analyses of subgroups and interactions, and sensitivity analyses	12-13
**Discussion**			
Key results	18	Summarise key results with reference to study objectives	13-14
Limitations	19	Discuss limitations of the study, taking into account sources of potential bias or imprecision. Discuss both direction and magnitude of any potential bias	14-15
Interpretation	20	Give a cautious overall interpretation of results considering objectives, limitations, multiplicity of analyses, results from similar studies, and other relevant evidence	16
Generalisability	21	Discuss the generalisability (external validity) of the study results	15-17
**Other information**			
Funding	22	Give the source of funding and the role of the funders for the present study and, if applicable, for the original study on which the present article is based	18

*Give information separately for exposed and unexposed groups.

Note: An Explanation and Elaboration article discusses each checklist item and gives methodological background and published examples of transparent reporting. The STROBE checklist is best used in conjunction with this article (freely available on the Web sites of PLoS Medicine at http://www.plosmedicine.org/, Annals of Internal Medicine at http://www.annals.org/, and Epidemiology at http://www.epidem.com/). Information on the STROBE Initiative is available at www.strobe-statement.org.

For peer review only

BMJ Open

Mental health of UK Members of Parliament in the House of Commons: a cross-sectional survey

Journal:	*BMJ Open*
Manuscript ID	bmjopen-2018-027892.R2
Article Type:	Research
Date Submitted by the Author:	15-May-2019

Complete List of Authors:	Poulter, Daniel; House of Commons<br>Votruba, Nicole; King's College London, Institute of Psychiatry, Psychology and Neuroscience;<br>Bakolis, Ioannis; King\'s College London, Department of Biostatistics and Health Informatics & Health Services and Population Research Department, Institute of Psychiatry, Psychology and Neuroscience<br>Debell, Frances; South London and Maudsley NHS Foundation Trust<br>Das-Munshi, Jayati; King's College London, Institute of Psychiatry, Section of Epidemiology, Department of Health Service & Population Research<br>Thornicroft, Graham; institute of psychiatry, Health service and population research/ Centre for Global Health

**Primary Subject Heading**:	Mental health
Secondary Subject Heading:	Public health, Mental health, Health services research, Health policy, Global health
Keywords:	MENTAL HEALTH, United Kingdom, policy making, stigma, Members of Parliament, MP

SCHOLARONE™
Manuscripts

Mental health of UK Members of Parliament in the House of Commons: a cross-sectional survey

Daniel Poulter[1]*, Nicole Votruba[2]*, Ioannis Bakolis[3],

Frances Debell[4], Jayati Das-Munshi[5] and Graham Thornicroft[6]

November 2018

* Joint first authors

Affiliations

[1] House of Commons, UK Parliament, House of Commons, London SW1A 0AA, UK; South London and Maudsley, Denmark Hill, London SE5 8AZ, UK; King's College London Policy Institute, 22 Kingsway, London, WC2B 6LE, UK, Dr Daniel Poulter, Visiting Professor

[2] Health Service & Population Research Department, Institute of Psychiatry, Psychology and Neuroscience, King's College London, De Crespigny Park, London SE5 8AF, UK, Nicole Votruba, Researcher

[3] Department of Biostatistics and Health Informatics & Health Services and Population Research Department, Institute of Psychiatry, Psychology and Neuroscience, King's College London, De Crespigny Park, London SE5 8AF, UK, Dr Ioannis Bakolis, Lecturer

[4] South London and Maudsley, Denmark Hill, London SE5 8AZ, UK, Dr Frances Debell

[5] Health Service & Population Research Department, Institute of Psychiatry, Psychology and Neuroscience, King's College London, De Crespigny Park, London SE5 8AF, UK, South London & Maudsley NHS Trust, UK, Dr Jayati Das-Munshi, Clinical Scientist Fellow (Honorary Consultant)

For peer review only

[6] Centre for Global Mental Health, and Centre for Implementation Science, Institute of Psychiatry, Psychology and Neuroscience, King's College London, De Crespigny Park, London SE5 8AF, UK, Sir Graham Thornicroft, Professor of Community Psychiatry

Correspondence to: Nicole Votruba Nicole.votruba@kcl.ac.uk +44 (0) 207 848 0619

Word count main text (excl. abstract, references, tables, boxes, figures): 3900

Key words: mental health, United Kingdom, policy making, stigma, Members of Parliament, MP

For peer review only

ABSTRACT

Objectives The purpose of this study was to assess: (i) overall mental health of Members of Parliament (MPs); and (ii) awareness among MPs of the mental health support services available to them in Parliament.

Design Anonymous, self-completed, online cross-sectional survey, conducted in December 2016.

Setting 56th UK House of Commons.

Participants All 650 members of the 56th UK House of Commons were invited to participate; 146 MPs (23%) completed the survey.

Outcomes The General Health Questionnaire-12 was used to assess age and sex standardised prevalence of probable common mental disorders (CMD). Results were compared to a nationally representative survey, the Health Survey for England 2014 (HSE). Core demographic questions, MPs' awareness of available mental health services, their willingness to discuss mental health issues with party whips and fellow MPs, and the effects of employment outside parliament, were assessed.

Results Comparison of MP respondents with HSE comparator groups found that MPs have higher rates of mental health problems (age and sex standardised prevalence of probable CMD in surveyed MPs 34% (n=49); (95% CI: 27% to 42%) versus 17%; (95% CI: 13% to 21%) in the high-income comparison group). Survey respondents were younger, more likely to be female and more educated, compared to all MPs. 77% of MPs (n=112) did not know how to access in house mental health support. 52% (n=76) would not discuss their mental health with party whips, or other MPs (48%; n=70).

Conclusions MPs in the study sample had higher rates of mental health problems than rates seen in the whole English population, or comparable occupational groups. Most surveyed MPs are unaware of mental health support services, or how to access them. Our findings represent a relatively small sample of MPs. There is a need for MPs to have better awareness of, and access to, mental health support.

For peer review only

STRENGTHS AND LIMITATIONS OF THIS STUDY

- This is a unique study where the mental health of MPs has been assessed using structured, validated scales for the first time.
- This study is also the first evaluation of MPs' awareness of the mental health support available to them from the Parliamentary Health and Wellbeing Service and how to access this service.
- This study also assessed for the first time the willingness of MPs to discuss any mental health issues with party whips or with fellow MPs.
- The survey had a relatively low response rate which may be related to the stigma associated with mental illness, and to the nature of an MP's role, which is associated with a stressful work schedule and life in the public eye.

For peer review only

INTRODUCTION

There is a public fascination with understanding the psyches of politicians and decision-makers, from ancient times to the present day, and a long history of public debate about the mental health of politicians, including discussion of the potential psychiatric diagnoses of notable individuals active in political life[1-9]. Research studies have considered some related questions, such as the harassment and stalking of politicians.[10-13] Studies have also examined media and public reactions to politicians' actual or perceived mental health problems. [14-17] Yet, little has been published on the actual mental health or mental illness of politicians. Some evidence of politicians disclosing personal mental health problems has been published, for example during the passage of the UK Mental Health (Discrimination) Act in 2013, which removed discriminatory provisions permitting disqualification of Members of Parliament with mental health problems under certain circumstances.[18].

A scoping literature search in January 2017 was conducted to understand what is known about politicians' mental health, and in particular the prevalence of common mental disorders in this group. The papers identified were largely limited to politicians in the UK, USA and Australasia. There remains a dearth of evidence on the prevalence of common mental disorders (CMDs) in politicians and how this compares to general population rates. To date, no quantitative, ethically approved surveys have been conducted of Members of Parliament (MPs) in the UK Parliament to assess their mental health, and to assess their awareness of the available support and treatment services.

Several factors in the UK political system may adversely influence MPs and their mental health: The UK Parliament permits MPs to hold employment outside Parliament in addition to their roles as elected representatives. Further, in the UK parliament, "whips" are appointed officials in each political party who are charged with organising their party's parliamentary business and ensuring party discipline amongst MPs. In addition, a confidential in-house service is provided within Parliament for MPs and peers, called the Parliamentary Health and Wellbeing Service, to support their occupational health and wellbeing.

For peer review only - http://bmjopen.bmj.com/site/about/guidelines.xhtml

In this context, the UK Parliamentary Mental Health (UKPMH) study aims are to: (i) assess the overall mental health of MPs by drawing comparisons with a nationally representative survey in England, and with comparator socio-demographic and occupational groups within the survey; and (ii) assess awareness among MPs of the mental health support services available to them.

The principal research question was: What is the prevalence of common mental disorders among MPs? The secondary questions addressed were: how far are MPs aware of mental health services that can assist them with mental health problems? Are MPs willing to discuss their mental health with party whips or other MPs? This study tested the following primary hypothesis: the occurrence of common mental disorders (CMDs) is higher among MPs compared to the general population and compared with specific socio-demographic, professional and occupational comparator groups.

METHODS

Study design and participants

We conducted an anonymised, online self-completed survey at the House of Commons in December 2016. The inclusion criteria for participation were: membership of the 56th UK Parliament, House of Commons; and providing written, informed consent. We followed the STROBE guidelines for observational studies for the reporting of this cross-sectional study.[19] No age limits were defined, except that to be elected to Parliament one must be over 18 years old. Participants were sent via email an invitation letter to participate. Initially, in November 2016 a letter was sent to all 650 members of the House of Commons to make them aware of the study. In early December, a letter including a web link to an online survey with an individual access code was sent out via to all MPs internal post, and via email. The survey took place between 5 and 31 December 2016. Repeated efforts were taken to promote participation and maximise response rates in the survey. The study information sheet (explaining the purpose of the study) and instructions for the online questionnaire, as well as two

For peer review only - http://bmjopen.bmj.com/site/about/guidelines.xhtml

reminder emails, were sent out with clear descriptions of encrypted data collection and protection measures to ensure anonymity.

Ethics and data protection

At all times throughout the study preparation, conduct and analysis, particular consideration and care has been given to the specific, sensitive study context, and to the potential vulnerability of participants, namely the risk of sensationalised coverage should any individual be identifiable. Ethics approval for the study was obtained in September 2016 from King's College London Ethics Committee (reference number: HR-16/17-3118). Efforts were taken to limit distress and secure confidentiality for the participants. To ensure full confidentiality no personal identifiers were collected, and identifiers were removed if provided. All participants were provided with contact information for the Parliamentary Health and Wellbeing Service in the introductory letter and via the online survey in case any participants were experiencing distress at the time of the survey.

Health Survey for England comparator groups

Data for the comparator groups were elicited from the Health Survey for England (HSE) 2014. The HSE is an annual survey which uses a multi-stage stratified design to sample nationally representative random cross section of the population of England each year. Participants are visited by an interviewer who collects demographic and socio-economic data, and information on health and health-related behaviours. A detailed description of the HSE has been reported elsewhere.[20] From the HSE, we identified four comparison groups: total population of England in the HSE England population (EN), corporate managers in England (CM), all managers in England (AM), and those in high-income groups in England (HIG). The socio-economic groups derive from a standardised questionnaire asked in the HSE to all survey respondents.

Measures of mental health

For peer review only

The General Health Questionnaire (GHQ-12) was used to assess the mental health of respondents in the UKPMH sample and the HSE 2014. The self-completed 12-item GHQ-12 is one of the most extensively used screening instruments for common mental disorders, measured by a 4-point Likert scale (ranging from ‘less than usual’ to ‘much more than usual’) across twelve items.[20, 21]

Scoring of the GHQ-12 for the present study was done in the original bi-modal method as developed by Goldberg.[22] Specifically, each symptom was scored either 0 if ’not at all present’ or present ‘no more than usual’, or 1 for symptoms that were present ‘rather more than usual’ or ‘much more than usual’). The scoring method allowed for total scores to range from 0 to 12. No formal threshold exists for identifying probable mental ill health, with optimal values likely to be specific to the population under study. However, in line with the previous HSE survey, MP’s total scores are grouped according to three categories: 0 (indicating no evidence of probable mental ill health), 1 to 3 (indicating less than optimal mental health), and 4 or more (indicating probable psychological disturbance or mental ill health).[20, 21]

The GHQ-12 has been extensively validated across international settings for screening and detection of the common mental disorders.[23] In previous work, with a cut-off point ≧4, the total score of the GHQ-12 was found in a UK setting to have a sensitivity of 84.6% and specificity of 89.3% when assessed against *International Classification of Mental Disorders (ICD-10) and the Diagnostic Statistical Manuals-IV (DSM-IV),* diagnoses derived from the Composite International Diagnostic Interview (CIDI-PC) for the common mental disorders (including depression, dysthymia, generalised anxiety disorder, panic disorder and other related conditions).[23]

A technical error in the administration of the questionnaire caused a lack of indication for respondents of the 4th option (much more/much less than usual) on GHQ-12 items 8, 9, 10, 11, 12. However, this has no impact on the total scores of GHQ-12 for each participant, as the third and fourth option are grouped together in the bi-modal scoring.

For peer review only

In the question on awareness of the Parliamentary Health and Wellbeing Service, a technical error in the administration of the questionnaire caused 4 options (no/ unsure/ unaware/ yes) to be offered rather than binary yes and no options. The three options (no/ unsure/ unaware) were combined to represent “no awareness”.

Covariates

Core demographic questions were obtained from the UKPMH study sample: Age (categorised into five groups: 21 to 30; 31 to 40; 41 to 50; 51 to 60; 61 to 70, >70 years), sex (female or male), and educational status (GCSE/ O level, A Level, Vocational Qualifications, Undergraduate Degree, Post Graduate Degree, Doctorate), as well as years serving as MP. MPs were also asked if they were aware of the mental health services available to them, as well as their willingness to discuss their mental health with their Whips and other MPs (full list of questions in Supplementary File). Ethnicity was not assessed. Due to the low number of MPs from a minority ethnic background in the 56th House of Commons (n=41), this avoided any concern about the identification of participants, which may have further limited the response rate.

For peer review only

Statistical analyses

All statistical analyses were performed using STATA 14.1. Within the UKPMH sample, descriptive analysis was undertaken first to determine the distribution of each item of the GHQ-12 and of socio-demographic characteristics, awareness of mental health services, and willingness to discuss mental health issues with party whips or with fellow MPs.

The UKPMH sample is subject to “unit non-response” as 22.4% of all MPs completed the survey. To address this issue, we employed inverse probability weighting (IPW)[24] in the analysis, where weights are used to rebalance the set of complete cases within the MP sample to make it representative of the whole English population; we used the weighted sample of the HSE 2014. Age-sex standardised proportion estimates were calculated i) for each item of the GHQ-12, and ii) for the presence of

For peer review only - http://bmjopen.bmj.com/site/about/guidelines.xhtml

probable mental ill health. We compared i) each item of the GHQ-12, and ii) the three combined categories derived from the total score of the GHQ-12 that indicate the presence of probable mental ill health of the MP sample with a range of socio-demographic groups (the English population (EN), corporate managers (CM), all managers (AM), and with high income groups (HIG) in England) derived from HSE 2014. As a sensitivity analyses, age-sex standardised proportion estimates were calculated separately for males and females.

Non-parametric tests (chi-square) and parametric tests (t-test for unequal sample sizes) were employed to explore potential differences in the proportion estimates between UKPMH and HSE 2014 samples.

Cross-sectional associations of whether an MP had additional employment outside Parliament with each different item of the GHQ-12, and with the three combined categories (indicating no evidence of probable mental ill health, less than optimal mental health, probable psychological disturbance or mental ill health) were explored with the use of ordinal logistic regression models. Results were expressed as increased risk (odds ratio and corresponding 95% confidence intervals) of being in a highest category of each item of the GHQ-12 for those MPs with a work role outside parliament were compared to those without such an external role.

In addition, linear regression models were employed to explore the mean difference in the GHQ-12 total scores for those MPs who had additional employment outside Parliament, and for those who did not. All models were adjusted for the following potential confounders identified a priori: age, sex and educational status. Age-sex standardised inverse probability weights were employed for all linear and ordinal regression models.

Patient and Public Involvement

Daniel Poulter, MP, was involved at all stages of the study and is co-author of the paper. Other parliamentarians and staff of the Parliamentary Health and Wellbeing Service were consulted at the

For peer review only

planning and design stages, as well as at the interpretations of the findings and dissemination stages of the study.

RESULTS

Questionnaires were returned by 146 respondents (22.4%) of the 650 MPs. Median time to complete the survey was 4 minutes (IQR: 3 to 5). Most respondents were male (63%), with an undergraduate (44%) or a postgraduate degree (36%) or doctorate (2%). Most were between 41 and 60 years old (66%), and most did not work outside parliament (81%) (see Table 1).

Table 1: Demographic characteristics of UKPMH participants

	MP sample (N=146)	Total Health Survey for England sample (N=7871)
	n (%)	n (%)
Below 40 years old	27 (18%)	4014 (51%)
Female	54 (36%)	4385 (55%)
Higher education degree	119 (82%)	888 (11.3%)
Knowledge on how to access to mental health support	65 (45%)	n/a
Unaware of parliamentary well-being service	112 (77%)	n/a
Willing to discuss mental health problems with whips	70 (48%)	n/a
Willing to discuss mental health problems with other MPs	76 (52%)	n/a
Presence of CMD (according to ≥4 cut point on the GHQ-12 total score)	49 (34%)	2902 (26%)

Mental health of MPs and the HSE 2014 comparator groups

Table 2 presents weighted proportion estimates and corresponding 95% confidence intervals of the UKPMH sample and the four different predetermined HSE 2014 occupational and sociodemographic comparator groups (EN, CM, AM, HIG). For each item of the GHQ-12, the UKPMH sample presented a higher weighted proportion of participants who had lower levels of concentration, were losing sleep because of worry, were feeling less useful, were less capable of making decisions, and were feeling under constant strain, compared to the four HSE 2014 occupational and sociodemographic comparison groups (p-values of chi-square test <0.001).

For peer review only - http://bmjopen.bmj.com/site/about/guidelines.xhtml

In addition, a higher weighted proportion of MPs could not overcome difficulties, were less able to enjoy normal day to day activities, were less able to face up to their problems, reported losing confidence in themselves, or feeling unhappy and depressed, and more individual MPs considered themselves to be a worthless person (p-values of chi-square test <0.001). Compared to the HSE 2014 predetermined occupational and sociodemographic comparator groups, a higher weighted proportion of the MPs also reported being less able to feel reasonably happy (p-values of chi-square test <0.001).

When we compared the weighted proportions of the three combined categories derived for the GHQ-12 total score that indicate the presence of probable mental ill health between the UKPMH and HSE 2014 samples, we found that a higher proportion of MPs had probable mental ill health (weighted proportion: 34%; 95% CI: 27%, 42%), compared with EN (weighted proportion: 26%; 95% CI: 25%, 27%), CM (weighted proportion: 22%; 95% CI: 18%, 26%), AM (weighted proportion: 23%; 95% CI: 20%, 27%) and HIG (weighted proportion: 17%; 95% CI: 13% to 21%) (p-values of chi-square test <0.001) (see Table 2 and Figure 1). In addition, female MPs had higher rates of probable mental ill health (weighted proportion: 41%; 95% CI: 27%, 56%) compared to male MPs (weighted proportion: 30%; 95% CI: 21%, 41%) (see Supplementary File, Table S1 and Table S2).

Table 2: Descriptive characteristics of the 12 item GHQ (GHQ-12), and the four different predetermined HSE 2014 occupational and sociodemographic comparator groups (EN, CM, AM, HIG).

	n	WP<br>95%CI	n	WP<br>95% CI	n	WP<br>95% CI	n	WP<br>95% CI	n	WP<br>95% CI
		MP		EN		CM		AM		HIG
**Item 1: Have you recently been able to concentrate on whatever you're doing?**										
Better than usual	5	0.03<br>0.01 to 0.07	223	0.035<br>0.03 to 0.04	15	0.03<br>0.02 to 0.05	24	0.03<br>0.02 to 0.05	10	0.03<br>0.01 to 0.05
Same as usual	93	0.66<br>0.57 to 0.74	6073	0.85<br>0.84 to 0.86	394	0.88<br>0.84 to 0.91	602	0.88<br>0.85 to 0.91	371	0.9<br>0.87 to 0.93
Less than usual	40	0.26<br>0.19 to 0.34	771	0.1<br>0.10 to 0.11	38	0.08<br>0.06 to 0.11	53	0.08<br>0.06 to 0.10	29	0.07<br>0.05 to 0.10
Much less than usual	8	0.05<br>0.02 to 0.11	103	0.01<br>0.01 to 0.02	2	0.01<br>0.00 to 0.04	3	0.01<br>0.00 to 0.03	1	0.005<br>0.00 to 0.01
**Item 2: Have you recently lost much sleep over worry?**										
Not at all	24	0.18<br>0.12 to 0.26	2334	0.33<br>0.32 to 0.34	146	0.33<br>0.28 to 0.38	226	0.33<br>0.29 to 0.37	130	0.3<br>0.26 to 0.35
No more than usual	66	0.47<br>0.38 to 0.56	3573	0.5<br>0.49 to 0.51	246	0.54<br>0.49 to 0.59	370	0.55<br>0.50 to 0.59	220	0.56<br>0.51 to 0.61
Rather more than usual	38	0.26<br>0.19 to 0.34	1035	0.14<br>0.13 to 0.15	51	0.11<br>0.08 to 0.14	76	0.11<br>0.09 to 0.14	55	0.13<br>0.10 to 0.16

For peer review only

Much more than usual	18	0.1<br>0.06 to 0.16	240	0.03<br>0.02 to 0.04	7	0.02<br>0.01 to 0.03	11	0.02<br>0.01 to 0.03	6	0.01<br>0.00 to 0.03
**Item 3: Have you recently felt you were playing a useful part in things?**										
More so than usual	27	0.19<br>0.13 to 0.27	676	0.10<br>0.09 to 0.11	58	0.16<br>0.12 to 0.21	83	0.14<br>0.11 to 0.18	39	0.10<br>0.07 to 0.13
Same as usual	67	0.46<br>0.38 to 0.55	5696	0.8<br>0.79 to 0.81	362	0.77<br>0.72 to 0.81	548	0.78<br>0.74 to 0.81	339	0.82<br>0.77 to 0.85
Less useful than usual	43	0.3<br>0.22 to 0.39	625	0.08<br>0.07 to 0.09	26	0.07<br>0.05 to 0.10	47	0.08<br>0.06 to 0.10	30	0.08<br>0.05 to 0.12
Much less useful	9	0.05<br>0.02 to 0.11	157	0.02<br>0.01 to 0.03	3	0.005<br>0.00 to 0.02	4	0.005<br>0.00 to 0.02	3	0.01<br>0.00 to 0.02
**Item 4: Have you recently felt capable of making decisions about things?**										
More so than usual	9	0.06<br>0.03 to 0.11	509	0.08<br>0.07 to 0.09	29	0.07<br>0.05 to 0.11	42	0.07<br>0.05 to 0.09	28	0.07<br>0.05 to 0.10
Same as usual	118	0.84<br>0.77 to 0.89	6162	0.85<br>0.84 to 0.86	403	0.88<br>0.84 to 0.91	613	0.89<br>0.86 to 0.91	367	0.89<br>0.85 to 0.92
Less so than usual	17	0.09<br>0.05 to 0.15	444	0.066<br>0.06 to 0.08	17	0.04<br>0.02 to 0.07	27	0.04<br>0.03 to 0.06	16	0.04<br>0.02 to 0.07
Much less capable	2	0.01<br>0.00 to 0.05	66	0.01<br>0.01 to 0.01	1	0<br>0.00 to 0.02	1	0<br>0.00 to 0.01	0	NA
**Item 5: Have you felt under constant strain recently?**										
Not at all	9	0.07<br>0.03 to 0.13	1778	0.25<br>0.24 to 0.27	130	0.28<br>0.24 to 0.33	194	0.28<br>0.24 to 0.31	94	0.22<br>0.18 to 0.27
No more than usual	60	0.41<br>0.33 to 0.50	3974	0.56<br>0.54 to 0.57	243	0.54<br>0.49 to 0.59	374	0.55<br>0.51 to 0.59	236	0.57<br>0.51 to 0.62
Rather more than usual	53	0.38<br>0.30 to 0.47	1192	0.16<br>0.15 to 0.17	69	0.17<br>0.13 to 0.21	102	0.16<br>0.13 to 0.20	75	0.19<br>0.15 to 0.24
Much more than usual	24	0.14<br>0.09 to 0.21	225	0.03<br>0.02 to 0.03	7	0.02<br>0.01 to 0.03	12	0.02<br>0.01 to 0.03	6	0.02<br>0.01 to 0.04
**Item 6: Have you recently felt you couldn't overcome your difficulties?**										
Not at all	41	0.29<br>0.21 to 0.37	2659	0.38<br>0.37 to 0.39	183	0.4<br>0.35 to 0.45	278	0.4<br>0.36 to 0.44	156	0.36<br>0.31 to 0.41
No more than usual	76	0.52<br>0.44 to 0.61	3762	0.52<br>0.51 to 0.53	234	0.53<br>0.47 to 0.58	352	0.52<br>0.48 to 0.56	229	0.57<br>0.52 to 0.62
Rather more than usual	24	0.16<br>0.10 to 0.23	602	0.08<br>0.08 to 0.09	31	0.07<br>0.05 to 0.10	48	0.07<br>0.05 to 0.09	23	0.06<br>0.04 to 0.09
Much more than usual	5	0.03<br>0.01 to 0.08	143	0.02<br>0.02 to 0.02	2	0.01<br>0.00 to 0.03	5	0.01<br>0.00 to 0.02	2	0<br>0.00 to 0.02
**Item 7: Have you recently been able to enjoy your normal day to day activities?**										
More so than usual	6	0.03<br>0.01 to 0.06	376	0.06<br>0.05 to 0.07	35	0.11<br>0.07 to 0.16	47	0.09<br>0.06 to 0.13	23	0.05<br>0.04 to 0.08
Same as usual	88	0.61<br>0.52 to 0.69	5649	0.79<br>0.78 to 0.80	358	0.76<br>0.71 to 0.81	544	0.77<br>0.73 to 0.81	344	0.83<br>0.79 to 0.87
Less so than usual	36	0.27<br>0.19 to 0.36	924	0.12<br>0.12 to 0.13	47	0.11<br>0.08 to 0.14	78	0.12<br>0.09 to 0.15	40	0.11<br>0.08 to 0.15
Much less than usual	16	0.10<br>0.06 to 0.16	225	0.025<br>0.02 to 0.03	9	0.02<br>0.01 to 0.04	14	0.02<br>0.01 to 0.03	4	0.01<br>0.00 to 0.02
**Item 8: Have you recently been able to face up to your problems?**										
More so than usual	9	0.07<br>0.04 to 0.13	340	0.06<br>0.05 to 0.07	19	0.06<br>0.04 to 0.11	30	0.06<br>0.04 to 0.09	17	0.05<br>0.03 to 0.08
Same as usual	118	0.80<br>0.71 to 0.86	6157	0.87<br>0.86 to 0.88	404	0.90<br>0.85 to 0.93	610	0.9<br>0.86 to 0.92	372	0.91<br>0.87 to 0.94
Less able than usual	19	0.14<br>0.08 to 0.21	510	0.07<br>0.06 to 0.07	15	0.03<br>0.02 to 0.06	27	0.04<br>0.03 to 0.06	17	0.04<br>0.02 to 0.07
Much less able	NA	NA	72	0.01<br>0.01 to 0.01	1	0.01<br>0.00 to 0.03	1	0.01<br>0.00 to 0.02	1	0.01<br>0.00 to 0.02

For peer review only - http://bmjopen.bmj.com/site/about/guidelines.xhtml

**Item 9: Have you recently been feeling unhappy and depressed?**										
Not at all	43	0.3<br>0.22 to 0.38	2846	0.4<br>0.39 to 0.42	213	0.47<br>0.42 to 0.52	318	0.47<br>0.43 to 0.51	168	0.39<br>0.34 to 0.44
No more than usual	59	0.42<br>0.33 to 0.51	3119	0.44<br>0.43 to 0.45	178	0.42<br>0.37 to 0.47	271	0.41<br>0.37 to 0.46	202	0.52<br>0.47 to 0.58
Rather more than usual	44	0.29<br>0.21 to 0.37	911	0.13<br>0.12 to 0.15	44	0.1<br>0.08 to 0.14	70	0.11<br>0.08 to 0.13	34	0.08<br>0.06 to 0.11
Much more than usual	NA	NA	206	0.03<br>0.01 to 0.04	3	0.01<br>0.00 to 0.02	7	0.01<br>0.01 to 0.03	3	0.01<br>0.00 to 0.02
**Item 10: Have you recently been losing confidence in yourself?**										
Not at all	53	0.37<br>0.29 to 0.46	3192	0.45<br>0.44 to 0.47	232	0.52<br>0.47 to 0.58	349	0.52<br>0.48 to 0.56	201	0.47<br>0.42 to 0.53
No more than usual	65	0.45<br>0.36 to 0.54	2979	0.42<br>0.41 to 0.43	175	0.4<br>0.35 to 0.45	261	0.39<br>0.35 to 0.43	174	0.44<br>0.39 to 0.50
Rather more than usual	28	0.18<br>0.13 to 0.26	739	0.1<br>0.10 to 0.11	24	0.06<br>0.04 to 0.10	46	0.08<br>0.06 to 0.10	32	0.08<br>0.06 to 0.12
Much more than usual	NA	NA	170	0.02<br>0.02 to 0.03	5	0.01<br>0.00 to 0.02	9	0.015<br>0.01 to 0.02	NA	NA
**Item 11: Have you recently been thinking of yourself as a worthless person?**										
Not at all	86	0.58<br>0.49 to 0.66	4689	0.66<br>0.65 to 0.68	323	0.73<br>0.68 to 0.77	480	0.72<br>0.68 to 0.75	285	0.69<br>0.64 to 0.74
No more than usual	44	0.31<br>0.24 to 0.40	1879	0.26<br>0.25 to 0.27	95	0.22<br>0.18 to 0.26	154	0.23<br>0.20 to 0.27	107	0.27<br>0.23 to 0.32
Rather more than usual	16	0.11<br>0.06 to 0.18	378	0.05<br>0.05 to 0.06	16	0.05<br>0.03 to 0.08	26	0.05<br>0.03 to 0.07	13	0.03<br>0.02 to 0.06
Much more than usual	NA	N NA<br>A	133	0.02<br>0.02 to 0.02	3	0.01<br>0.00 to 0.02	6	0.01<br>0.00 to 0.02	2	0.01<br>0.00 to 0.02
**Item 12: Have you recently been feeling reasonably happy, all things considered?**										
More so than usual	16	0.09<br>0.05 to 0.15	698	0.11<br>0.10 to 0.11	45	0.13<br>0.09 to 0.18	66	0.12<br>0.09 to 0.15	39	0.11<br>0.08 to 0.14
About same as usual	96	0.67<br>0.59 to 0.75	5633	0.79<br>0.78 to 0.80	364	0.8<br>0.75 to 0.85	553	0.81<br>0.77 to 0.84	346	0.84<br>0.80 to 0.88
Less so than usual	34	0.24<br>0.17 to 0.32	611	0.08<br>0.08 to 0.09	25	0.05<br>0.04 to 0.08	42	0.06<br>0.04 to 0.08	20	0.05<br>0.03 to 0.08
Much less than usual	NA	NA	137	0.02<br>0.02 to 0.02	4	0.01<br>0.00 to 0.03	7	0.01<br>0.01 to 0.03	2	0<br>0.00 to 0.02
**Presence of probable mental ill health**										
No evidence of probable mental ill health	35	0.25<br>0.18 to 0.34	4256	0.53<br>0.52 to 0.55	290	0.58<br>0.53 to 0.62	446	0.58<br>0.54 to 0.62	254	0.56<br>0.51 to 0.61
Less than optimal mental ill health	62	0.40<br>0.32 to 0.49	1620	0.2<br>0.19 to 0.21	97	0.2<br>0.17 to 0.25	140	0.19<br>0.16 to 0.22	117	0.27<br>0.23 to 0.32
Probable mental ill health	49	0.34<br>0.27 to 0.43	2141	0.26<br>0.25 to 0.27	108	0.22<br>0.18 to 0.26	170	0.23<br>0.20 to 0.27	74	0.17<br>0.13 to 0.21

Weighted proportion (WP) with the corresponding 95% Confidence Intervals (CI).

Key: MP: Member of Parliament Sample; EN: English Population (HSE 2014); CM: Corporate Managers (HSE 2014); AM: All managers (HSE 2014); HIG: high-income group (HSE 2014).

For peer review only - http://bmjopen.bmj.com/site/about/guidelines.xhtml

Characteristics of respondents in comparison to all MPs

Compared with all 650 MPs, those who participated were younger (18 %, n=27 vs. 16% of total MP population were below 40 years old), more likely to be female (37%, n=54 of the UKPMH sample vs 30% of total MPs population were female) in relation to the gender distribution of the total number of MPs, and more educated (81%, n=119) of the UKPMH sample had a university degree vs. 76% of total MP population.

Awareness of mental health support services

Most MPs were not aware of the mental health services provided by the Parliamentary Health and Wellbeing Service within parliament. Most MPs (55 %) did not know how to access any mental health support at Parliament (see Figure 2). When asked whether they felt the Parliamentary Health and Wellbeing Service currently offered sufficient support, a large majority of MPs (77%) were unaware of what options are currently offered by the service and only 23% were aware that support was sufficiently available (see Figure 3).

(Figures 2, 3, 4, 5 about here)

Willingness to disclose poor mental health

Most MPs who took part in this survey were not willing to discuss mental health problems with their party whips (52%), and only a small majority of MPs would feel able to talk with other MPs about their mental health (52%) (see Figures 4 and 5). After adjusting for age, sex and educational status, we found evidence that MPs who were willing to discuss their mental health with their party whips or fellow MPs, had a reduced risk of CMDs (willing to discuss with whips: adjusted OR: 0.32; (95% CI: 0.16, 0.31), or discuss with fellow MPs: adjusted OR: 0.57; (95% CI: 0.30, 0.99) .

Additional employment outside parliament

For peer review only - http://bmjopen.bmj.com/site/about/guidelines.xhtml

We found no evidence of an association between having additional employment outside Parliament with the individual GHQ-12 items, or an increased total GHQ score indicating poor mental health (see Supplementary File, Table S3).

DISCUSSION

Principal findings

The main findings of this study were: (1) strong evidence to indicate that a higher proportion of MPs had poor mental health than among the general population, than among the defined occupational and socio-demographic comparator groups (EN, CM, AM, HIG). The primary study hypothesis was therefore confirmed. (2) Most MPs were not aware of Parliamentary mental health and support services. (3) Most MPs were not willing to discuss their mental health with party whips, and only a small majority would be happy to discuss mental health issues with other MPs. (4) Having employment outside Parliament, in addition to the role of MP, is not linked with increased risk for mental ill health.

The Parliamentary Health and Wellbeing Service is the occupational health service provided since 2013 inside the House of Commons. It aims to support all staff and MPs in developing a healthy and safe working environment, and encourages MPs to adopt better attitudes and behaviour towards their own physical health and mental health.[25] Despite the service being in place for almost four years, the Parliamentary Health and Wellbeing Service had reported low numbers of MPs requesting support. This study confirms this reluctance to seek help in finding that a majority of MPs are unaware of the service or how to access it. Reasons for this might be insufficient advertising of the support options offered and location of the services, as well as anticipated stigma and discrimination among MPs.[26]

Strengths and weaknesses of the study

For peer review only - http://bmjopen.bmj.com/site/about/guidelines.xhtml

The study has several limitations and potential biases. First, the response rate was relatively low (22.4%). Given the intense work loads of MPs, this may have been partly due to the additional workload of completing the survey, even though the median time to complete survey was only 4 minutes. Notably, a possible fear of being identified, of stigmatisation, and of the potential reputational damage associated with adverse media coverage may have influenced the response rate. We tried to reduce these biases by promoting the survey in Parliament, by sending several reminders, and by stressing the brevity, as well as the anonymity of the survey. Generally, MPs are a difficult survey population to engage, which has also been confirmed in a 2008 internal UK Parliament survey, where only 14.5% (94 MPs) responded.[27]

Secondly, it is also possible that MPs who responded to the online survey may have increased stress or mental ill health and that therefore a greater number of them were willing to complete the survey. A potential self-selection bias may therefore be present in the UKPMH sample. However, there is also a potential risk of under-reporting from people who might be reluctant to take part in the study, because they are affected by mental health problems, or because of the stigma associated with the topic. Prior experiences of, or fears of stalking and harassment, which might result from their disclosure, may decrease the willingness in MPs to participate in the survey.[28]

Respondents tended to be younger in relation to the age distribution of all MPs (18% of the UKPMH sample vs. 16% of total MP population were below 40 years old), and more likely to be female (36% female of the UKPMH sample vs 30% of total MPs population were female) in relation to the gender distribution of the total number of MPs and had a university degree (81% of the UKPMH sample vs 76% of total MP population). We did not assess marital or cohabitation status, as this would have increased the risk of identifiability of MPs, and this may have therefore also adversely affected the response rate.

Thirdly, comparing MPs to other occupational and socio-demographic groups within a population presents challenges. We considered comparing the UKPMH sample to the UK Health and Safety

For peer review only - http://bmjopen.bmj.com/site/about/guidelines.xhtml

Executive's Labour Force Survey (LFS), which provides annual data on rates of mental disorder by occupation.[29] However, the LFS relies on random household sampling is poorly suited to extrapolating meaningful data for a relatively small group 650 UK MPs. Published LFS data lacks sufficient granularity to be able to analyse the prevalence of mental disorders at an occupation-specific level, which for politicians would be 'elected officers and representatives'.[30] Given the unique features of political careers, including the diverse backgrounds from which politicians may be drawn, specific data relating to these generic occupational groupings are unlikely to be fully helpful in understanding why there is a higher burden of mental ill health. In this sample we found that having employment outside Parliament, and in addition to the role of MP, does not seem to constitute an increased risk for mental ill health. However, we regard this outcome with caution as this study may be underpowered to test for this specific variable, as most participants (81%) did not have employment outside Parliament.

Comparison of results with earlier studies

When examining UK parliamentary working hours reform, research found high levels of physical and emotional stress as a result of various aspects of political life such as additional work roles, extensive travel and job insecurity.[31] A longitudinal study in new UK MPs highlighted increased levels of stress post-election.[32] In 2008 the UK Parliament also conducted its own informal survey regarding experience and perceptions of mental illness, which concluded that one in five MPs had a personal experience of a mental health problem, and one in three felt stigma was a barrier to openness about mental health, yet no data on CMD were collected.[27] Given that work characteristics promoting stress are associated with mental disorders,[33, 34] it may be reasonable to assume that rates of CMD would be high in parliamentarians. However, no rigorous assessment has previously been conducted to investigate this issue.

Selected studies have investigated mental health in politicians, and although they have drawn on biographical evidence, their findings are in line with the results of this study. One study rated 46

For peer review only - http://bmjopen.bmj.com/site/about/guidelines.xhtml

statesmen and national leaders' biographies for psychopathology, and found increased rates for lifetime psychopathology, episodes of mental ill health, with only 15.2% of politicians showing no psychopathology at all.[35] A review of biographical sources looking at mental disorders in U.S. Presidents between 1776 and 1974, found that eighteen (49%) presidents met criteria indicative of psychiatric disorders.[36]

A cross-national study in the UK, Australia, New Zealand and Norway found that a higher proportion of MPs than the general public experience stalking, harassment and intrusive or aggressive behaviours.[28] They found that in the UK, 81% of MPs had experienced intrusive or aggressive behaviours, 18% been subject to attack/attempted attack, and 53% stalked or harassed. These intimidating experiences both have a negative impact on MPs' mental health and are likely to reinforce stigma and non-disclosure.[37]

This is the first study of assessment of mental health in members of Parliament of the UK House of Commons using structured, validated scales. These findings indicate that MPs are more likely to experience probable mental ill health and symptoms indicative of mental distress compared to the general population, and compared with similar occupational and professional groups. In addition, most MPs are not aware of mental health support offered by the Parliamentary Health and Wellbeing service, or willing to disclose to their whips or other MPs. This leaves MPs who have experience of mental ill health facing considerable difficulties without knowing how to access help.

Interpretation of the results

A number of studies have examined media and public reactions to politicians' actual or perceived mental health problems.[14-16] In an ever more hostile media environment, poor mental health can be regarded as a factor limiting politicians in their capacities. Stigma against people with mental disorders is prevalent in all countries and all sectors of society. It was not until 2013 that the UK passed the Mental Health (Discrimination) (No 2) Act 2013, which removed discriminatory provisions

For peer review only

permitting Members of Parliament (MPs) with mental health problems to be disqualified under certain circumstances.[38] Subsequent to the Act, there have been more disclosures from politicians about personal mental health problems. However, given that the results of this study showed that only 48% of surveyed MPs felt able to talk to their party whips, and only about half (52%) felt able to talk to another MP about their mental health, stigma and self-stigma about mental health appears to remain a powerful barrier to seeking help and support among Members of the UK House of Commons.

The power of disclosure as a catalyst for overcoming stigma has been demonstrated in 1998 when Kjell Magne Bondevik, then Prime Minister of Norway, spoke publicly about his experience of depression. His disclosure was empathetically received by the media and by the public.[39]

In 2012, during a House of Commons debate on mental health, four MPs disclosed their own mental health experiences. This eventually paved the way to providing MPs with access to mental health services in Westminster. Consequently, the Parliamentary Health and Wellbeing Service was created in 2013 and operates a mental health referral service as well as providing general medical advice, support and guidance to MPs and other staff working at Parliament. The service is nurse-led and is supported by one occupational health doctor for 3 days each week. It does not offer the more comprehensive health service that is often provided by General Practice in the United Kingdom. Our findings show poor awareness amongst MPs of the Parliamentary Health and Wellbeing Service and how to access it. This may be related to the restricted times that the service operates, or that the service is not located on the main Parliamentary Estate. These findings support the need for an increased mental health support for MPs and raising awareness about the Parliamentary Health and Wellbeing Service. They also support the need to for mental health stigma and self-stigma reduction amongst MPs.

Implications for future research

For peer review only - http://bmjopen.bmj.com/site/about/guidelines.xhtml

This is an initial study into the mental health of MPs, and further work is needed to assess the key issues identified, and to assess trends in the mental health of MPs over time. Our findings are only a starting point, but they reveal MPs' mental health problems and the need to properly assess them. A more granular assessment of mental health problems, including rates and consequences of alcohol and substance use-related problems, as well as cognitive impairment would be needed to provide a more in-depth picture. In terms of prevention, a better understanding of the causes for mental health problems and specific risk factors in MPs such as (cyber) bullying, harassment or stalking would be informative, and investigating effective mechanisms and strategies for prevention and increasing resilience. There is a need for better promotion of mental health support, such as the Parliamentary Health and Wellbeing Service, and for additional information and support for MPs in accessing the full range of mental health care. Due to their working routine and hours, MPs spend a majority of their working time far from the support provided by the NHS services in their own constituencies. In addition to their high-performance work life, this adds to the increased stress on MPs' mental health. It is also why strengthening the Parliamentary Health and Wellbeing Service could offer a specifically relevant support function. Research is also needed on mental health of other parliamentary staff, to identify their needs, and to evaluate their awareness of, and access to, the Parliamentary Health and Wellbeing Service and other relevant services.

CONCLUSION AND POLICY IMPLICATIONS

MPs have a vital role to play in the UK democracy: in making and scrutinising the legislation that governs the country, as well as in representing the interests of their constituents and the nation. This study has found the people in these important roles experience significantly higher levels of mental ill health when compared to the general population, and when compared to other senior executive and managerial groups. Most MPs do not feel that they have adequate mental health support, and they lack knowledge of how to access the mental health services that are available to them. Most MPs are

For peer review only

For peer review only - http://bmjopen.bmj.com/site/about/guidelines.xhtml

not able to discuss their mental health problems with their whips or other MPs. These findings indicate that better support is required both to prevent mental health problems among MPs and to ensure rapid and effective care when needed, to support MPs in their vital work for the people they serve.

Acknowledgments: We would like to thank all Members of Parliament who took part in this study. In addition, we would like to thank Elaine Bryce (member of Dr Daniel Poulter's Parliamentary office), and the staff of the Parliamentary Health and Wellbeing Service for their support in this study.

Funding: This research received no specific grant from any funding agency in the public, commercial or not-for-profit sectors.

Competing interests:

NV acknowledges funding from the Economic and Social Research Council (ESRC) and National Institute for Health Research (NIHR) Collaboration for Leadership in Applied Health Research and Care South London at King's College London NHS Foundation Trust.

GT is supported by the National Institute for Health Research (NIHR) Collaboration for Leadership in Applied Health Research and Care South London at King's College London NHS Foundation Trust. The views expressed are those of the author(s) and not necessarily those of the NHS, the NIHR or the Department of Health. GT acknowledges financial support from the Department of Health via the National Institute for Health Research (NIHR) Biomedical Research Centre and Dementia Unit awarded to South London and Maudsley NHS Foundation Trust in partnership with King's College London and King's College Hospital NHS Foundation Trust. GT is supported by the European Union Seventh Framework Programme (FP7/2007-2013) Emerald project. GT also receives support from the National Institute of Mental Health of the National Institutes of Health under award number R01MH100470 (Cobalt study). GT is also supported by the UK Medical Research Council in relation the Emilia (MR/S001255/1) and Indigo Partnership (MR/R023697/1) awards.

For peer review only

IB is supported by the NIHR Biomedical Research Centre at South London and Maudsley NHS Foundation Trust and by the NIHR Collaboration for Leadership in Applied Health Research.

JD has a Clinician Scientist Fellowship, funded by the Health Foundation working with the Academy of Medical Sciences

All authors have completed the ICMJE uniform disclosure form at www.icmje.org/coi_disclosure.pdf and declare: no support from any organisation for the submitted work; no financial relationships with any organisations that might have an interest in the submitted work in the previous three years; DP is currently MP of the 57th UK Parliament and was member of the 56th UK Parliament; no other relationships or activities that could appear to have influenced the submitted work.

Copyright/license for publication: The Corresponding Author has the right to grant on behalf of all authors and does grant on behalf of all authors, a worldwide licence to the Publishers and its licensees in perpetuity, in all forms, formats and media (whether known now or created in the future), to i) publish, reproduce, distribute, display and store the Contribution, ii) translate the Contribution into other languages, create adaptations, reprints, include within collections and create summaries, extracts and/or, abstracts of the Contribution, iii) create any other derivative work(s) based on the Contribution, iv) to exploit all subsidiary rights in the Contribution, v) the inclusion of electronic links from the Contribution to third party material where-ever it may be located; and, vi) licence any third party to do any or all of the above."

Ethical approval: Ethics approval for the study was obtained in September 2016 from King's College London Ethics Committee (reference number: HR-16/17-3118).

Data sharing: No additional data available. The Health Survey for England 2014 can be accessed at: https://digital.nhs.uk/data-and-information/publications/statistical/health-survey-for-england/health-survey-for-england-2014. Due to the sensibility of the data, and in order to ensure full

For peer review only

anonymity, confidentiality and data protection for the participants, the full survey data cannot be made accessible to the public.

Transparency declaration: The corresponding author affirms that the manuscript is an honest, accurate, and transparent account of the study being reported; that no important aspects of the study have been omitted; and that any discrepancies from the study as planned have been explained.

Contributors: DP and GT conceived the original idea for the study, which was then discussed with NV. NV coordinated the study. All authors contributed to the design of the study. NV and FD conducted the literature review. DP and NV collected the data. IB conducted design and analysis of the data. JD supported the design of the data analysis, and contributed throughout the design and writing up of the study. NV led the writing of the manuscript, and all authors contributed and critically revised it. All authors have given their approval for the publication of this manuscript and agree to be accountable for all aspects of the work to ensure that the questions related to the accuracy or integrity of any part of the work are appropriately investigated and resolved.

For peer review only

For peer review only - http://bmjopen.bmj.com/site/about/guidelines.xhtml

References

1. Davidson J. Downing Street Blues: A History of Depression and Other Mental Afflictions in British Prime Ministers: McFarland; 2010.

2. Fieve R. The case of Dominique Straus-Kahn and Mental Disorder. International Clinical Psychopharmacology. 2012;28:e14-e5.

3. Freedman L. Mental states and political decisions: Commentary on... Psychiatry and politicians. The Psychiatrist. 2011;35(4):148-50.

4. Peters U. Daniel Paul Schreber, the illness of the Senate president. Fortschritte der Neurologie-Psychiatrie. 1995;63(12):469-79.

5. Owen D. In sickness and in power: illnesses in heads of government during the last 100 years,. Methuen Publishing, London; 2008.

6. Owen LD. Hubris and nemesis in heads of government. Journal of the Royal Society of Medicine. 2006;99(11):548-51.

7. Russell G. Psychiatry and politicians: the ‘hubris syndrome’. The Psychiatrist. 2011;35(4):140-5.

8. Sidwell B. Gaius Caligula's Mental Illness. Classical world. 2010;103(2):183-206.

9. Vatz RE. Rhetoric and psychiatry: A Szaszian perspective on a political case study. Current Psychology. 2006;25(3):173-81.

10. James DV, Mullen PE, Meloy JR, Pathé MT, Farnham FR, Preston L, et al. The role of mental disorder in attacks on European politicians 1990–2004. Acta Psychiatrica Scandinavica. 2007;116(5):334-44.

11. James DV, Sukhwal S, Farnham FR, Evans J, Barrie C, Taylor A, et al. Harassment and stalking of Members of the United Kingdom Parliament: associations and consequences. The Journal of Forensic Psychiatry & Psychology. 2016;27(3):309-30.

12. Gersons BPR, Nijdam MJ. Supporting leaders under threat and their protection. In: Parkes CM, editor. Responses to Terrorism: Can psychosocial approaches break the cycle of violence? New York: Routledge; 2014. p. 181-444.

13. Every-Palmer S, Barry-Walsh J, Pathé M. Harassment, stalking, threats and attacks targeting New Zealand politicians: A mental health issue. Australian & New Zealand Journal of Psychiatry. 2015;49(7):634-41.

14. Altheide DL. Mental illness and the news: The Eagleton story. Sociology & Social Research. 1977.

15. Dukakis MS. Campaigns and disability: When an incumbent president questions his potential successor's mental health status during the campaign. Politics & Life Sciences. 2015;33(2):88-92.

For peer review only

16. MacDonald A, Majumder RK. On the resolution and tolerance of cognitive inconsistency in another naturally occurring event: Attitudes and beliefs following the Senator Eagleton incident. Journal of Applied Social Psychology. 1973;3(2):132-43.

17. Tolor A. Opinions about mental illness and political ideology. American Journal of Psychiatry. 1973;130(11):1269-72.

18. Wykes T, Craig T. Can our politicians help to reduce stigma and discrimination? J Ment Health. 2013;22(3):203-6.

19. Equator Network. The Strengthening the Reporting of Observational Studies in Epidemiology (STROBE) Statement: guidelines for reporting observational studies [Available from: http://www.equator-network.org/reporting-guidelines/strobe/.

20. Craig R, Fuller E, Mindell J. Health survey for England 2014. 2015.

21. Goldberg DP, Blackwell B. Psychiatric illness in general practice: a detailed study using a new method of case identification. Br med J. 1970;2(5707):439-43.

22. Goldberg DP, Hillier VF. A scaled version of the General Health Questionnaire. Psychological medicine. 1979;9(1):139-45.

23. Goldberg DP, Gater R, Sartorius N, Ustun TB, Piccinelli M, Gureje O, et al. The validity of two versions of the GHQ in the WHO study of mental illness in general health care. Psychol Med. 1997;27(1):191-7.

24. Hofler M, Pfister H, Lieb R, Wittchen HU. The use of weights to account for non-response and drop-out. Soc Psychiatry Psychiatr Epidemiol. 2005;40(4):291-9.

25. UK Parliament. New support announced for MPs with mental health problems 12 February 2013 [Available from: http://www.parliament.uk/business/committees/committees-a-z/other-committees/members-estimate/news/new-support-announced-for-mps-with-mental-health-problems/.

26. Thornicroft G, Mehta N, Clement S, Evans-Lacko S, Doherty M, Rose D, et al. Evidence for effective interventions to reduce mental-health-related stigma and discrimination. The Lancet. 2016;387(10023):1123-32.

27. All-Party Parliamentary Group on Mental Health. Mental Health in Parliament: Report by the All-Party Parliamentary Group on Mental Health. 2008.

28. James DV, Farnham FR, Sukhwal S, Jones K, Carlisle J, Henley S. Aggressive/intrusive behaviours, harassment and stalking of members of the United Kingdom parliament: a prevalence study and cross-national comparison. The Journal of Forensic Psychiatry & Psychology. 2016;27(2):177-97.

29. Health and Safety Executive. Labour Force Survey data for year 2013/14-2015/16 2016.

30. Office for National Statistics. Standard Occupational Classification 2010

31. Weinberg A, Cooper CL. Workload, stress and family life in British Members of Parliament and the psychological impact of reforms to their working hours. Stress and Health. 1999;15(2):79-87.

For peer review only

32. Weinberg A, Cooper C. Stress among national politicians elected to Parliament for the first time. Stress and Health. 2003;19(2):111-7.

33. Wilhelm K, Kovess V, Rios-Seidel C, Finch A. Work and mental health. Social psychiatry and psychiatric epidemiology. 2004;39(11):866-73.

34. Wang J, Lesage A, Schmitz N, Drapeau A. The relationship between work stress and mental disorders in men and women: findings from a population-based study. Journal of Epidemiology & Community Health. 2008;62(1):42-7.

35. Post F. Creativity and Psychopathology a Study of 291 World-Famous Men. British Journal of Psychiatry. 1994;165(1):22-34.

36. Davidson JR, Connor KM, Swartz M. Mental illness in U.S. Presidents between 1776 and 1974: a review of biographical sources. J Nerv Ment Dis. 2006;194(1):47-51.

37. Kuehner C, Gass P, Dressing H. Increased risk of mental disorders among lifetime victims of stalking--findings from a community study. European psychiatry : the journal of the Association of European Psychiatrists. 2007;22(3):142-5.

38. Wykes T, Craig T. Can our politicians help to reduce stigma and discrimination? : Taylor & Francis; 2013.

39. Brustad S. Foreword by the Minister of Health and Care Services of Norway. In: Knapp M, McDaid D, Mossialos E, editors. Mental health policy and practice across Europe. Maidenhead: McGraw-Hill Education (UK); 2006. p. xxiv-xxv.

For peer review only

For peer review only - http://bmjopen.bmj.com/site/about/guidelines.xhtml

List of figures and legends

Figure 1:
Age-Sex standardised prevalence estimates and 95% Confidence Intervals of UKPMH and of specific population groups of HSE 2014 for the three different categories of Common Mental Disorders (CMD).

Key: MP: Member of Parliament Sample; EN: English Population (HSE 2014); CM: Corporate Managers (HSE 2014); AM: All managers (HSE 2014); HIG: High-income group (HSE 2014).

Figure 2:
Access to the mental health (MH) support of the Parliamentary Health and Wellbeing Service

NB: All p-values <0.001.

Figure 3:
Awareness of the mental health (MH) support of the Parliamentary Health and Wellbeing Service

NB: All p-values <0.001

Figure 4:
Willingness to talk to party whips

NB: All p-values <0.001

Figure 5:
Willingness to talk to other MPs

NB: All p-values <0.001

For peer review only - http://bmjopen.bmj.com/site/about/guidelines.xhtml

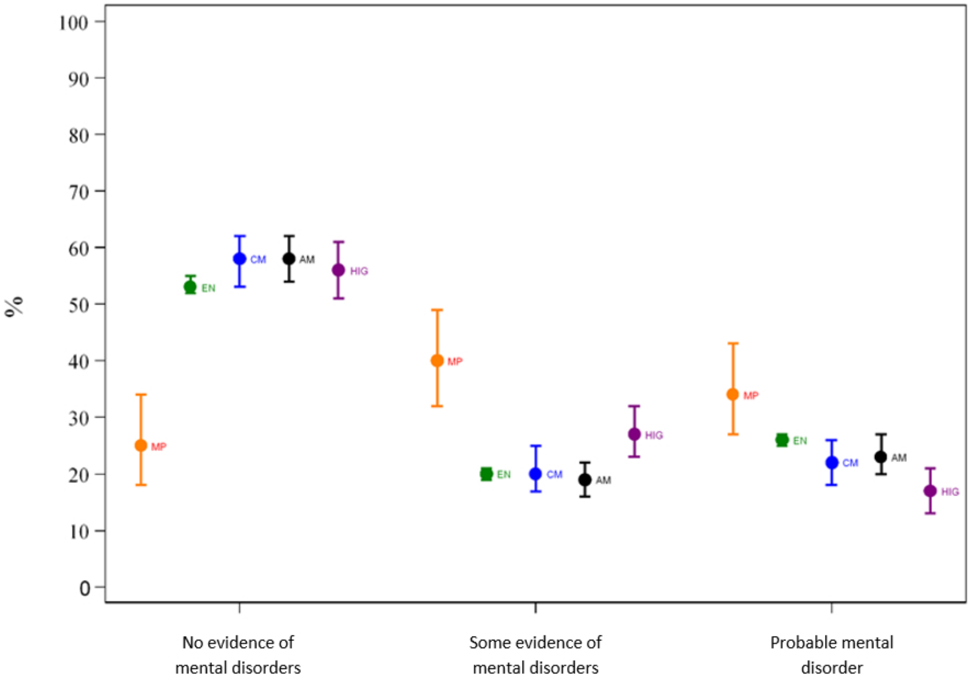

Figure 1: Age-Sex standardised prevalence estimates and 95% Confidence Intervals of UKPMH and of specific population groups of HSE 2014 for the three different categories of Common Mental Disorders (CMD).

Key: MP: Member of Parliament Sample; EN: English Population (HSE 2014); CM: Corporate Managers (HSE 2014); AM: All managers (HSE 2014); HIG: High-income group (HSE 2014).

192x141mm (120 x 120 DPI)

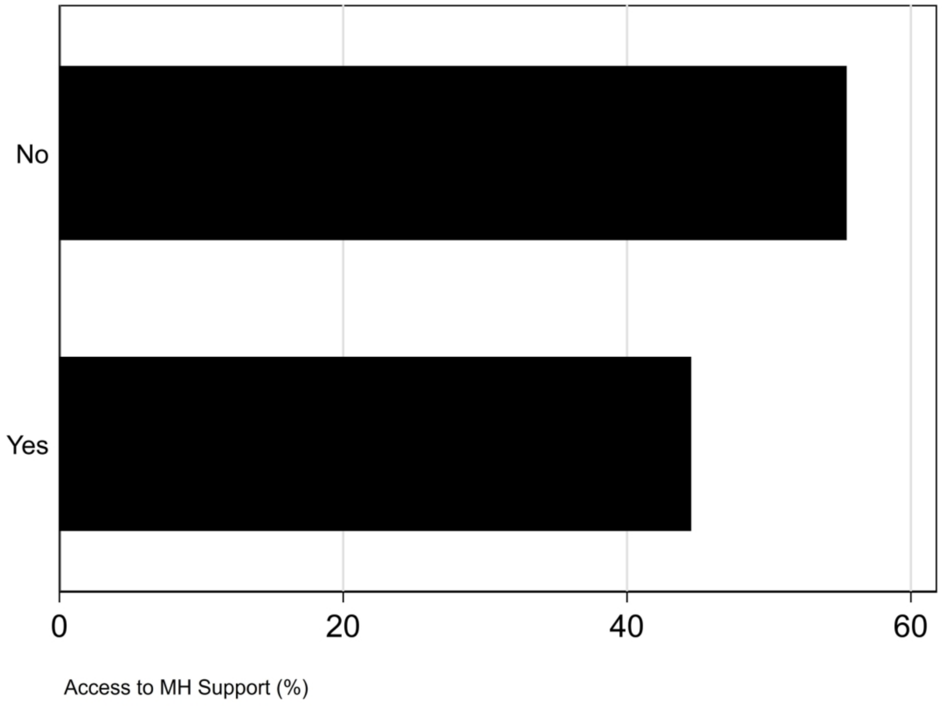

Figure 2: Access to the mental health (MH) support of the Parliamentary Health and Wellbeing Service

NB: All p-values <0.001.

169x127mm (300 x 300 DPI)

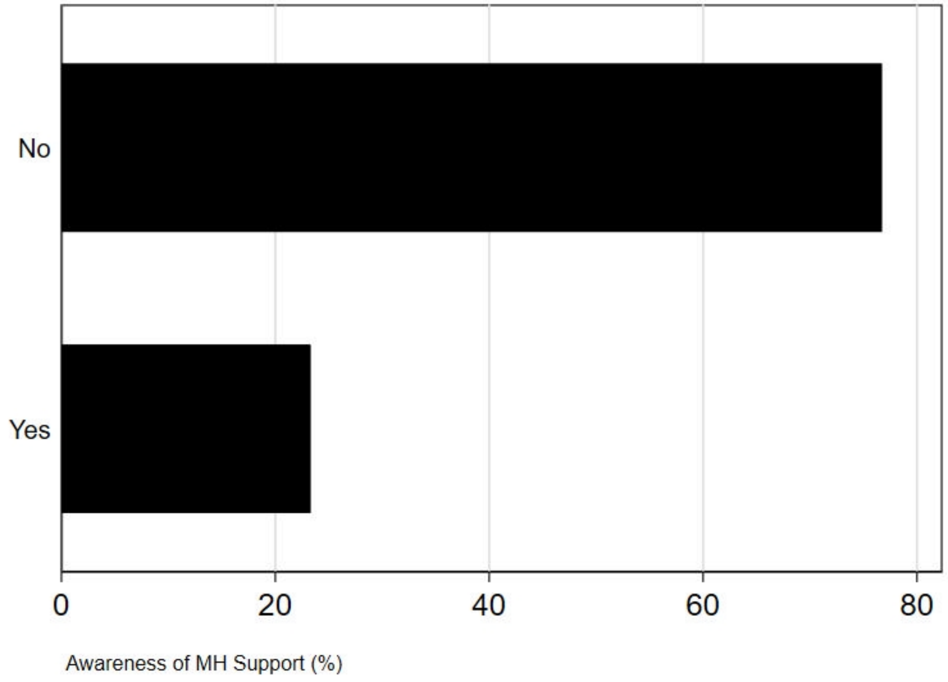

Awareness of the mental health (MH) support of the Parliamentary Health and Wellbeing Service

NB: All p-values <0.001

66x48mm (300 x 300 DPI)

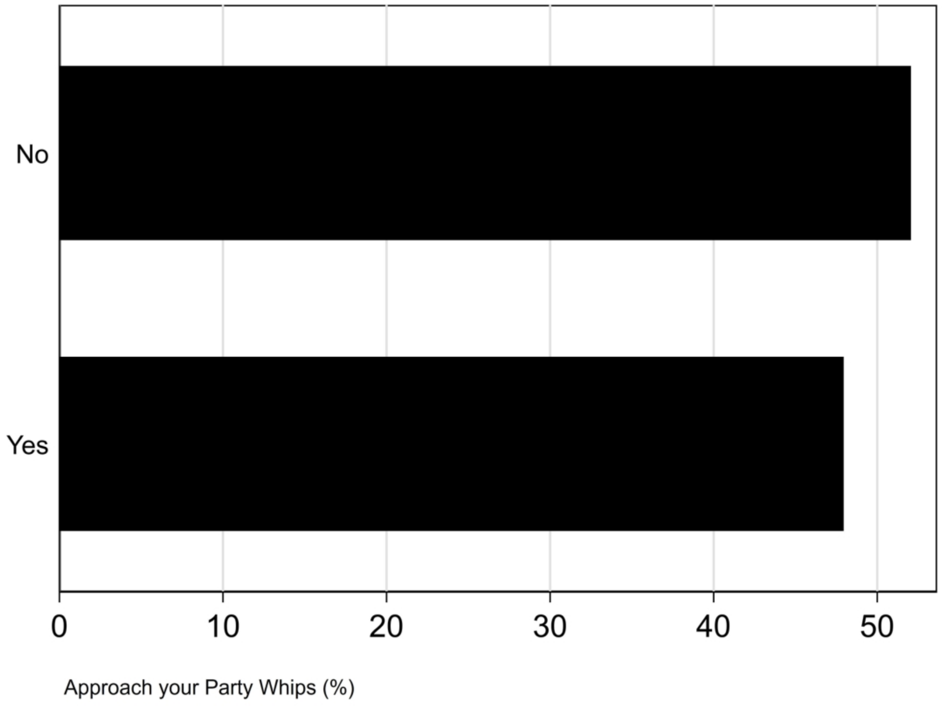

Figure 4: Willingness to talk to party whips

NB: All p-values <0.001.

169x127mm (300 x 300 DPI)

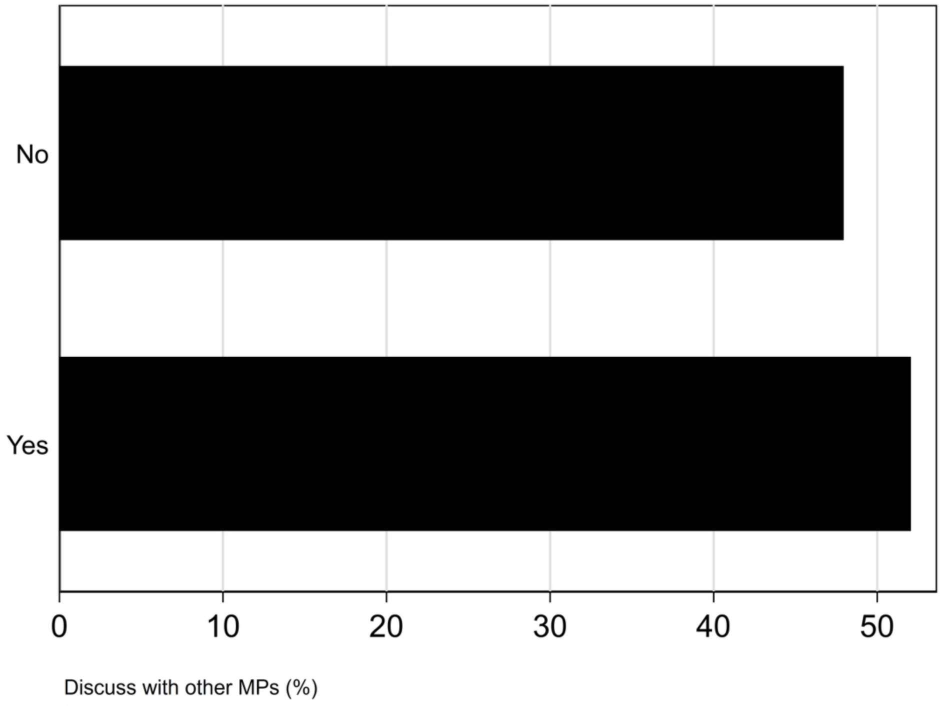

Figure 5: Willingness to talk to other MPs

NB: All p-values <0.001.

169x127mm (300 x 300 DPI)

Supplementary File (Online)

1. FULL LIST OF QUESTIONS

UKMPH Survey 2016: list of demographic questions

1. What age group are you?
    - Age 21 to 30
    - Age 31 to 40
    - Age 41 to 50
    - Age 51 to 60
    - Age 61 to 70
    - Age 70 +

2. How long have you been a Westminster MP?
    - Less than 5 years
    - 5 to 10 years
    - 11 to 15 years
    - 16 to 20 years
    - 21 to 25 years
    - More than 25 years

3. What is your highest level of educational attainment?
    - GCSE / O Level
    - A Level / Scottish Higher
    - Vocational Qualifications (BTEC, NVQ, HNC etc)
    - Undergraduate Degree (BA, BSc, or equivalent)
    - Post Graduate (MA, MSC, or equivalent)
    - Doctorate (PhD or equivalent)

4. What is your gender?
    - Male
    - Female

5. Do you have a job / role outside of Parliament?
    - Yes - Paid
    - Yes - Unpaid
    - No

For peer review only

UKMPH Survey 2016: List of questions on inhouse mental health services

6. Do you know how to access Mental Health Support through the Parliamentary Health and Wellbeing Service?
    - Yes
    - No

7. Does the Parliamentary Health and Wellbeing Service currently offer enough support to meet your mental health needs?
    - Yes
    - Somewhat
    - No

8. Would you be happy to approach your Party Whip's office if you were experiencing mental health problems?
    - Yes
    - No

9. Would you be happy to discuss with other MPs if you were experiencing mental health problems?
    - Yes
    - No

For peer review only

2. TABLE S1

Table S1: Descriptive characteristics of the 12 item GHQ (GHQ-12) and the four different predetermined HSE 2014 occupational and sociodemographic comparator groups (EN, CM, AM, HIG) - for Males

	n	WP	n	WP	n	WP	n	WP	n	WP
		**MP**		**EN**		**CM**		**AM**		**HIG**
		**95% CI**		**95% CI**		**95% CI**		**95% CI**		**95% CI**
**Age**										
21-30	2	0.00<br>0.00 to 0.01	515	0.22<br>0.20 to 0.23	26	0.19<br>0.13 to 0.27	35	0.17<br>0.12 to 0.23	32	0.18<br>0.13 to 0.25
31-40	15	0.10<br>0.06 to 0.16	558	0.17<br>0.16 to 0.19	28	0.12<br>0.08 to 0.17	46	0.14<br>0.10 to 0.18	59	0.23<br>0.18 to 0.29
41-50	27	0.31<br>0.22 to 0.42	702	0.19<br>0.18 to 0.20	56	0.22<br>0.17 to 0.28	74	0.19<br>0.16 to 0.24	57	0.19<br>0.15 to 0.25
51-60	31	0.49<br>0.38 to 0.60	606	0.16<br>0.15 to 0.17	38	0.17<br>0.13 to 0.23	66	0.20<br>0.15 to 0.24	43	0.14<br>0.10 to 0.18
61-70	15	0.10<br>0.06 to 0.17	632	0.14<br>0.13 to 0.15	51	0.17<br>0.13 to 0.22	78	0.18<br>0.14 to 0.22	45	0.13<br>0.10 to 0.18
70 +	2	0.00<br>0.00 to 0.01	565	0.12<br>0.11 to 0.13	37	0.12<br>0.09 to 0.16	60	0.13<br>0.10 to 0.16	46	0.13<br>0.09 to 0.17
**Educational attainment**										
NVQ4/NVQ5/ Degree	0	0.79<br>0.69 to 0.87	931	0.28<br>0.26 to 0.30	98	0.42<br>0.35 to 0.49	122	0.34<br>0.29 to 0.40	214	0.77<br>0.72 to 0.82
Higher ed below degree	72	0.09<br>0.04 to 0.17	524	0.13<br>0.12 to 0.14	29	0.13<br>0.09 to 0.19	46	0.13<br>0.09 to 0.17	29	0.08<br>0.06 to 0.12
NVQ3/GCE A Level	8	0.03<br>0.01 to 0.08	504	0.16<br>0.15 to 0.17	36	0.16<br>0.11 to 0.22	58	0.18<br>0.14 to 0.23	20	0.08<br>0.05 to 0.13
NVQ2/GCE O Level	3	0.10<br>0.05 to 0.19	631	0.18<br>0.17 to 0.20	41	0.17<br>0.13 to 0.23	66	0.18<br>0.14 to 0.23	13	0.04<br>0.02 to 0.07
NVQ1/CSE other grade	9	N/A	190	0.05<br>0.04 to 0.06	5	0.03<br>0.01 to 0.07	9	0.03<br>0.01 to 0.06	2	0.01<br>0.00 to 0.02
Foreign/other	0	N/A	9	0.00<br>0.00 to 0.01	0	0.09<br>0.06 to 0.14	1	0.01<br>0.00 to 0.04	0	0.01<br>0.00 to 0.03
No qualification	0	N/A	768	0.20<br>0.19 to 0.21	26	N/A	56	0.14<br>0.10 to 0.18	4	N/A
**GHQ -12**										
**Item 1: Have you recently been able to concentrate on whatever you're doing?**										
Better than usual	2	0.02<br>0.00 to 0.10	100	0.04<br>0.03 to 0.05	5	0.03<br>0.01 to 0.07	9	0.03<br>0.01 to 0.06	7	0.03<br>0.01 to 0.06
Same as usual	61	0.68<br>0.56 to 0.77	2746	0.87<br>0.85 to 0.88	193	0.89<br>0.83 to 0.94	290	0.90<br>0.85 to 0.93	239	0.92<br>0.87 to 0.95
Less than usual	26	0.26<br>0.18 to 0.37	284	0.08<br>0.07 to 0.09	12	0.06<br>0.03 to 0.11	18	0.06<br>0.04 to 0.10	14	0.05<br>0.03 to 0.09
Much less than usual	3	0.04<br>0.01 to 0.12	40	0.01<br>0.01 to 0.02	2	0.02<br>0.00 to 0.08	3	0.02<br>0.00 to 0.06	0	N/A
**Item 2: Have you recently lost much sleep over worry?**										
Not at all	18	0.19<br>0.12 to 0.30	1211	0.38<br>0.36 to 0.40	81	0.39<br>0.32 to 0.46	128	0.39<br>0.34 to 0.45	92	0.33<br>0.28 to 0.40
No more than usual	42	0.47<br>0.36 to 0.58	1519	0.48<br>0.46 to 0.50	114	0.53<br>0.45 to 0.60	166	0.52<br>0.46 to 0.58	136	0.56<br>0.49 to 0.62
Rather more than usual	26	0.27<br>0.19 to 0.38	352	0.11<br>0.10 to 0.12	15	0.07<br>0.04 to 0.12	23	0.08<br>0.05 to 0.11	30	0.11<br>0.07 to 0.15
Much more than usual	6	0.06<br>0.03 to 0.14	89	0.03<br>0.02 to 0.03	2	0.01<br>0.00 to 0.04	3	0.01<br>0.00 to 0.04	2	0.01<br>0.00 to 0.02
**Item 3: Have you recently felt you were playing a useful part in things?**										

For peer review only

More so than usual	14	0.15<br>0.09 to 0.25	291	0.10<br>0.09 to 0.11	26	0.13<br>0.09 to 0.19	35	0.11<br>0.08 to 0.16	27	0.10<br>0.07 to 0.15
Same as usual	47	0.49<br>0.38 to 0.60	2533	0.80<br>0.79 to 0.82	171	0.78<br>0.71 to 0.84	257	0.78<br>0.73 to 0.83	215	0.82<br>0.77 to 0.87
Less useful than usual	28	0.31<br>0.22 to 0.42	274	0.08<br>0.07 to 0.09	15	0.09<br>0.05 to 0.15	27	0.10<br>0.07 to 0.15	16	0.07<br>0.04 to 0.11
Much less useful	3	0.04<br>0.01 to 0.13	66	0.02<br>0.01 to 0.03	0	N/A	1	0.00<br>0.00 to 0.02	2	0.01<br>0.00 to 0.03
**Item 4: Have you recently felt capable of making decisions about things?**										
More so than usual	5	0.05<br>0.02 to 0.13	231	0.08<br>0.07 to 0.10	13	0.08<br>0.04 to 0.14	20	0.07<br>0.04 to 0.11	20	0.07<br>0.05 to 0.12
Same as usual	77	0.86<br>0.76 to 0.92	2745	0.86<br>0.84 to 0.87	193	0.88<br>0.81 to 0.93	290	0.89<br>0.84 to 0.92	232	0.89<br>0.84 to 0.92
Less so than usual	10	0.09<br>0.05 to 0.18	171	0.05<br>0.04 to 0.06	6	0.04<br>0.02 to 0.09	10	0.04<br>0.02 to 0.08	8	0.04<br>0.02 to 0.08
Much less capable	0	N/A	23	0.01<br>0.00 to 0.01	0	N/A	0	N/A	0	N/A
**Item 5: Have you felt under constant strain recently?**										
Not at all	8	0.08<br>0.04 to 0.17	837	0.27<br>0.25 to 0.29	68	0.32<br>0.25 to 0.39	106	0.32<br>0.27 to 0.38	64	0.23<br>0.18 to 0.29
No more than usual	42	0.43<br>0.33 to 0.54	1773	0.56<br>0.54 to 0.58	114	0.53<br>0.45 to 0.60	168	0.52<br>0.46 to 0.58	143	0.54<br>0.47 to 0.60
Rather more than usual	33	0.38<br>0.28 to 0.50	466	0.14<br>0.13 to 0.16	27	0.15<br>0.10 to 0.21	42	0.15<br>0.11 to 0.20	49	0.21<br>0.16 to 0.28
Much more than usual	9	0.10<br>0.05 to 0.19	92	0.03<br>0.02 to 0.03	3	0.01<br>0.00 to 0.03	4	0.01<br>0.00 to 0.02	4	0.02<br>0.01 to 0.06
**Item 6: Have you recently felt you couldn't overcome your difficulties?**										
Not at all	31	0.31<br>0.22 to 0.42	1191	0.39<br>0.37 to 0.40	88	0.41<br>0.34 to 0.49	138	0.43<br>0.37 to 0.49	96	0.34<br>0.29 to 0.41
No more than usual	45	0.52 0.41 to 0.63	1680	0.52<br>0.51 to 0.54	107	0.51<br>0.43 to 0.58	155	0.49<br>0.43 to 0.54	148	0.59<br>0.52 to 0.65
Rather more than usual	14	0.15<br>0.09 to 0.24	241	0.07<br>0.06 to 0.08	15	0.07<br>0.04 to 0.12	24	0.08<br>0.05 to 0.12	16	0.07<br>0.04 to 0.11
Much more than usual	2	0.02<br>0.01 to 0.09	55	0.02<br>0.01 to 0.02	2	0.01<br>0.00 to 0.06	3	0.01<br>0.00 to 0.04	0	N/A
**Item 7: Have you recently been able to enjoy your normal day to day activities?**										
More so than usual	1	0.01<br>0.00 to 0.05	158	0.06<br>0.05 to 0.07	15	0.09<br>0.05 to 0.15	23	0.08<br>0.05 to 0.13	12	0.04<br>0.02 to 0.07
Same as usual	58	0.61<br>0.50 to 0.71	2537	0.80<br>0.78 to 0.82	174	0.79<br>0.71 to 0.85	256	0.77<br>0.71 to 0.82	220	0.84<br>0.78 to 0.88
Less so than usual	26	0.31<br>0.22 to 0.42	382	0.12<br>0.10 to 0.13	18	0.10<br>0.06 to 0.17	33	0.12<br>0.08 to 0.17	27	0.12<br>0.08 to 0.18
Much less than usual	7	0.07<br>0.03 to 0.15	88	0.02<br>0.02 to 0.03	4	0.02<br>0.01 to 0.05	8	0.02<br>0.01 to 0.05	1	0.00<br>0.00 to 0.02
**Item 8: Have you recently been able to face up to your problems?**										
More so than usual	7	0.08<br>0.04 to 0.16	154	0.06<br>0.05 to 0.07	9	0.06<br>0.03 to 0.12	15	0.06<br>0.03 to 0.10	9	0.04<br>0.02 to 0.08
Same as usual	73	0.78<br>0.68 to 0.86	2746	0.87<br>0.86 to 0.88	191	0.90<br>0.84 to 0.94	287	0.90<br>0.86 to 0.93	235	0.91<br>0.86 to 0.95
Less able than usual	12	0.14<br>0.08 to 0.24	198	0.06<br>0.05 to 0.07	6	0.03<br>0.01 to 0.06	10	0.04<br>0.02 to 0.07	12	0.05<br>0.02 to 0.09
Much less able	0	N/A	29	0.01<br>0.01 to 0.01	1	0.01<br>0.00 to 0.06	1	0.01<br>0.00 to 0.04	0	N/A
**Item 9: Have you recently been feeling unhappy and depressed?**										
Not at all	29	0.31<br>0.21 to 0.42	1263	0.41<br>0.39 to 0.43	96	0.46<br>0.38 to 0.53	151	0.48<br>0.42 to 0.54	104	0.38<br>0.31 to 0.44
No more than usual	38	0.43<br>0.32 to 0.54	1420	0.45<br>0.43 to 0.47	90	0.43<br>0.36 to 0.51	130	0.41<br>0.35 to 0.47	129	0.54<br>0.47 to 0.60
Rather more than usual	25	0.26<br>0.18 to 0.37	366	0.12<br>0.10 to 0.13	20	0.11<br>0.07 to 0.17	28	0.10<br>0.07 to 0.14	23	0.09<br>0.06 to 0.13

For peer review only

Much more than usual	0	N/A	75	0.02 0.02 to 0.03	1	0.00 0.00 to 0.03	3	0.01 0.00 to 0.04	0	N/A
**Item 10: Have you recently been losing confidence in yourself?**										
**Not at all**	38	0.39 0.29 to 0.51	1510	0.49 0.47 to 0.51	113	0.53 0.46 to 0.61	176	0.55 0.49 to 0.61	132	0.48 0.42 to 0.55
No more than usual	39	0.43 0.33 to 0.54	1290	0.41 0.39 to 0.43	80	0.39 0.32 to 0.47	116	0.37 0.32 to 0.43	106	0.44 0.38 to 0.51
Rather more than usual	15	0.17 0.10 to 0.28	263	0.09 0.07 to 0.10	11	0.07 0.04 to 0.13	17	0.07 0.04 to 0.12	18	0.07 0.04 to 0.12
Much more than usual	0	N/A	58	0.02 0.01 to 0.02	1	0.00 0.00 to 0.03	2	0.01 0.00 to 0.03	0	N/A
**Item 11: Have you recently been thinking of yourself as a worthless person?**										
Not at all	54	0.57 0.45 to 0.67	2128	0.69 0.67 to 0.70	152	0.72 0.65 to 0.78	231	0.73 0.67 to 0.78	184	0.70 0.64 to 0.76
No more than usual	29	0.32 0.23 to 0.43	810	0.25 0.24 to 0.27	43	0.20 0.15 to 0.27	64	0.20 0.16 to 0.26	65	0.27 0.21 to 0.34
Rather more than usual	9	0.11 0.06 to 0.20	134	0.04 0.04 to 0.05	12	0.08 0.04 to 0.14	17	0.07 0.04 to 0.11	6	0.02 0.01 to 0.05
Much more than usual	0	N/A	53	0.02 0.01 to 0.02	0	N/A	1	0.00 0.00 to 0.03	1	0.00 0.00 to 0.03
**Item 12: Have you recently been feeling reasonably happy, all things considered?**										
More so than usual	5	0.06 0.02 to 0.14	310	0.11 0.10 to 0.12	18	0.11 0.07 to 0.17	26	0.10 0.06 to 0.14	29	0.12 0.08 to 0.18
About same as usual	67	0.71 0.60 to 0.80	2510	0.80 0.78 to 0.81	175	0.82 0.75 to 0.87	266	0.83 0.77 to 0.87	215	0.83 0.77 to 0.87
Less so than usual	20	0.23 0.15 to 0.33	243	0.07 0.06 to 0.08	13	0.06 0.03 to 0.10	18	0.06 0.04 to 0.09	11	0.05 0.02 to 0.08
Much less than usual	0	N/A	59	0.02 0.01 to 0.02	1	0.01 0.00 to 0.06	4	0.02 0.01 to 0.05	1	0.00 0.00 to 0.02
**Presence of probable mental ill health**										
No evidence of probable MIH	26	0.29 0.20 to 0.40	2009	0.56 0.55 to 0.58	143	0.58 0.51 to 0.65	220	0.58 0.52 to 0.64	168	0.58 0.52 to 0.65
Less than optimal MIH	39	0.41 0.30 to 0.52	665	0.19 0.17 to 0.20	44	0.22 0.16 to 0.28	61	0.19 0.15 to 0.24	69	0.27 0.21 to 0.33
MIH	27	0.30 0.21 to 0.41	904	0.25 0.23 to 0.26	49	0.20 0.15 to 0.27	78	0.23 0.18 to 0.28	45	0.15 0.11 to 0.20

Weighted proportion (WP) with the corresponding 95% Confidence Intervals (CI).

Key: MP: Member of Parliament Sample; EN: English Population (HSE 2014); CM: Corporate Managers (HSE 2014); AM: All managers (HSE 2014); HIG: high-income group (HSE 2014).

For peer review only - http://bmjopen.bmj.com/site/about/guidelines.xhtml

3. TABLE S2

Table S2: Descriptive characteristics of the 12 item GHQ (GHQ-12) and the four different predetermined HSE 2014 occupational and sociodemographic comparator groups (EN, CM, AM, HIG) - for Females

	n	WP	n	WP	n	WP	n	WP	n	WP
		**MP**		**EN**		**CM**		**AM**		**HIG**
		**95% CI**		**95% CI**		**95% CI**		**95% CI**		**95% CI**
**Age**										
21-30	2	0.00<br>0.00 to 0.01	681	0.20<br>0.19 to 0.22	21	0.13<br>0.08 to 0.21	36	0.13<br>0.09 to 0.19	26	0.21<br>0.14 to 0.29
31-40	8	0.08<br>0.04 to 0.17	784	0.17<br>0.16 to 0.19	45	0.16<br>0.12 to 0.21	72	0.17<br>0.14 to 0.21	55	0.32<br>0.25 to 0.40
41-50	17	0.32<br>0.20 to 0.46	845	0.18<br>0.17 to 0.19	66	0.24<br>0.19 to 0.29	83	0.20<br>0.16 to<br>0.24	30	0.18<br>0.12 to 0.25
51-60	21	0.54<br>0.39 to 0.68	726	0.16<br>0.15 to<br>0.17	51	0.21<br>0.16 to<br>0.28	80	0.21<br>0.17 to 0.26	21	0.12<br>0.08 to 0.18
61-70	5	0.06<br>0.02 to 0.14	681	0.13<br>0.12 to 0.14	44	0.15<br>0.11 to 0.19	71	0.16<br>0.13 to 0.20	20	0.11<br>0.07 to 0.17
70 +	1	0.00<br>0.00 to 0.01	722	0.15<br>0.14 to 0.16	32	0.11<br>0.08 to<br>0.15	55	0.12<br>0.10 to 0.16	11	0.06<br>0.03 to 0.11
**Educational attainment**										
NVQ4/NVQ5/Degree	0	N/A	1106	0.27<br>0.25 to<br>0.28	84	0.33<br>0.27 to 0.40	115	0.30<br>0.25 to 0.35	14<br>1	0.88<br>0.82 to 0.92
Higher ed below degree	47	0.05<br>0.01 to 0.17	483	0.10<br>0.09 to<br>0.11	39	0.15<br>0.11 to<br>0.20	58	0.14<br>0.11 to 0.18	5	0.03<br>0.01 to 0.06
NVQ3/GCE A Level	3	0.02<br>0.01 to 0.09	678	0.17<br>0.16 to 0.18	37	0.16<br>0.11 to<br>0.24	61	0.17<br>0.13 to<br>0.23	6	0.04<br>0.02 to 0.08
NVQ2/GCE O Level	2	0.01<br>0.00 to 0.07	878	0.19<br>0.18 to 0.21	58	0.22<br>0.17 to 0.27	88	0.21<br>0.17 to<br>0.25	6	0.03<br>0.02 to 0.08
NVQ1/CSE other grade	2	N/A	125	0.03<br>0.02 to<br>0.03	7	0.02<br>0.01 to<br>0.05	9	0.02<br>0.01 to 0.04	1	0.01<br>0.00 to 0.04
Foreign/other	0	N/A	95	0.02<br>0.02 to 0.02	4	0.01<br>0.00 to 0.03	9	0.02<br>0.01 to<br>0.04	2	0.01<br>0.00 to 0.04
No qualification	0	N/A	1060	0.22<br>0.21 to<br>0.24	30	0.10<br>0.07 to 0.15	57	0.13<br>0.10 to<br>0.17	2	0.01<br>0.00 to 0.03
**GHQ -12**										
**Item 1: Have you recently been able to concentrate on whatever you're doing?**										
Better than usual	3	0.05<br>0.01 to 0.15	123	0.03<br>0.02 to<br>0.04	10	0.04<br>0.02 to<br>0.07	15	0.04<br>0.02 to<br>0.06	3	0.02<br>0.01 to 0.06
Same as usual	32	0.61<br>0.46 to 0.74	3327	0.83<br>0.82 to 0.85	201	0.86<br>0.81 to 0.90	312	0.87<br>0.83 to 0.90	13<br>2	0.87<br>0.81 to 0.92
Less than usual	14	0.25<br>0.14 to 0.39	487	0.12<br>0.11 to 0.13	26	0.11<br>0.07 to 0.15	35	0.09<br>0.07 to 0.13	15	0.10<br>0.06 to 0.17
Much less than usual	5	0.09<br>0.04 to 0.21	63	0.01<br>0.01 to 0.02	0	N/A	0	N/A	1	0.01<br>0.00 to 0.04
**Item 2: Have you recently lost much sleep over worry?**										

Not at all	6	0.12 0.05 to 0.26	1123	0.28 0.27 to 0.30	65	0.27 0.21 to 0.34	98	0.26 0.22 to 0.32	38	0.25 0.18 to 0.33
No more than usual	24	0.45 0.31 to 0.60	2054	0.51 0.50 to 0.53	132	0.56 0.49 to 0.63	204	0.57 0.52 to 0.63	84	0.56 0.48 to 0.64
Rather more than usual	12	0.23 0.13 to 0.37	683	0.17 0.16 to 0.18	36	0.15 0.11 to 0.20	53	0.14 0.11 to 0.18	25	0.17 0.12 to 0.24
Much more than usual	12	0.19 0.10 to 0.33	151	0.03 0.03 to 0.04	5	0.02 0.01 to 0.05	8	0.02 0.01 to 0.04	4	0.02 0.01 to 0.06
**Item 3: Have you recently felt you were playing a useful part in things?**										
More so than usual	13	0.28 0.16 to 0.43	385	0.10 0.09 to 0.11	32	0.18 0.12 to 0.27	48	0.16 0.12 to 0.23	12	0.09 0.05 to 0.15
Same as usual	20	0.38 0.25 to 0.53	3163	0.79 0.78 to 0.81	191	0.76 0.68 to 0.83	291	0.77 0.71 to 0.82	12 4	0.80 0.72 to 0.86
Less useful than usual	15	0.26 0.15 to 0.40	351	0.08 0.08 to 0.09	11	0.05 0.03 to 0.08	20	0.05 0.03 to 0.08	14	0.10 0.06 to 0.17
Much less useful	6	0.09 0.03 to 0.20	91	0.02 0.02 to 0.03	3	0.01 0.00 to 0.04	3	0.01 0.00 to 0.03	1	0.01 0.00 to 0.05
**Item 4: Have you recently felt capable of making decisions about things?**										
More so than usual	4	0.07 0.03 to 0.19	278	0.07 0.06 to 0.08	16	0.06 0.04 to 0.10	22	0.06 0.04 to 0.09	8	0.07 0.03 to 0.13
Same as usual	41	0.78 0.65 to 0.88	3417	0.85 0.83 to 0.86	210	0.89 0.84 to 0.92	323	0.89 0.86 to 0.92	13 5	0.88 0.81 to 0.93
Less so than usual	7	0.10 0.04 to 0.21	273	0.07 0.06 to 0.08	11	0.04 0.02 to 0.08	17	0.04 0.03 to 0.07	8	0.05 0.03 to 0.10
Much less capable	2	0.04 0.01 to 0.17	43	0.01 0.01 to 0.01	1	0.01 0.00 to 0.04	1	0.00 0.00 to 0.02	0	N/A
**Item 5: Have you felt under constant strain recently?**										
Not at all	1	0.02 0.00 to 0.13	941	0.24 0.23 to 0.25	62	0.24 0.19 to 0.31	88	0.23 0.19 to 0.28	30	0.20 0.14 to 0.28
No more than usual	18	0.34 0.22 to 0.49	2201	0.55 0.53 to 0.57	129	0.55 0.47 to 0.62	206	0.57 0.52 to 0.63	93	0.63 0.54 to 0.70
Rather more than usual	20	0.39 0.26 to 0.54	726	0.18 0.17 to 0.19	42	0.19 0.14 to 0.26	60	0.17 0.13 to 0.22	26	0.16 0.11 to 0.22
Much more than usual	15	0.25 0.15 to 0.40	133	0.03 0.02 to 0.03	4	0.02 0.01 to 0.05	8	0.02 0.01 to 0.04	2	0.01 0.00 to 0.06
**Item 6: Have you recently felt you couldn't overcome your difficulties?**										
Not at all	10	0.20 0.11 to 0.34	1468	0.37 0.35 to 0.39	95	0.39 0.32 to 0.46	140	0.37 0.32 to 0.43	60	0.40 0.32 to 0.48
No more than usual	31	0.56 0.41 to 0.69	2082	0.52 0.50 to 0.53	127	0.55 0.47 to 0.62	197	0.56 0.50 to 0.61	81	0.54 0.46 to 0.62
Rather more than usual	10	0.18 0.09 to 0.32	361	0.09 0.08 to 0.10	16	0.07 0.04 to 0.11	24	0.07 0.04 to 0.10	7	0.05 0.02 to 0.09
Much more than usual	3	0.06 0.02 to 0.19	88	0.02 0.02 to 0.03	0	N/A	2	0.00 0.00 to 0.02	2	0.01 0.00 to 0.06
**Item 7: Have you recently been able to enjoy your normal day to day activities?**										
More so than usual	5	0.08 0.03 to 0.20	218	0.06 0.05 to 0.07	20	0.13 0.07 to 0.22	24	0.09 0.06 to 0.16	11	0.08 0.04 to 0.15
Same as usual	30	0.58 0.43 to 0.71	3112	0.78 0.76 to 0.79	184	0.74 0.66 to 0.81	288	0.77 0.71 to 0.82	12 4	0.82 0.75 to 0.88
Less so than usual	10	0.17	542	0.13	29	0.11	45	0.12	13	0.08

		0.09 to 0.31		0.12 to 0.14		0.08 to 0.16		0.09 to 0.15		0.05 to 0.14
Much less than usual	9	0.17 0.08 to 0.30	137	0.03 0.03 to 0.04	5	0.02 0.01 to 0.05	6	0.02 0.01 to 0.04	3	0.02 0.01 to 0.06
**Item 8: Have you recently been able to face up to your problems?**										
More so than usual	2	0.04 0.01 to 0.17	186	0.05 0.04 to 0.06	10	0.07 0.03 to 0.16	15	0.06 0.03 to 0.12	8	0.06 0.03 to 0.13
Same as usual	45	0.83 0.69 to 0.91	3411	0.86 0.85 to 0.87	213	0.89 0.81 to 0.94	323	0.90 0.84 to 0.93	137	0.90 0.83 to 0.94
Less able than usual	7	0.13 0.06 to 0.26	312	0.08 0.07 to 0.08	9	0.04 0.02 to 0.08	17	0.05 0.03 to 0.07	5	0.03 0.01 to 0.07
Much less able	0	N/A	43	0.01 0.01 to 0.01	0	N/A	0	N/A	1	0.01 0.00 to 0.05
**Item 9: Have you recently been feeling unhappy and depressed?**										
Not at all	14	0.26 0.16 to 0.41	1583	0.40 0.38 to 0.41	117	0.49 0.42 to 0.56	167	0.46 0.40 to 0.52	64	0.42 0.34 to 0.50
No more than usual	21	0.38 0.25 to 0.53	1699	0.43 0.42 to 0.45	88	0.40 0.33 to 0.48	141	0.42 0.36 to 0.47	73	0.49 0.41 to 0.58
Rather more than usual	19	0.35 0.23 to 0.50	545	0.13 0.12 to 0.15	24	0.10 0.07 to 0.15	42	0.11 0.08 to 0.15	11	0.07 0.04 to 0.13
Much more than usual	0	N/A	131	0.03 0.03 to 0.04	2	0.01 0.00 to 0.03	4	0.01 0.00 to 0.03	3	0.02 0.01 to 0.06
**Item 10: Have you recently been losing confidence in yourself?**										
Not at all	15	0.29 0.17 to 0.44	1682	0.42 0.40 to 0.44	119	0.52 0.45 to 0.59	173	0.49 0.43 to 0.55	69	0.45 0.37 to 0.54
No more than usual	26	0.49 0.35 to 0.63	1689	0.43 0.41 to 0.44	95	0.41 0.35 to 0.48	145	0.41 0.36 to 0.47	68	0.45 0.37 to 0.53
Rather more than usual	13	0.22 0.13 to 0.36	476	0.12 0.11 to 0.13	13	0.05 0.03 to 0.09	29	0.08 0.06 to 0.11	14	0.10 0.06 to 0.17
Much more than usual	0	N/A	112	0.03 0.02 to 0.04	4	0.02 0.01 to 0.04	7	0.02 0.01 to 0.04	0	N/A
**Item 11: Have you recently been thinking of yourself as a worthless person?**										
Not at all	32	0.60 0.45 to 0.73	2561	0.64 0.63 to 0.66	171	0.74 0.68 to 0.80	249	0.71 0.65 to 0.75	101	0.67 0.58 to 0.74
No more than usual	15	0.29 0.17 to 0.44	1069	0.27 0.26 to 0.29	52	0.23 0.18 to 0.29	90	0.26 0.21 to 0.31	42	0.27 0.20 to 0.35
Rather more than usual	7	0.11 0.05 to 0.24	244	0.06 0.05 to 0.07	4	0.02 0.01 to 0.04	9	0.02 0.01 to 0.05	7	0.05 0.02 to 0.11
Much more than usual	0	N/A	80	0.02 0.02 to 0.02	3	0.01 0.00 to 0.04	5	0.01 0.00 to 0.03	1	0.01 0.00 to 0.05
**Item 12: Have you recently been feeling reasonably happy, all things considered?**										
More so than usual	11	0.19 0.10 to 0.32	388	0.10 0.09 to 0.11	27	0.15 0.10 to 0.23	40	0.13 0.09 to 0.19	10	0.07 0.04 to 0.12
About same as usual	29	0.54 0.40 to 0.68	3123	0.79 0.77 to 0.80	189	0.79 0.71 to 0.85	287	0.79 0.74 to 0.84	131	0.86 0.80 to 0.91
Less so than usual	14	0.27 0.16 to 0.42	368	0.09 0.08 to 0.10	12	0.05 0.03 to 0.08	24	0.06 0.04 to 0.09	9	0.06 0.03 to 0.11
Much less than usual	0	N/A	78	0.02 0.02 to 0.03	3	0.01 0.00 to 0.04	3	0.01 0.00 to 0.03	1	0.01 0.00 to 0.05

**Presence of probable mental ill health**										
No evidence of probable MIH	9	0.19 0.10 to 0.34	2247	0.51 0.49 to 0.52	147	0.57 0.51 to 0.64	226	0.58 0.53 to 0.63	86	0.52 0.44 to 0.60
Less than optimal MIH	23	0.40 0.27 to 0.54	955	0.22 0.20 to 0.23	53	0.19 0.15 to 0.24	79	0.19 0.15 to 0.23	48	0.29 0.22 to 0.36
MIH	22	0.41 0.27 to 0.56	1237	0.28 0.26 to 0.29	59	0.24 0.18 to 0.30	92	0.23 0.19 to 0.28	29	0.20 0.14 to 0.27

Weighted proportion (WP) with the corresponding 95% Confidence Intervals (CI).

Key: MP: Member of Parliament Sample; EN: English Population (HSE 2014); CM: Corporate Managers (HSE 2014); AM: All managers (HSE 2014); HIG: high-income group (HSE 2014).

For peer review only

4. TABLE S3

Table S3. Crude and adjusted associations of mental health in relation to job status (having a job outside the parliament vs. not) of members of the parliament

GHQ-12 Items (n=146)	Crude		Adjusted±	
	**OR**	**95%CI**	**OR**	**(95% CI)**
Have you recently been able to concentrate on whatever you're doing?	0·6	0.23 to 1.57	0.74	0.27 to 2.04
Have you recently lost much sleep over worry?	0·64	0.26 to 1.58	0.73	0.28 to 1.90
Have you recently felt you were playing a useful part in things?	1.52	0.70 to 3.28	1.62	0.70 to 3.74
Have you recently felt capable of making decisions about things?	0·98	0.37 to 2.56	1.17	0.42 to 3.27
Have you felt under constant strain recently?	0·59	0.26 to 1.34	0.71	0.32 to 1.59
Have you recently felt you couldn't overcome your difficulties?	0·74	0.36 to 1.50	0.87	0.41 to 1.85
Have you recently been able to enjoy your normal day to day activities?	1.01	0.43 to 2.37	0.96	0.36 to 2.57
Have you recently been able to face up to your problems	1.04	0.37 to 2.93	0.98	0.36 to 2.69
Have you recently been feeling unhappy and depressed?	0·66	0.31 to 1.41	0.82	0.35 to 1.92
Have you recently been losing confidence in yourself?	1.02	0.37 to 2.69	1.29	0.46 to 3.60
Have you recently been thinking of yourself as a worthless person?	1.01	0.41 to 2.43	1.2	0.45 to 3.21
Presence of Common Mental Disorders	0.77	0.47 to 1.26	0.82	0.49 to 1.36
	**MD**	**95%CI**	**MD**	**95%CI**
**Total Score of GHQ to 12**	-.61	-3.06 to 1.84	-0·07	-2.44 to 2.31

Crude and Adjusted Odds Ratio (ORs) and Mean Difference (MD) with corresponding 95% Confidence Intervals (95% CI). Inverse probability weights were used with reference to the total number of the members of the parliament. All models were adjusted for age, sex and educational status

For peer review only

STROBE Statement—Checklist of items that should be included in reports of ***cross-sectional studies***

Item	Item No	Recommendation	Page No
**Title and abstract**	1	(*a*) Indicate the study's design with a commonly used term in the title or the abstract	1
		(*b*) Provide in the abstract an informative and balanced summary of what was done and what was found	3
**Introduction**			
Background/rationale	2	Explain the scientific background and rationale for the investigation being reported	4-5
Objectives	3	State specific objectives, including any prespecified hypotheses	5
**Methods**			
Study design	4	Present key elements of study design early in the paper	5
Setting	5	Describe the setting, locations, and relevant dates, including periods of recruitment, exposure, follow-up, and data collection	5-6
Participants	6	(*a*) Give the eligibility criteria, and the sources and methods of selection of participants	5
Variables	7	Clearly define all outcomes, exposures, predictors, potential confounders, and effect modifiers. Give diagnostic criteria, if applicable	7
Data sources/ measurement	8*	For each variable of interest, give sources of data and details of methods of assessment (measurement). Describe comparability of assessment methods if there is more than one group	6-7
Bias	9	Describe any efforts to address potential sources of bias	5-6
Study size	10	Explain how the study size was arrived at	5, 8
Quantitative variables	11	Explain how quantitative variables were handled in the analyses. If applicable, describe which groupings were chosen and why	8-9
Statistical methods	12	(*a*) Describe all statistical methods, including those used to control for confounding	8-9
		(*b*) Describe any methods used to examine subgroups and interactions	8-9
		(*c*) Explain how missing data were addressed	8-9
		(*d*) If applicable, describe analytical methods taking account of sampling strategy	8-9
		(*e*) Describe any sensitivity analyses	8-9
**Results**			
Participants	13*	(a) Report numbers of individuals at each stage of study—eg numbers potentially eligible, examined for eligibility, confirmed eligible, included in the study, completing follow-up, and analysed	9
		(b) Give reasons for non-participation at each stage	n.a.
		(c) Consider use of a flow diagram	n.a.
Descriptive data	14*	(a) Give characteristics of study participants (eg demographic, clinical, social) and information on exposures and potential confounders	9
		(b) Indicate number of participants with missing data for each variable of interest	n.a.
Outcome data	15*	Report numbers of outcome events or summary measures	9-12
Main results	16	(*a*) Give unadjusted estimates and, if applicable, confounder-adjusted estimates and their precision (eg, 95% confidence interval). Make clear	10-12

For peer review only - http://bmjopen.bmj.com/site/about/guidelines.xhtml

		which confounders were adjusted for and why they were included	
		(*b*) Report category boundaries when continuous variables were categorized	10-12
		(*c*) If relevant, consider translating estimates of relative risk into absolute risk for a meaningful time period	N.a.
Other analyses	17	Report other analyses done—eg analyses of subgroups and interactions, and sensitivity analyses	12-13
**Discussion**			
Key results	18	Summarise key results with reference to study objectives	13-14
Limitations	19	Discuss limitations of the study, taking into account sources of potential bias or imprecision. Discuss both direction and magnitude of any potential bias	14-15
Interpretation	20	Give a cautious overall interpretation of results considering objectives, limitations, multiplicity of analyses, results from similar studies, and other relevant evidence	16
Generalisability	21	Discuss the generalisability (external validity) of the study results	15-17
**Other information**			
Funding	22	Give the source of funding and the role of the funders for the present study and, if applicable, for the original study on which the present article is based	18

*Give information separately for exposed and unexposed groups.

Note: An Explanation and Elaboration article discusses each checklist item and gives methodological background and published examples of transparent reporting. The STROBE checklist is best used in conjunction with this article (freely available on the Web sites of PLoS Medicine at http://www.plosmedicine.org/, Annals of Internal Medicine at http://www.annals.org/, and Epidemiology at http://www.epidem.com/). Information on the STROBE Initiative is available at www.strobe-statement.org.

For peer review only
